# Palladium-catalysed cyclisation of alkenols: Synthesis of oxaheterocycles as core intermediates of natural compounds

**DOI:** 10.3762/bjoc.10.216

**Published:** 2014-09-03

**Authors:** Miroslav Palík, Jozef Kožíšek, Peter Koóš, Tibor Gracza

**Affiliations:** 1 Department of Organic Chemistry Slovak University of Technology, Radlinského 9, SK-812 37 Bratislava, Slovakia; 2 Department of Physical Chemistry, Slovak University of Technology, Radlinského 9, SK-812 37 Bratislava, Slovakia; 3 Georganics Ltd., Koreničova 1, SK-811 03 Bratislava, Slovakia

**Keywords:** alkenols, cycloetherification, homogeneous catalysis, natural products, palladium

## Abstract

The study of Pd-catalysed cyclisation reactions of alkenols using different catalytic systems is reported. These transformations affect the stereoselective construction of mono- and/or bicyclic oxaheterocyclic derivatives depending on a starting alkenol. The substrate scope and proposed mechanism of Pd-catalysed cyclisation reactions are also discussed. Moreover, the diastereoselective Pd-catalysed cyclisation of appropriate alkenols to tetrahydrofurans and subsequent cyclisation provided properly substituted 2,5-dioxabicyclo[2.2.1]heptane and 2,6-dioxabicyclo[3.2.1]octane, respectively. Such bicyclic ring subunits are found in many natural products including ocellenynes and aurovertines.

## Introduction

**Figure 1:**
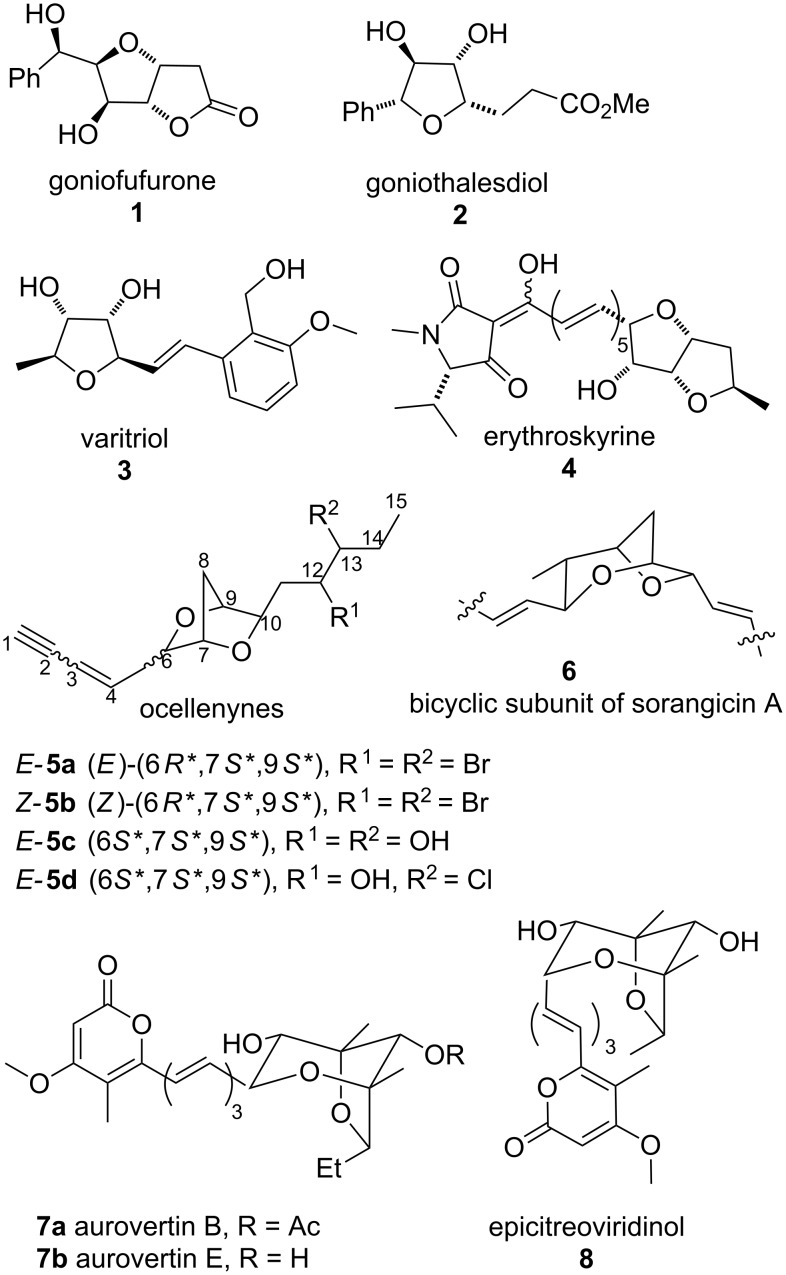
Examples of naturally occurring tetrahydrofurans.

Oxaheterocycles of various sizes are found in many different biologically active compounds. Particularly, substituted tetrahydrofuran units are present in a large branch of natural products that display interesting biological properties, such as goniofufurone **1** [1], goniothalesdiol **2** [2], varitriol **3** [3], erythroskyrine **4** [4–5], ocellenynes **5** [6–7], sorangicin A **6** [8], aurovertins **7** [9–12] and epicitreoviridinol **8** [13] (Figure 1).

Over the last decades, an enormous work has been devoted to find an efficient stereoselective route to variously substituted tetrahydrofurans [14–15]. Among many described transformations, the metal-catalysed carboetherification reactions [16–18] and intramolecular oxycarbonylations of alkenes [19–21] are of particular importance. Although, many of these synthetic routes have showed their potential, there is still an area for improving the scope and stereocontrol of the new synthetic construction of substituted tetrahydrofurans.

**Scheme 1:**
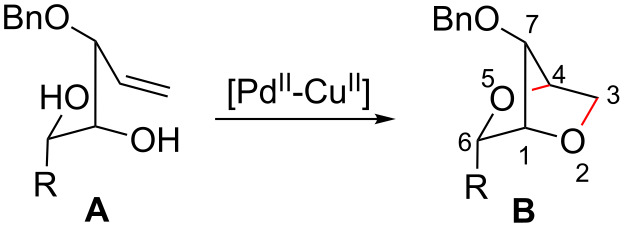
PdCl_2_/CuCl_2_-catalysed bicyclisation of unsaturated polyols [22].

Recently, we have described a novel type of PdCl_2_/CuCl_2_-catalysed bicyclisation reaction of α-*O*-benzyl-protected sugar-derived alkenitols **A**, that provided 2,5-dioxabicyclo[2.2.1]heptanes **B** with high 1,4-*threo*-selectivity (Scheme 1) [22–23]. In this process, the terminal carbon–carbon double bond is bis-*O*-functionalised with two hydroxy groups by sequential intramolecular–intramolecular reaction.

Based on our continuous interest in the palladium-catalysed cyclisation reactions and their applications in natural product syntheses [24–25], we have decided to explore the substrate scope and the limitations of this transformation. With this aim, we detailed the synthesis of a number of alkene alcohols, and described different catalytic systems in the Pd-catalysed cyclisation reaction. Additionally, we have also outlined the synthetic approach to substituted 2,5-dioxabicyclo[2.2.1]heptane and 2,6-dioxabicyclo[3.2.1]octane rings. Such substituted bicyclic rings are of further interest as they are the core substructures in a number of marine derived metabolites, including ocellenynes and sorangicin A.

## Results and Discussion

### Synthesis of starting materials

Palladium-catalysed cyclisations are substrate selective reactions. In most cases, the literature known cyclisation using similar substrate (with even small change in its substructure) can lead in different product formation. Although, there are several known rules-reactions (β-hydride elimination, η^3^-complex formation...) which are applicable to predict the behaviour of the used substrate in the Pd-catalysed reaction, there are cases where the results of such reactions still remain on experimental findings.

While the cyclisation reactions of alkenols have been relatively well described in the literature, only less attention was given to the reactions of unsaturated polyols. However, such cyclisations can provide a variety of products which are useful intermediates in many natural product syntheses. With this aim, we have designed syntheses of several substrates bearing different double bonds and substituents to cover certain possibilities for Pd-catalysed cyclisation screening.

**Figure 2:**
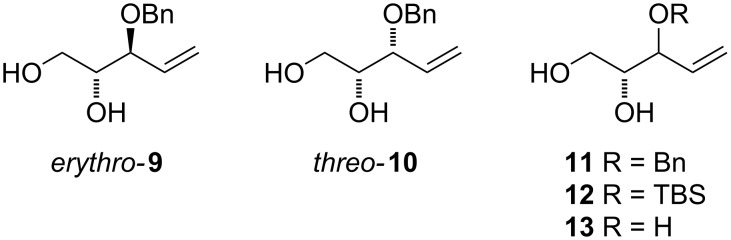
Structures of C_5_-alkenitols.

At first, easily accessible C_5_-alkenitols (Figure 2) were chosen as simplest suitable substrates for screening the optimal reaction conditions of the previously described bicyclisation reaction. Thus, the known triols *erythro*-**9** [26] and *threo*-**10** [27] were prepared from divinylcarbinol using asymmetric epoxidation [28–29]. Diastereomeric mixtures of 3-*O*-benzyl **11**, 3-*O*-silyl-protected **12** and fully unprotected triol **13** was prepared starting from 1,2-*O*-isopropylidene-D-glyceraldehyde using described procedures [30].

**Scheme 2:**
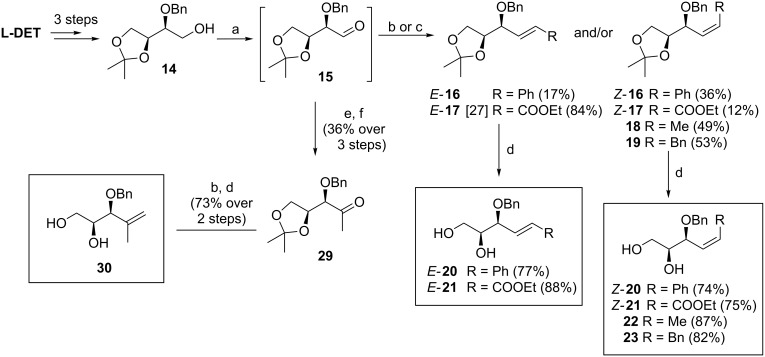
Synthesis of alkenols **20**-**23** and **30**. Reagents and conditions: a) lit. [31] (COCl)_2_, DMSO, Et_3_N, CH_2_Cl_2_, −78 °C to rt, 2 h; b) Phosphonium salt, BuLi, THF, 0 °C to rt, overnight, MPLC; c) lit. [32] (EtO)_2_POCH_2_CO_2_Et, NaH, THF, 0 °C to rt, 3 h; d) 60% AcOH, 60 °C, 3 h; e) MeMgCl, Et_2_O, 0 °C to rt, 1 h; f) Dess–Martin periodinane, CH_2_Cl_2_, 0 °C, 1 h. L-DET = L-dimethyl tartrate.

The preparation of substrates **20**–**23** having a symmetrically disubstituted C–C double bond is depicted in Scheme 2. The synthesis started from known threose **15** [31] followed by a common synthetic sequence comprising the olefination reaction and the hydrolysis of the acetonide protecting group. Thus, Horner–Wadsworth–Emmons olefination of aldehyde **15** furnished the corresponding separable mixture of *Z* and *E* alkenes **16** and **17**. In the case of utilising stabilised phosphorane ylides, the Wittig reaction provided only *Z* alkenes **18** and **19**. Following acidic hydrolysis provided α-*O*-benzyl substrates **20**–**23** in good yields. Additionally, the synthesis of substrate **30** with 1,1-disubstituted C–C double bond was accomplished in 3 steps. The addition of methylmagnesium chloride to previously prepared threose **15**, followed by Dess–Martin oxidation of the secondary alcohol gave methylketone **29**. Subsequent Wittig olefination using (methylidene)triphenylphosphorane ylide and final hydrolysis of the acetonide provided the desired C_5_-substrate **30**.

**Scheme 3:**
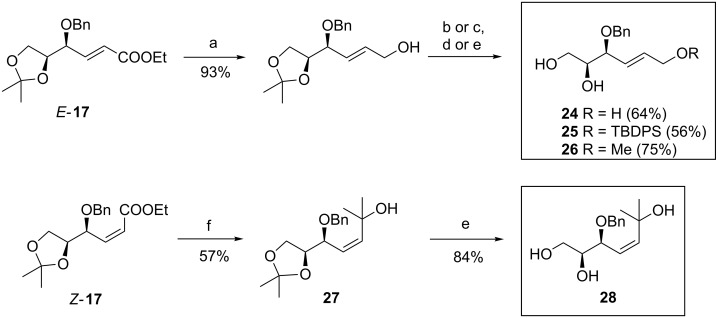
Synthesis of alkenols **24**–**26** and **28**. Reagents and conditions: a) lit. [32] DIBAL-H, CH_2_Cl_2_; b) TBDPSCl, imidazole, CH_2_Cl_2_, rt, overnight; c) NaH, THF then MeI, rt, overnight; d) FeCl_3_·6H_2_O, CHCl_3_, rt, 1 h (for **25**); e) 60% AcOH, 60 °C, 3 h; f) MeLi, Et_2_O, −78 °C, 1 h. TBDPSCl = *tert*-butyldiphenylsilyl chloride.

The synthesis of substrates **24**–**28** bearing an allylic hydroxy group is pictured in Scheme 3. At first, the ester group of previously prepared intermediate *E*-**17** was reduced using DIBAL-H [32] providing the known allylic alcohol in very good yield. Following protection of the primary alcohol yielded fully protected alkene-tetraol and subsequent chemoselective removal of the acetonide protecting group in one pot led to substrates **24**–**26**. The synthesis of substrate **28** bearing a tertiary allylic alcohol was performed in a two-step sequence. The addition of methyllithium to ester *Z*-**17** and following deprotection of the corresponding alcohol **27** with aqueous acetic acid afforded tetraol **28** in good yield.

**Scheme 4:**
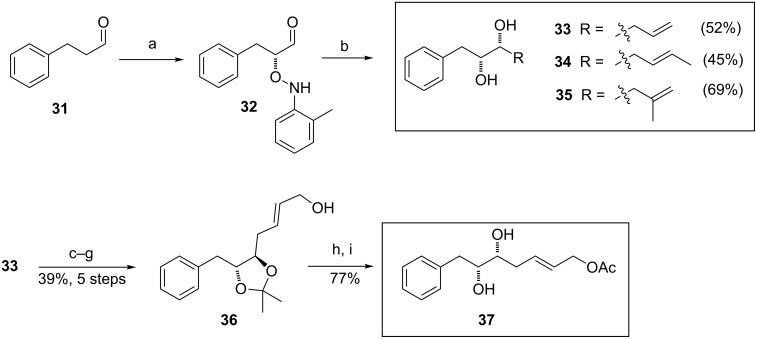
Synthesis of substrates **33–35**, **37**. Reagents and conditions: a) lit. [33] L-proline (0.25 equiv), 2-nitrosotoluene, CHCl_3_, −18 °C; b) RMgCl, CeCl_3_·2LiCl, THF, −78 °C to rt, overnight; c) acetone, PTSA, 3 h, rt; d) OsO_4_ (0.01 equiv), NMO (2 equiv), pyridine, 5 d, rt; e) NaIO_4_, MeOH/H_2_O, 3 h, rt; f) NaH, (EtO)_2_POCH_2_CO_2_Et, THF, −20 °C to rt, 15 min; g) DIBAL-H, CH_2_Cl_2_, −30 °C to −10 °C, 45 min; h) Ac_2_O, pyridine, CH_2_Cl_2_, 3 h, rt; i) AcOH/H_2_O, 3 h, 60 °C. PTSA = *p*-toluenesulfonic acid, NMO = *N*-methylmorpholine *N*-oxide, DIBAL = diisobutylaluminium hydride.

Substrates *syn*-diols **33**–**35** (not bearing an α-*O*-protected group) were prepared in 2 steps starting from the aldehyde **31** using the Yamamoto’s [33] sequential *O*-nitrosoaldol and Grignard addition process using different reagents (Scheme 4).

Thus, L-proline-catalysed oxidation of **31** with 2-nitrosotoluene gave the optically pure *O*-selective nitrosoaldol product **32**, which underwent a reaction with the corresponding Grignard reagent in the presence of CeCl_3_·2LiCl providing diols **33**–**35** in good overall yields and high diastereoselectivity (d.r. >20:1).

Additionally, allylic acetate **37** was obtained starting from **33** in a seven-step sequence in 30% overall yield. The acetonisation of hydroxy groups of the previously prepared diol **33**, followed by OsO_4_ dihydroxylation of the C–C double bond provided the corresponding diol in good yield. The resulting vicinal diol was then cleaved by sodium periodate to the corresponding aldehyde, which was immediately subjected to a Horner–Wadsworth–Emmons olefination using diethyl carbethoxyethylidenephopsphonate. Reduction of the resultant ester with DIBAL-H in dichloromethane afforded partially protected triol **36** in 39% yield over five steps. Finally, acetylation of the primary hydroxy group and subsequent removal of the acetonide provided the target compound **37** in good yield (77%).

**Scheme 5:**
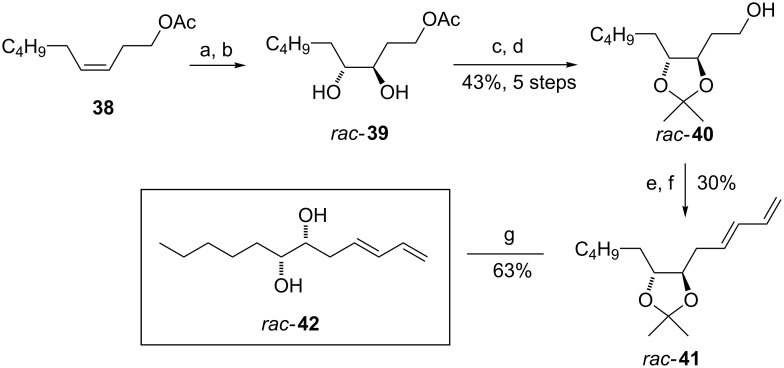
Synthesis of *rac*-**42**. Reagents and conditions: a) MCPBA, CH_2_Cl_2_, 0 °C to rt, 45 min; b) TFA, H_2_O, THF, 60 °C overnight, c) acetone, PTSA, 3 h, rt; d) NaOMe, MeOH, 48 h, rt, 43% over 5 steps; e) (COCl)_2_, DMSO, Et_3_N, CH_2_Cl_2_, −78 °C to rt, 2 h; f) diethyl allylphosphonate, BuLi, THF, 0 °C to rt, overnight, 30% over 2 steps; g) 60% AcOH, 60 °C, 3 h, 63%. MCPBA = *m*-chloroperbenzoic acid, TFA = trifluoacetic acid, PTSA = *p*-toluenesulfonic acid.

Synthesis of the similar substrate *rac*-**42** having two conjugated double bonds is shown in Scheme 5. The synthesis started from the known acetate **38**, which was obtained by acetylation of commercially available non-3-ene-1-ol [34]. Epoxidation of acetate **38** with MCPBA in dichloromethane and subsequent acidic epoxide hydrolysis produced the *syn*-diol *rac***-39**. The following protection of diol *rac***-39** as its acetonide and the primary hydroxy group deprotection using sodium methoxide afforded alcohol *rac***-40** in good yield (43% over 5 steps). Next, Swern oxidation of the primary hydroxy group provided the aldehyde, which was then transformed to diene derivative *rac***-41** by Wadsforth–Emmons olefination using diethyl allylphosphonate. Final deprotection of the hydroxy groups furnished *rac***-42** in 63% yield.

**Figure 3:**
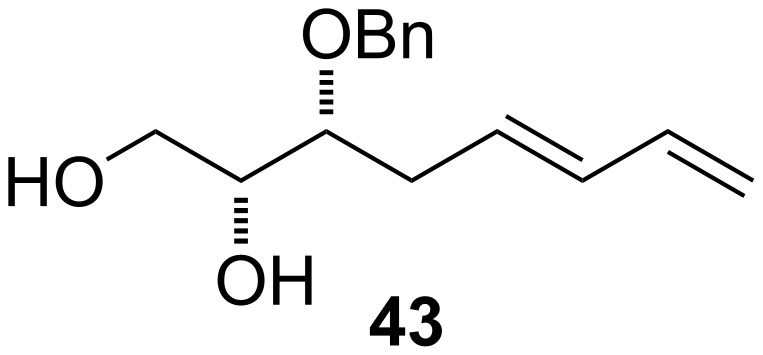
Structure of **43**.

In addition, enantiomerically pure substrate **43** was synthetised from D-glucose in 11 steps according to a protocol of Szewczyk [35] (Figure 3).

### Pd-catalysed cyclisations of unsaturated polyols

**Scheme 6:**
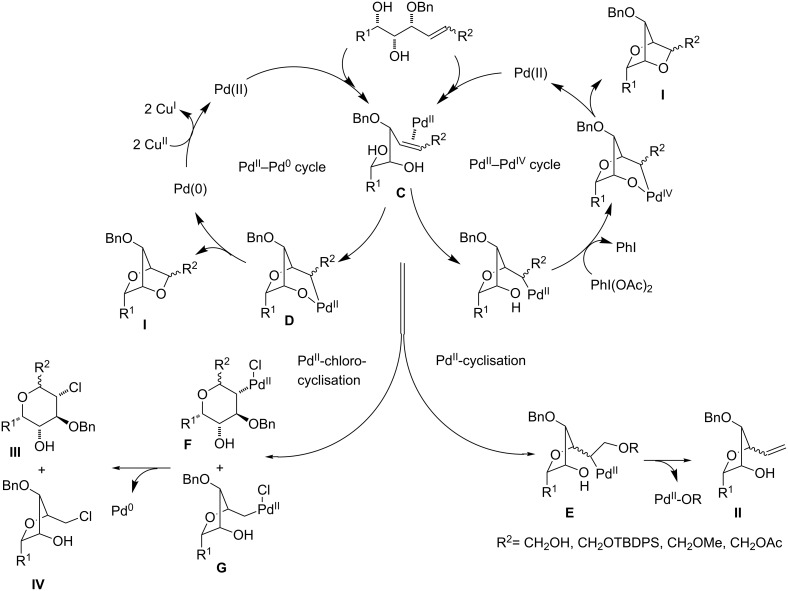
Suggested mechanisms for Pd^II^–Pd^0^, Pd^II^–Pd^IV^ and Pd^II^-chloro/cyclisation of unsaturated polyols.

Prepared substrates were then subjected to the Pd-catalysed transformation under several reaction conditions. At first, we tried the reaction conditions which were recently developed for the bicyclisation of α-*O*-benzyl-protected polyols bearing a terminal alkene moiety [22]. Thus, the reactions incorporating the Pd^II^–Pd^0^ catalytic cycle (Scheme 6) were carried out using PdCl_2_ (0.1 equiv) as a catalyst, CuCl_2_ (3 equiv) as a reoxidant, NaOAc (3 equiv) as a buffer in AcOH at room temperature (Table 1, Method A, see Supporting Information File 1 for full experimental data).

**Table 1:** Pd-Catalysed cyclisations of unsaturated polyols.

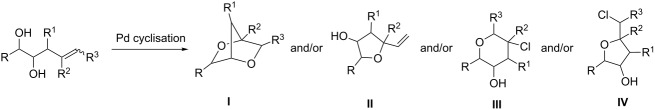				

Entry	Substrate	Reaction conditions^a^	Product(s)	Yield (%)

1	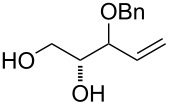 **11**	Method A	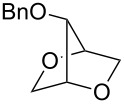 **44**	79 [23]

2	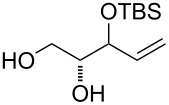 **12**	Method A Method C	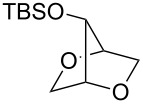 **45**	63 40

3	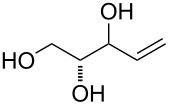 **13**	Method A	Complex mixture	

4	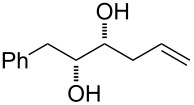 **33**	Method A Method B	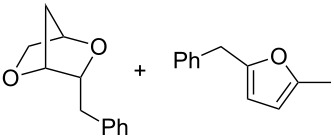 **51** + **52**	15 (**51**), 25 (**52**) 65 (**52**)

5	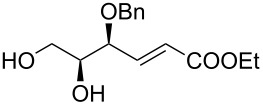 *E***-21**	Method A	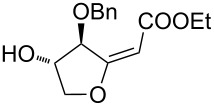 **46**	30

6	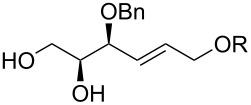 **24–26**	Method A	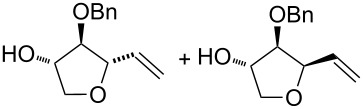 **47** + **48**	66 (**47**/**48**, 5:3)

7	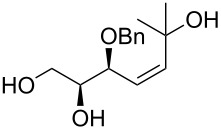 **28**	Method A	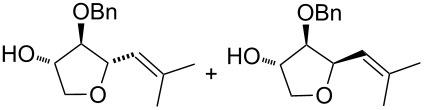 **49** + **50**	54 (**49**/**50**, 5:3)
8	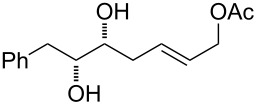 **37**	Method A Method B Pd(PPh_3_)_4_^b^	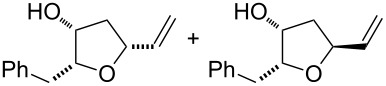 **56** + **57**	70 (**56/57**, 1:3) 69 (**56/57**, 1:3) 84 (**56/57**, 1:3)

9	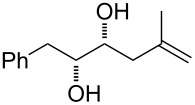 **35**	Method A	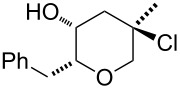 **53**	33

10	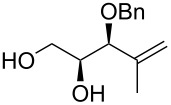 **30**	Method A Method B	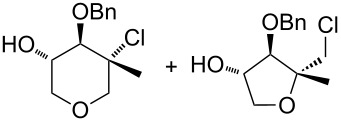 **54** + **55**	38 (**54**), 35 (**55**) 70 (**55**)

11	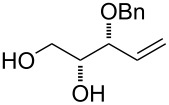 *threo*-**9**	Method B	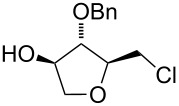 **58**	78

^a^Method A: PdCl_2_ (0.1 equiv), CuCl_2_ (3 equiv), NaOAc (3 equiv), AcOH, rt.; method B: PdCl_2_(MeCN)_2_ (0.1 equiv), BuLi (2 equiv), CuCl_2_ (3 equiv), LiCl (3 equiv), THF, rt; method C: Pd(OAc)_2_ (0.1 equiv), PhI(OAc)_2_ (2 equiv), Me_4_N^+^Cl^−^ (1 equiv), NaOAc (1 equiv), AcOH, rt. ^b^Lit. [36–38] Pd(PPh_3_)_4_ (0.1 equiv), THF, rt.

It is clear, that the chemoselectivity of the cyclisation reaction directly correlates to the nature of the C=C bond of the substrate. Also, the configuration and the position of participating substituents have immense influence on the reaction output and the obtained results showed that the cyclisation reactions can progress through four different transformation pathways, yielding various types of products (**I**, **II**, **III** and **IV**).

Under these conditions (method A) the reactions of the simplest alkenols having a terminal alkene moiety and those with an α-*O*-protected allylic system, i.e., **11** [23] (Table 1, entry 1) and **12** (Table 1, entry 2) provided corresponding bicycles of the type **I**. Likewise, the alkenediol **33** without α-allylic hydroxy group provided bicyclic product **51**, however, as a minor product in only 15% yield along with the furan compound **52** (Table 1, entry 4). The furan derivative **52** in this reaction was probably formed through a monocyclisation, followed by β-*H*^−^-elimination and aromatisation. In the case of substrate **13** having an unprotected α-hydroxy group, the reaction provided only a mixture of unidentified products (Table 1, entry 3). These results are consistent with previous observations and it is evident that a protection of the α-allylic hydroxy function is required for a successful bicyclisation reaction.

Next, we have also examined the compatibility of substrates having a symmetrically disubstituted C=C bond in the cyclisation reactions. Unfortunately, butadienes *rac*-**42** and **43** underwent uncontrollable transformations under these conditions providing a complex mixture of products. Similarly, the transformations of substrates **20**, *Z*-**21**, **22** and **34** failed, while the reaction of *E*-**21** (Table 1, entry 5) afforded surprisingly tetrahydrofuran derivative **46** as a product of a Wacker-type cyclisation.

Interestingly, the reactions of alkenols having an additional allylic OR group provided only products of type **II**. Thus, diastereomeric mixtures of vinyltetrahydrofurans **47**, **48** (Table 1, entry 6), **49**, **50** (Table 1, entry 7) and **56**, **57** (Table 1, entry 8) were formed starting from alkenols **24**–**26**, **28** and **37**. Formation of these products (type **II**) in this type of Pd^II^-catalysed cyclisation [21,39–43] possibly involved an intramolecular Wacker-type reaction to form Pd^II^-intermediate **E** and subsequent regeneration of the Pd^II^-catalyst via cleavage of the C–OR bond (Scheme 6).

**Figure 4:**
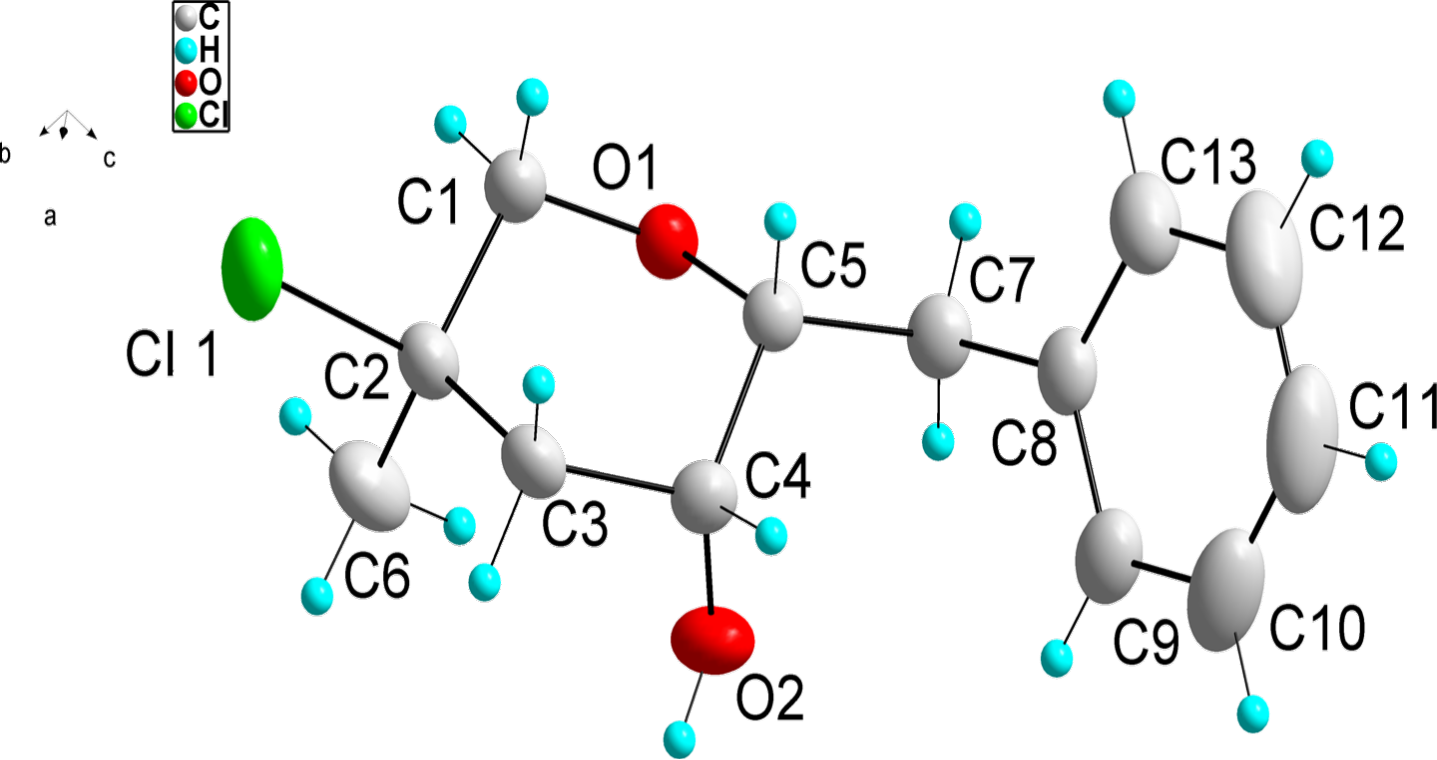
An ORTEP [44] view of crystal and molecular structure of **53**.

Surprisingly, the reaction of 1,1-disubstituted alkenes **30**, **35** (Table 1, entries 10 and 9) provided only chlorinated tetrahydropyran (type **III**) and tetrahydrofuran (type **IV**) derivatives. The chloroderivatives **III** and **IV** were probably formed from σ-alkyl Pd^II^-complexes **F** and **G** by reductive elimination of Pd^0^ (Scheme 6). Additionally, the X-ray analysis [44] of **53** confirmed the absolute configuration and structure of the six-membered heterocycle (Figure 4).

Recently, Wolfe reported a tetrahydrofuran-forming reaction via Pd-catalysed carboetherification [45–49] under strong basic conditions in the presence of a phosphine ligand. In order to enhance the ligand affinity of the hydroxy group in the formation of σ-palladium^II^-complex **D** (Scheme 6), we have decided to adopt the described conditions and to examine the transformation of alkenols in the presence of PdCl_2_(MeCN)_2_, butyllithium and LiCl (method B). Unfortunately, these experiments in most cases did not afford any cyclisation products and reactions of non-terminal olefinic substrates **20**–**23**, **34**, *rac*-**42** and **43** provided only a complex mixture of inseparable products. However, terminal olefins **30** and *threo*-**9** underwent chlorocyclisation most probably due to the presence of an excess of chloride anions (Table 1, entries 10 and 11). Interestingly, this chlorocyclisation reaction proceeded with high *trans*-diastereoselectivity, which is in accordance with Wolfe´s TS model [17–18]. In both cases, only 2,3-*trans* diastereomers **55** and **58** were isolated in good yields. In addition, this reaction represents a new synthetic access to the 3-hydroxy-2,3-*trans*-tetrahydrofuran skeleton and is complementary to the known X^+^-mediated cyclisation methodology producing exclusively 2,3-*cis*-diastereomer [14–15,50–51].

Based on the published findings, we have also examined the cyclisation reactions incorporating the Pd^II^–Pd^IV^ catalytic cycle [52–53] (Scheme 6). The experiments were carried out using Pd(OAc)_2_ salt as a catalyst, PhI(OAc)_2_ as reoxidant, AcONa and Me_4_N^+^Cl^−^ as buffer in AcOH (method C). Unfortunately, all reactions and their modifications (temperature, solvents: AcOH, AcOH–H_2_O, NMP, DMF, MeOH, THF, Et_2_O, DCM, CHCl_3_) did not provide cyclisation products and only complex mixtures of β-*H*^−^-elimination and consequential products were observed. Only one exception to previously unpleasant findings was a reaction of *O*-silyl-protected triol **12**, which provided the bicycle **45** but only in a decreased yield of 40% (Table 1, entry 2).

**Scheme 7:**
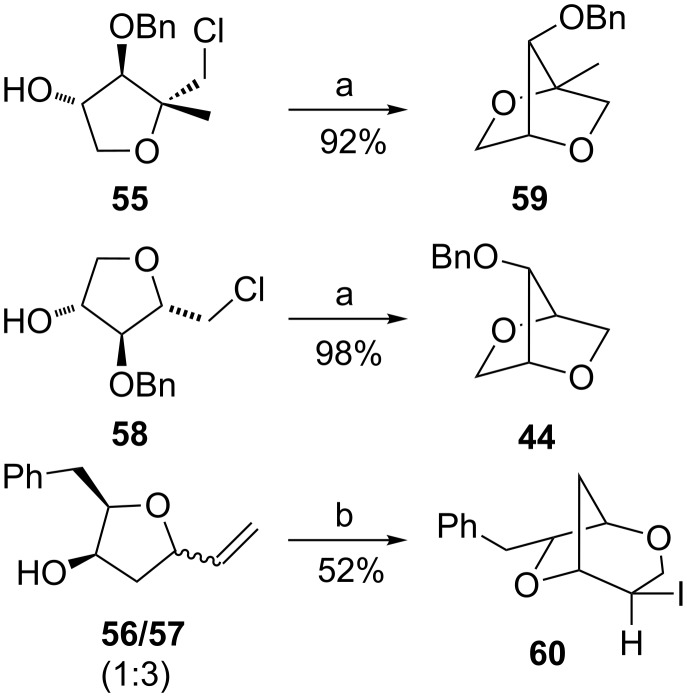
Bicyclisation of **55**–**58**. Reagents and conditions: a) NaH, DMF, 50 °C, 2 h; b) I_2_, CH_3_CN, rt, overnight.

To show the usefulness of such cyclisation products, we have investigated the possibility of employing prepared tetrahydrofuran derivatives bearing suitable moieties in the next cyclisation step (Scheme 7).

Gratifyingly, the chloromethyltetrahydrofurans **55** and **58** were both transformed into the bicyclic products **59** and **44** by treatment with sodium hydride in DMF. Also, this transformation step has approved the relative configurations of substituents on the tetrahydrofuran ring. Interestingly, an iodo-cyclisation reaction of a 1:3 diastereomeric mixture of vinyltetrahydrofurans **56** and **57** in acetonitrile provided only one corresponding product derived from **56**. Thus, pure (4*R*,7*R*)-4,7-disubstituted 2,6-dioxabicyclo[3.2.1]octane **60** was isolated in 52% yield. The *trans* arrangement of the substituents at C4/C7 in the product of the 6-*endo*-trig cyclisation was determined by means of ^1^H NMR and NOE interactions.

In conclusion, we have also shown the possibility to construct interesting bicyclic intermediates in a 2 step sequence combining the Pd^II^-catalysed cyclisation [36–40] or Pd^0^-allylic substitution [42–43] of alkenols having an allylic OR group and additional halocyclisation.

## Conclusion

In summary, we have developed the syntheses of several unsaturated alcohols. The chiral alkenols **20**–**28**, **34**–**37** and **43** represent useful C5–C12 chain building blocks.

The stereoselective Pd^II^–Cu^II^-catalysed cyclisation [22] and its substrate scope has been investigated. The bicyclisation reaction appears to be applicable only to terminal olefinic substrates, while the reaction of alkenols bearing nonterminal and/or disubstituted olefins did not provide bicyclisation products. Moreover, alkenes having both an allylic OR group and a hydroxylated tether underwent intramolecular Wacker-type cyclisation affording corresponding vinyltetrahydrofurans, which constitute useful intermediates for the synthesis of naturally occurring tetrahydrofuran derivatives.

We have also explored the Pd-cyclisation of unsaturated polyols in the presence of a strong base or a high oxidation state palladium catalyst. The Pd^II^–Pd^IV^-catalysed transformation toward the bicyclisation product proceeded only on the *O*-silyl-protected triol **12**. The Pd^II^-cyclisation of terminal olefinic substrates in the presence of BuLi and LiCl provided selectively 5-*exo*-trig cyclisation products with excellent 2,3-*trans* diastereoselectivity.

Finally, we have also proposed a synthetic access to the dioxaheptane core of natural C_15_ acetogenins and dioxaoctane, a substructure of the macrolide-polyether antibiotic sorangicin A and aurovertins. Thus, Pd-catalysed cyclisation of appropriate alkenols to tetrahydrofurans and subsequent iodo-cyclisation yielded properly substituted 2,5-dioxabicyclo[2.2.1]heptane and 2,6-dioxabicyclo[3.2.1]octane, respectively with defined stereochemistry and excellent diastereoselectivity. The further synthetic studies toward ocellenynes are currently underway.

## Supporting Information

File 1 Mechanisms, general information, experimental procedures and spectroscopic data for all new compounds.

File 2 ^1^H NMR and ^13^C NMR spectra of selected compounds.

File 3 X-ray crystal structure analysis of **53**.
