# Supplementary material for: Palladium-catalysed cyclisation of alkenols: Synthesis of oxaheterocycles as core intermediates of natural compounds
Source: Beilstein J Org Chem. 2014 Sep 3;10:2077–86. doi: 10.3762/bjoc.10.216 (PMC4168889; doi:10.3762/bjoc.10.216)
Supplement: File 1 — Mechanisms, general information, experimental procedures and spectroscopic data for all new compounds. [file Beilstein_J_Org_Chem-10-2077-s001.pdf]

Supporting Information File 1

for

Palladium-catalysed cyclisation of alkenols: Synthesis of oxaheterocycles as core intermediates of natural compounds

Miroslav Palík[1], Jozef Kožíšek[2], Peter Koóš[3] and Tibor Gracza*[1]

Address: [1]Department of Organic Chemistry Slovak University of Technology, Radlinského 9, SK-812 37 Bratislava, Slovakia, [2]Department of Physical Chemistry, Slovak University of Technology, Radlinského 9, SK-812 37 Bratislava, Slovakia and[3]Georganics Ltd., Koreničova 1, SK-811 03 Bratislava, Slovakia

Email: Tibor Gracza - tibor.gracza@stuba.sk

* Corresponding author

Mechanisms, general information, experimental procedures and spectroscopic data for all new compounds

Contents

1. Mechanisms……………………………………………………………………………………..……. S2
2. General Information…………………………………………………………..…….……………..……..S3
3. Experimental procedures and characterisation data………………………..…………………………......S3–S15
4. References………………………………………………………………………….………………..……..S15

1. Mechanisms

Scheme 1: Suggested mechanisms for Pd^{II}-Pd^{0} and Pd^{II}-Pd^{IV} bicyclisation of the triol **12.**

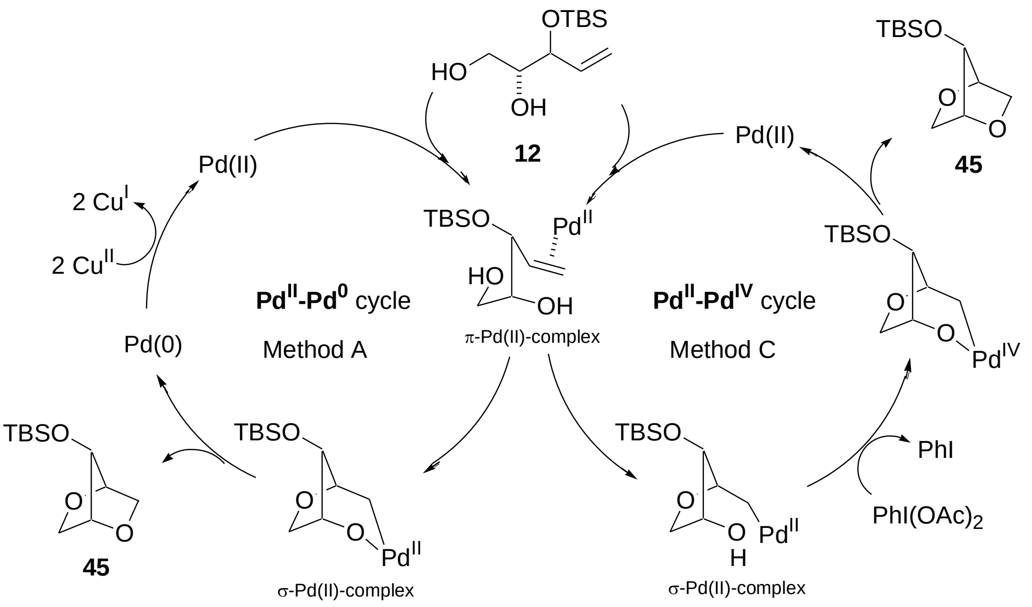

Method A: $PdCl_2$ (0.1 equiv), $CuCl_2$ (3 equiv), NaOAc (3 equiv), AcOH, rt.;

Method C: $Pd(OAc)_2$ (0.1 equiv), $PhI(OAc)_2$ (2 equiv), Me4N+Cl- (1 equiv), NaOAc (1 equiv), AcOH, rt.

Scheme 2: Suggested mechanisms for transformation of diol **33** to bicyclic ether **51** and furan derivative **52**.

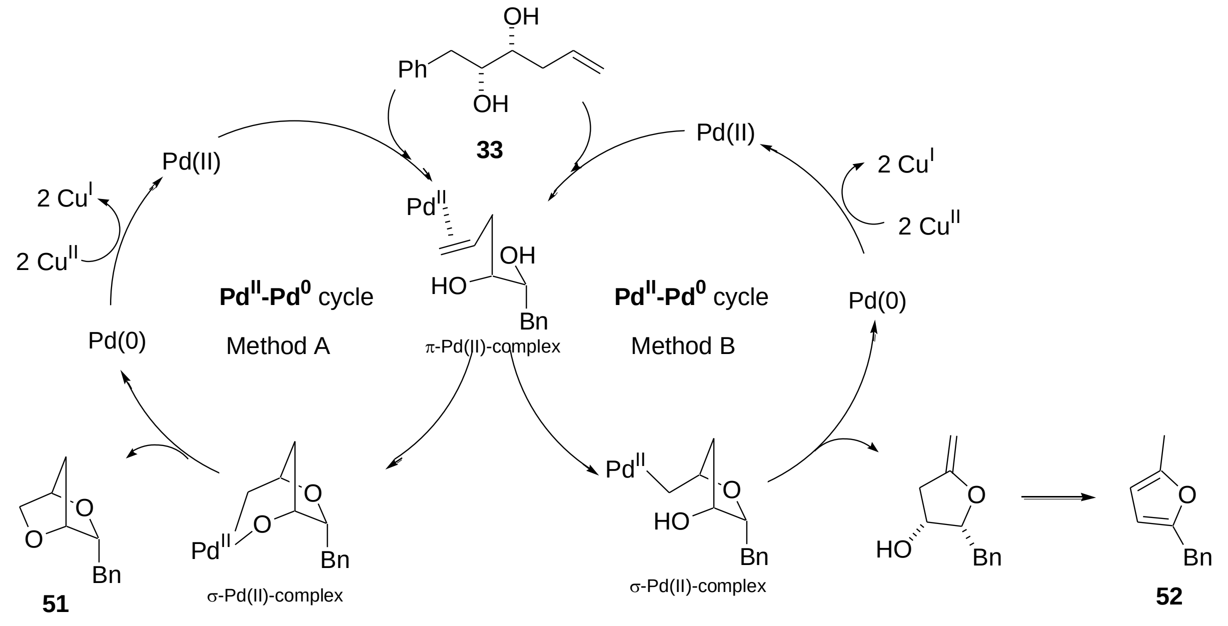

Method A: $PdCl_2$ (0.1 equiv), $CuCl_2$ (3 equiv), NaOAc (3 equiv), AcOH, rt.;

Method B: $PdCl_2(MeCN)_2$ (0.1 equiv), BuLi (2 equiv), $CuCl_2$ (3 equiv), LiCl (3 equiv), THF, rt.;

2. General Information

^{1}H and ^{13}C NMR spectra were recorded on either 300 (75) MHz or 600 (150) MHz Varian spectrometer. Chemical shifts (δ) are quoted in ppm and are referenced to the tetramethylsilane (TMS) as internal standard. High resolution mass spectra (HRMS) were recorded on a Kratos Concept-IS mass spectrometer, and are accurate to ± 0.001. Optical rotations were measured with a JASCO P-2000 polarimeter and are given in units of 10^{-1} deg·cm^{2}·g^{-1}. FTIR spectra were obtained on a Nicolet 5700 spectrometer (Thermo Electron) equipped with a Smart Orbit (diamond crystal ATR) accessory, using the reflectance technique (4000-400 cm^{-1}). Melting points were obtained using a Boecius apparatus and are uncorrected. Commercial reagents were used without further purification. All solvents were distilled before use. Hexanes refer to the fraction boiling at 60-65°C. Flash column liquid chromatography (FLC) was performed on silica gel Kieselgel 60 (40-63 μm, 230-400 mesh) using Buchi Sepacore® preparative MPLC system and analytical thin-layer chromatography (TLC) was performed on aluminum plates pre-coated with either 0.2 mm (DC-Alufolien, Merck) or 0.25 mm silica gel 60 F_{254} (ALUGRAM® SIL G/UV_{254}, Macherey-Nagel). The compounds were visualised by UV fluorescence and by dipping the plates in an aqueous H_2SO_4 solution of cerium sulphate/ammonium molybdate followed by charring with a heat gun.

Syntheses of substrates 12, 15, 16, 18-20, 22-30, 33-35, 37 and *rac*-**42**

12: According to literature[1] to a solution of diastereomeric mixture of (2*S*,3*R*)- and (2*S*,3*S*)-1,2-*O*-isopropylidenepent-4-ene-1,2,3-triol[2] (1000 mg, 6.3 mmol) in dry DMF (15 mL) was added imidazole (2.583 g, 37.9 mmol, 6 equiv.) and TBDPSCl (5.214 g, 18.9 mmol, 3 equiv.) at room temp. After stirring the mixture at room temp. overnight, the reaction mixture was diluted with ethyl acetate (200 mL) and washed with water (2 x 70 mL). Organic phase was dried over Na_2SO_4 and concentrated. The crude product was dissolved in aqueous acetic acid (50%, 50 mL) and heated at 50 °C overnight. The solvents were removed *in vacuo*, and the residue purified by flash chromatography (EtOAc/hexanes 1:1). Yield of silyl-protected triol **12** was 875 mg (42% over two steps), colourless oil [R_f = 0.15, EtOAc/hexanes 1:1, mixture of (2*S*,3*R*)- and (2*S*,3*S*)-**12** in ratio 60:40].

IR (film, cm^{-1}): ν 3367 (s), 3070 (s), 2856 (s), 1472 (s), 1427 (s), 1390 (s), 1106 (s), 1073 (s), 821 (s), 740 (s), 700 (s), 613 (s), 507 (s).

^{1}H NMR (300 MHz, $CDCl_3$) mixture of isomers: δ=7.73–7.60 (m, 1H), 7.48–7.32 (m, 1H), 5.87–5.72 (m, 1H), 5.08–4.91 (m, 1H), 4.25–4.15 (m, 1H), 3.65–3.48 (m, 1H), 2.26-2.22 (bs, 2H), 1.08 (s, 2H).

^{13}C NMR (75 MHz, $CDCl_3$) mixture of isomers: δ= 136.6, 136.3, 136.0, 135.8, 133.2, 133.1, 129.9, 129.8, 127.7, 127.5, 118.0, 117.9, 76.7, 75.9, 74.7, 74.5, 63.0, 62.85, 27.0, 19.3.

15: A solution of DMSO (14.81 g, 189.6 mmol, 2.25 equiv.) in CH_2Cl_2 (130 mL) was slowly added to oxalyl chloride (15.939 g, 126.6 mmol, 1.5 equiv.) in CH_2Cl_2 (130 mL) at -78 °C and stirred for 30 min under Ar. A solution of 2-*O*-benzyl-3,4-*O*-isopropylidene-L-threitol **14** (21.269 g, 84.3 mmol) in CH_2Cl_2 (130 mL) was added dropwise at -78 ^{o}C. After 30 min stirring at -78 ^{o}C Et_3N (47.1 mL, 337.2 mmol, 4 equiv.) was added. After 1 h at -78 °C, the mixture was allowed to warm to room temperature over 1 h. The mixture was concentrated *in vacuo*, diluted with ether and filtered through a silica gel pad. The filtrate and washings were combined and concentrated under reduced pressure. The crude product (30.9 g, R_f = 0.57, EtOAc/hexanes 1:1) was used without further purification in the next step.

General procedure for Wittig olefination: A solution of butyl lithium (2 M in hexanes, 10.5 mL, 21 mmol, 1.9 equiv.) was added to a dispersion of phosphonium salt (22 mmol, 2 equiv.) in tetrahydrofuran (100 mL) at 0 ^{o}C over 10 min. After 30 min. stirring at 0 ^{o}C a solution of the crude threose **15** (2.75 g, 10.99 mmol) in THF (30 mL) was added and the solution was left to stir at room temperature overnight (12 h), and then quenched with saturated solution of NH_4Cl (100 mL). Ether (100 mL) was added and the separated aqueous phase extracted with Et_2O (3 × 80 mL). Organic phases were washed with brine (70 mL), dried over Na_2SO_4 and concentrated. Residue was purified by flash chromatography (9% ethyl acetate in hexanes).

3. Experimental procedures and characterisation data

E-**16**: Prepared by general procedure for Wittig olefination using benzyltriphenylphosphonium bromide. Yield 413 mg (17% over two steps from **14**); $[\alpha]_D^{20}$ = + 59.2 (*c* = 0.148, $CHCl_3$); HRMS: *m/z* (ESI) calculated for $C_{21}H_{24}O_3$ $[M+Na]^+$ 347.1623, found 347.1567.

IR (film, cm^{-1}): ν 3026 (s), 2983 (s), 2880 (s), 1493 (s), 1452 (s), 1380 (s), 1370 (s), 1213 (s), 1071 (s), 1040 (s), 1030 (s), 847 (s), 810 (s).

^{1}H NMR (300 MHz, $CDCl_3$): *δ*=7.43–7.23 (m, 10H, Ph), 6.62 (d, *J* = 16.0 Hz, 1H, H-3'), 6.08 (dd, *J* = 16.0, 8.2 Hz, 1H, H-2'), 4.73 (d, *J* = 12.4 Hz, 1H, O-CH_2-Ph), 4.53 (d, *J* = 12.4 Hz, 1H, O-CH_2-Ph), 4.30 (q, *J* = 6.5 Hz, 1H, H-4), 4.05–3.95 (m, 2H, H-5_a, H-1'), 3.83 (dd, *J* = 8.6, 6.2 Hz, 1H, H-5_b), 1.40 (s, 3H, CH_3),1.37 (s, 3H, CH_3).

^{13}C NMR (75 MHz, $CDCl_3$): δ = 138.1 (s, i-Ph); 136.2 (s, i-Ph); 135.3 (d, C-3'); 128.7, 128.3, 128.2, 128.1, 127.9, 127.4 (6xd, Ph); 127.3 (d, C-2'); 109.7 (s, C-2); 77.9 (d, C-1'); 73.8 (d, C-4); 70.1 (t, O-CH_2-Ph); 65.4 (t, C-5); 26.5 (q, CH_3); 25.4 (q, CH_3).

Z-**16**: Yield 875 mg (36% from **14**); $[\alpha]_D^{20}$ = + 53.4 (*c* = 0.079, $CHCl_3$); HRMS: *m/z* (ESI) calculated for $C_{21}H_{24}O_3$ $[M+Na]^+$ 347.1623, found 347.1567.

IR (film, cm^{-1}): ν 3026 (s), 2983 (s), 2881 (s), 1495 (s), 1454 (s), 1380 (s), 1369 (s), 1213 (s), 1071 (s), 1043 (s), 1028 (s), 846 (s), 805 (s).

^{1}H NMR (300 MHz, $CDCl_3$): *δ*=7.37 – 7.08 (m, 10H, Ph), 6.86 (dd, *J* = 11.8, 1H, H-3'), 5.63 (dd, *J* = 11.8, 10.1 Hz, 1H, H-2'), 4.58 (d, *J* = 12.0 Hz, 1H, O-CH_2-Ph), 4.47 (ddd, *J* = 10.1, 5.8, 0.9 Hz, 1H, H-1'), 4.31 (ddd *J* = 6.6, 5.7, 5.5 Hz, 1H, H-4), 4.27 (d, *J* = 12.0 Hz, 1H, O-CH_2-Ph),4.01 (dd, *J* = 8.7, 6.6 Hz, 1H, H-5_a), 3.94 (dd, *J* = 8.8, 5.5 Hz, 1H, H-5_b), 1.38 (s, 3H, CH_3), 1.36 (s, 3H, CH_3).

^{13}C NMR (75 MHz, $CDCl_3$): δ = 138.1 (s, i-Ph); 136.3 (s, i-Ph); 135.2 (d, C-3'); 128.7, 128.2, 128.1, 128.0, 127.9, 127.4 (6xd, Ph); 127.3 (d, C-2'); 109.7 (s, C-2); 77.9 (d, C-1'); 73.8 (d, C-4); 70.0 (t, O-CH_2-Ph); 65.4 (t, C-5); 26.4 (q, CH_3); 25.3 (q, CH_3).

Z-**18**: Prepared by general procedure for Wittig olefination using ethyltriphenylphosphonium bromide. Yield 963 mg (49% from **14**); $[\alpha]_D^{20}$ = + 10.2 (*c* = 0.075, $CHCl_3$); HRMS: *m/z* (ESI) calculated for $C_{16}H_{22}O_3$ $[M+Na]^+$ 285.1467, found 285.1505.

IR (film, cm^{-1}): ν 2980 (s), 1705 (s), 1657 (s), 1496 (s), 1369 (s), 1261 (s), 1210 (s), 1175 (s), 1066 (s), 985 (s), 777 (s), 847 (s), 510 (s).

^{1}H NMR (300 MHz, $CDCl_3$): *δ*=7.43–7.22 (m, 5H, Ph), 5.84 (dq, *J* = 11.1, 7.0 Hz, 1H, H-3'), 5.37–5.25 (m, 1H, H-2'), 4.67 (d, *J* = 12.4 Hz, 1H, O-CH_2-Ph), 4.45 (d, *J* = 12.4 Hz, 1H, O-CH_2-Ph), 4.25–4.15 (m, 2H, H-4, H-1'), 3.97–3.91 (m, 1H, H-5_a), 3.68–3.62 (m, 1H, H-5_b), 1.62 (dd, J = 7.0, 1.8 Hz, 3H, H-4'), 1.41 (s, 3H, CH_3), 1.36 (s, 3H, CH_3).

^{13}C NMR (75 MHz, $CDCl_3$): *δ*=138.6 (s, i-Ph), 131.0, 128.3, 127.9 (3xd, Ph), 127.5 (d, C-3'), 126.8 (d, C-2'), 109.8 (s, C-2), 78.2 (d, C-4), 74.6 (d, C-1'), 69.9 (t, O-CH_2-Ph), 65.9 (t, C-5), 26.7 (q, CH_3), 25.5 (q, CH_3), 14.0 (q, C-4').

Z-**19**: Prepared by general procedure for Wittig olefination using 2-phenylethyltriphenylphosphonium bromide. Yield 1.344 g (53% from **14**); $[\alpha]_D^{20}$ = + 28.1 (*c* = 0.1, $CHCl_3$); HRMS: *m/z* (ESI) calculated for $C_{22}H_{26}O_3$ $[M+Na]^+$ 361.1780, found 361.1745.

IR (film, cm^{-1}): ν 3062 (s), 3028 (s), 2881 (s), 1712 (s), 1604 (s), 1495 (s), 1453 (s), 1067 (s), 1028 (s), 850 (s), 737 (s), 697 (s), 511 (s).

^{1}H NMR (300 MHz, $CDCl_3$): *δ*=7.38–7.11 (m, 10H, Ph), 5.94 (dddd, *J* = 11.1, 8.0, 7.1, 0.9 Hz, 1H, H-3'), 5.44 (ddd, *J* = 11.1, 9.4, 1.7 Hz, 1H, H-2'), 4.71 (d, *J* = 12.4 Hz, 1H, O-CH_2-Ph), 4.49 (d, *J* = 12.4 Hz, 1H, O-CH_2-Ph), 4.33 (ddd, *J* = 9.3, 6.7, 0.9 Hz, 1H, H-1'), 4.26 (ddd, *J* = 6.5, 6.4, 6.2 Hz, 1H, H-4), 3.97 (dd, *J* = 8.6, 6.5 Hz, 1H,

H-5_a), 3.72 (dd, *J* = 8.6, 6.2 Hz, 1H, H-5_b), 3.43 (ddd, *J* = 16.0, 8.0, 2.1 Hz, 1H, H-4'a), 3.32 (ddd, *J* = 15.9, 7.2, 1.8 Hz, 1H, H-4'$_b$), 1.42 (s, 3H, C$\underline{H}_3$), 1.38 (s, 3H, C$\underline{H}_3$).

^{13}C NMR (75 MHz, $CDCl_3$): δ=139.8 (s, i-Ph), 138.3 (s, i-Ph), 134.9 (d, C-3'), 134.8 (d, C-2'), 128.5, 128.3, 127.7, 127.5, 126.6, 126.2 (6xd, Ph), 109.7 (s, C-2), 78.0 (d, C-4), 74.7 (d, C-1'), 70.0 (t, O-$\underline{C}H_2$-Ph), 65.7 (t, C-5), 34.2 (q, C-4'), 26.5 (q, $\underline{C}H_3$), 25.4 (q, $\underline{C}H_3$).

General procedure for acetonide hydrolysis: A solution of acetonide (2 mmol) was dissolved in 60% AcOH (30 mL) and heated to 60 ^{o}C for 3 h. The solvents were removed *in vacuo* and the crude product was purified by FLC (silica gel, gradient from 0 to 50% EtOAc in hexanes).

E-**20**: Yield 405 mg (77%); $[\alpha]_D^{20}$ = + 88.2 (*c* = 0.378, $CHCl_3$); HRMS: *m/z* (ESI) calculated for $C_{18}H_{20}O_3$ $[M+Na]^+$ 307.1310, found 307.1312.

IR (film, cm^{-1}): ν 3375 (bs), 3026 (s), 2924 (s), 2868 (s), 1494 (s), 1452 (s), 1043 (s), 1027 (s), 970 (s), 875 (s), 746 (s), 693 (s), 544 (s).

^{1}H NMR (300 MHz,$CDCl_3$): δ=7.47–7.14 (m, 10H, Ph), 6.69 (d, *J* = 16.1 Hz, 1H, H-5), 6.15 (dd, *J* = 16.0, 8.4 Hz, 1H, H-4), 4.70 (d, *J* = 11.6 Hz, 1H, O-C$\underline{H}_2$-Ph), 4.42 (d, *J* = 11.6 Hz, 1H, O-C$\underline{H}_2$-Ph), 4.05 (dd, *J* = 8.3, 6.9 Hz, 1H, H-3), 3.79–3.70 (m, 2H H-1), 3.68–3.58 (m, 1H, H-2), 2.96 (bs, 1H, O$\underline{H}$), 2.17 (bs, 1H, O$\underline{H}$).

^{13}C NMR (75 MHz, $CDCl_3$): δ=137.7 (s, i-Ph), 135.9 (s, i-Ph), 135.5 (d, C-5), 128.6, 128.5, 128.2, 128.0, 127.9, 126.6 (6xd, Ph), 125.5 (d, C-4), 81.0 (d, C-3), 74.0 (d, C-2), 70.5 (t, O-$\underline{C}H_2$-Ph), 63.0 (t, C-1).

Z-**20**: Yield 422 mg (74%); $[\alpha]_D^{20}$ = + 67.4 (*c* = 0.370, $CHCl_3$); HRMS: *m/z* (ESI) calculated for $C_{18}H_{20}O_3$ $[M+Na]^+$ 307.1310, found 307.1312.

IR (film, cm^{-1}): ν 3381 (bs), 3026 (s), 2924 (s), 2868 (s), 1494 (s), 1453 (s), 1390 (s), 1261 (s), 1205 (s), 1086 (s), 1041 (s), 1027 (s), 697 (s).

^{1}H NMR (300 MHz, $CDCl_3$): δ=7.39–7.21 (m, 8H, Ph), 7.14–7.04 (m, 2H, Ph), 6.92 (d, *J* = 11.9 Hz, 1H, H-5), 5.64 (dd, *J* = 11.9, 10.1 Hz, 1H, H-4), 4.55 (d, *J* = 11.5 Hz, 1H, O-C$\underline{H}_2$-Ph), 4.46 (ddd, *J* = 10.1, 6.6, 1.0 Hz, 1H, H-3), 4.14 (d, *J* = 11.5 Hz, 1H, O-C$\underline{H}_2$-Ph), 3.76 (d, *J* = 6.4 Hz, 2H, H-1), 3.70 – 3.58 (m, 1H, H-2), 2.86 (d, *J* = 3.5 Hz, 1H, O$\underline{H}$), 2.14 (bs, 1H, O$\underline{H}$).

^{13}C NMR (75 MHz, $CDCl_3$): δ=137.6 (s, i-Ph), 136.2 (s, i-Ph), 135.8 (d, C-5), 128.7, 128.4, 128.3, 128.2, 128.0, 127.8 (6xd, Ph), 127.5 (d, C-4), 74.6 (d, C-3), 74.0 (d, C-2), 70.1 (t, O-$\underline{C}H_2$-Ph), 63.0 (t, C-1).

22: Yield 386 mg (87%); $[\alpha]_D^{20}$ = + 22.5 (*c* = 0.02, $CHCl_3$); HRMS: *m/z* (ESI) calculated for $C_{13}H_{18}O_3$ $[M+Na]^+$ 245.1154, found 245.1051.

IR (film, cm^{-1}): ν 3391 (bs), 3309 (s), 2915 (s), 2850 (s), 1610 (s), 1440 (s), 1090 (s), 1041 (s), 965 (s), 810 (s), 775 (s), 699 (s), 495 (s).

^{1}H NMR (300 MHz, $CDCl_3$): δ=7.41–7.27 (m, 5H, Ph), 5.90 (dq, *J* = 11.1, 7.0 Hz, 1H, H-5), 5.35 (ddd, *J* = 9.5, 6.5, 1.8 Hz, 1H, H-4), 4.63 (d, *J* = 11.5 Hz, 1H, O-C$\underline{H}_2$-Ph), 4.35 (d, *J* = 11.5 Hz 1H, O-C$\underline{H}_2$-Ph), 4.26 (ddd, *J* = 9.5, 7.6, 0.9 Hz, 1H, H-3), 3.67 (m, 2H, H-1), 3.56–3.47 (m, 1H, H-2), 2.99 (d, *J* = 2.6 Hz, 1H, O$\underline{H}$), 2.20 (t, *J* = 6.3 Hz, 1H, O$\underline{H}$), 1.70 (dd, *J* = 7.0, 1.8 Hz, 3H, H-6).

^{13}C NMR (75 MHz, $CDCl_3$): δ=139.3 (s, i-Ph), 132.9 (d, C-5), 129.8, 129.3, 129.23 (3xd, Ph), 128.4 (d, C-4), 76.2 (d, C-3), 75.3 (d, C-2), 71.5 (t, O-$\underline{C}H_2$-Ph), 64.2 (t, C-1), 15.0 (q, C-6).

23: Yield 488 mg (82%); $[\alpha]_D^{20}$ = + 23.2 (*c* = 0. 315, $CHCl_3$); HRMS: *m/z* (ESI) calculated for $C_{19}H_{22}O_3$ $[M+Na]^+$ 321.1467, found 321.1463.

IR (film, cm^{-1}): ν 3383 (bs), 3060 (s), 3026 (s), 3873 (s), 1495 (s), 1453 (s), 1060 (s), 1028 (s), 876 (s), 735 (s), 697 (s), 461 (s), 472 (s).

^{1}H NMR (300 MHz, $CDCl_3$): δ=7.41–7.18 (m, 10H, Ph), 6.03 (dddd, J = 11.1, 8.8, 8.0, 0.9 Hz, 1H, H-5), 5.48 (ddd, J = 11.2, 9.6, 1.7 Hz, 1H, H-4), 4.69 (d, J = 11.5 Hz, 1H, O-C$\underline{H}_2$-Ph), 4.44–4.39 (m, 1H, H-3), 4.39 (d, J = 11.3 Hz, 1H, O-C$\underline{H}_2$-Ph), 3.82–3.65 (m, 2H, H-1), 3.64–3.55 (m, J = 11.2 Hz, 1H, H-4), 3.55–3.40 (m, 2H, H-6), 3.00 (bs, 1H, O$\underline{H}$), 2.18 (bs, 1H, O$\underline{H}$).

^{13}C NMR (75 MHz, $CDCl_3$): δ=139.9 (s, i-Ph), 137.8 (s, i-Ph), 135.5 (d, C-5), 128.6, 128.5, 128.3, 127.9, 127.8, 127.0 (6xd, Ph), 126.2 (d, C-4), 75.2 (d, C-3), 73.9 (d, C-2), 70.3 (t, O-$\underline{C}H_2$-Ph); 62.8 (t, C-1), 34.0 (t, C-6).

24: Yield 819 mg (64% from *E*-**17**); $[\alpha]_D^{20}$= + 47.7 (c = 0.71, $CHCl_3$); HRMS: *m/z* (ESI) calculated for $C_{13}H_{18}O_4$ $[M+Na]^+$ 261.1103, found 261.1146.

IR (film, cm^{-1}): ν 3400 (bs), 3030 (s), 2917 (s), 2854 (s), 1722 (s), 1453 (s), 1251 (s), 1212 (s), 1084 (s), 1071 (s), 1026 (s), 737 (s), 698 (s).

^{1}H NMR (300 MHz, $CDCl_3$): δ=7.39–7.26 (m, 5H, Ph), 5.94 (ddd, J = 15.6, 5.2, 4.8 Hz, 1H, H-5), 5.66 (ddd, J = 15.6, 8.1, 1.6 Hz, 1H, H-4), 4.67–4.58 (m, 2H, H-3, O-C$\underline{H}_2$-Ph), 4.35 (d, J = 11.6 Hz, 1H, O-C$\underline{H}_2$-Ph), 4.17 (dd, J = 5.0, 1.6 Hz, 1H, H-2), 3.89 (dd, J = 7.8, 6.3 Hz, 1H, H-1_a), 3.74 – 3.51 (m, 3H, H-1_b, H-6), 3.20 (bs, 1H, O$\underline{H}$), 2.68 (bs, 1H, O$\underline{H}$), 2.37 (bs, 1H, O$\underline{H}$).

^{13}C NMR (75 MHz, $CDCl_3$): δ=137.8 (s, i-Ph), 135.2 (d, C-5), 128.5, 127.9, 127.9 (3xd, Ph), 127.2 (d, C-4), 80.4 (d, C-3), 73.9 (d, C-3), 70.5 (t, O-$\underline{C}H_2$-Ph), 63.1 (t, C-6), 62.5 (t, C-1).

25: To a solution of 1,2-*O*-isopropylidene-3-*O*-benzylhex-4-ene-6-ol[3] (1.00 g, 3.59 mmol, prepared by DIBAL reduction of *E*-**17**) in dichloromethane (50 mL) *tert*-butyldiphenylsilyl chloride (1.15 g, 4.66 mmol, 1.3 equiv.) and imidazole (366 mg, 5.39 mmol, 1.5 equiv.) were added at room temperature. After stirring the mixture overnight at rt, dichloromethane was removed *in vacuo*. The residue was extracted between water (50 mL) and diethyl ether (3 x 50 mL). The organic layers were washed with brine (50 mL), dried over Na_2SO_4 and concentrated. The crude product was dissolved in $CHCl_3$ (50 mL) and $FeCl_3.6H_2O$ (1.778 g, 6.579 mmol, 2 equiv.) was added at room temperature in one portion and kept for 1 h with stirring. The reaction the mixture was quenched with sat. aq $NaHCO_3$ (30 mL) and extracted with $CHCl_3$ (3 x 40 mL). Removal of the solvent *in vacuo* and flash chromatography purification (silica gel, gradient from 0 to 50% EtOAc in hexanes) gave **25** (950 mg, 56% over two steps).

HRMS: *m/z* (ESI) calculated for $C_{29}H_{36}O_4Si$ $[M+Na]^+$ 499.2281, found 499.2198; $[\alpha]_D^{20}$ = + 28.9 (c = 0.3, $CHCl_3$).

IR (film, cm^{-1}): ν 3392 (bs), 3068 (s), 2929 (s), 2856 (s), 1705 (s), 1471 (s), 1427 (s), 1263 (s), 1106 (s), 1068 (s), 1028 (s), 739 (s), 700 (s).

^{1}H NMR (300 MHz, $CDCl_3$): δ=7.73–7.61 (m, 4H, Ph), 7.47–7.26 (m, 11H, Ph), 5.89 (ddd, J = 15.5, 4.1, 0.6 Hz, 1H, H-5), 5.71 (ddd, J = 15.5, 8.2, 1.6 Hz, 1H, H-4), 4.62 (d, J = 11.6 Hz, 1H, O-C$\underline{H}_2$-Ph), 4.32 (d, J = 11.6 Hz, 1H, O-C$\underline{H}_2$-Ph), 4.29–4.26 (m, 2H, H-6), 3.91–3.83 (m, 1H, H-3), 3.74–3.65 (m, 1H, H-2), 3.64–3.58 (m, 1H, H-1_a), 3.57–3.46 (m, 1H, H-1_b), 2.85 (d, J = 2.4 Hz, 1H, O$\underline{H}$), 2.08 (bs, 1H, O$\underline{H}$), 1.06 (s, 9H, $(C\underline{H}_3)_3CSi$).

^{13}C NMR (75 MHz, $CDCl_3$): δ=137.8 (s, i-Ph), 135.6 (s, i-Ph), 135.4 (d, C-5), 133.5, 129.7, 128.5, 127.9, 127.8, 127.7 (6xd, Ph), 125.8 (d, C-4), 80.4 (d, C-3), 73.8 (d, C-2), 70.3 (t, O-$\underline{C}H_2$-Ph), 63.5 (t, C-6), 63.0 (t, C-1), 26.8 (q, $(\underline{C}H_3)_3CSi$), 19.2 (s, $(CH_3)_3\underline{C}Si$).

26: To a solution of 1,2-*O*-isopropylidene-3-*O*-benzylhex-4-ene-6-ol[3] (1.10 g, 3.95 mmol, prepared by DIBAL reduction of *E*-**17**) in tetrahydrofuran (25 mL) sodium hydride (60% in mineral oil, 475 mg, 11.86 mmol, 3 equiv.) was added at room temperature. After 1 h stirring methyl iodide (3.364 g, 23.7 mmol, 6 equiv.) was added and stirred overnight at rt. The mixture was quenched with sat. aq NH_4Cl (30 mL) and extracted with diethyl ether (3 x 50 mL). Organic phase was dried over Na_2SO_4 and concentrated to give oil which was dissolved in 60% AcOH (50 mL) and heated at 60 °C for 3 h. The solvents were removed *in vacuo* and the residue was purified by flash chromatography (silica gel, gradient from 0 to 50% ethyl acetate in hexanes); yield 835 mg (75% over two steps).

HRMS: *m/z* (ESI) calculated for $C_{14}H_{20}O_4$ $[M+Na]^+$ 275.1259, found 275.1212; $[\alpha]_D^{20}$ = + 46.8 (c = 0.22, $CHCl_3$).

IR (film, cm^{-1}): ν 3385 (bs), 3030 (s), 2927 (s), 2873 (s), 1721 (s), 1496 (s), 1454 (s), 1382 (s), 1089 (s), 1068 (s), 1027 (s), 978 (s), 910 (s).

^{1}H NMR (300 MHz, $CDCl_3$): δ=7.39–7.27 (m, 5H, Ph), 5.90 (ddd, J = 15.4, 5.4, 0.5 Hz, 1H, H-5), 5.68 (ddd, J = 15.7, 8.1, 1.5 Hz, 1H, H-4), 4.66 (d, J = 11.6 Hz, 1H, O-C$\underline{H}_2$-Ph), 4.35 (d, J = 11.6 Hz, 1H, O-C$\underline{H}_2$-Ph), 3.99 (dd, J = 5.4, 1.5 Hz, 1H, H-8_a), 3.90 (dd, J = 8.2, 6.5 Hz, 1H, H-3), 3.74–3.52 (m, 4H, H-1, H-2, H-8_b), 3.37 (s, 3H, C$\underline{H}_3$-O), 3.01 (bs, 1H, O$\underline{H}$), 2.36 (bs, 1H, O$\underline{H}$).

^{13}C NMR (75 MHz, $CDCl_3$): δ=137.7 (s, i-Ph), 132.7 (d, C-5), 128.7, 128.5, 127.9 (3xd, Ph), 127.8 (d, C-4), 80.3 (d, C-3), 73.8 (d, C-2), 72.1 (t, C-6), 70.4 (t, O-$\underline{C}H_2$-Ph), 63.0 (t, C-1), 58.1 (q, $\underline{C}H_3$-O).

27: To a solution of *Z*-**17**[4] (810 mg, 2.52 mmol) in diethyl ether (40 mL) methyl lithium (1M solution in diethyl ether, 5 mL, 5.04 mmol, 2 equiv.) was added at -78 °C during 15 min and stirring was continued at this temperature for 1 h. The mixture was quenched with sat. aq NH_4Cl (20 mL) and extracted with diethyl ether (3 x 30 mL). Organic layers were washed with brine (30 mL), dried over Na_2SO_4 and concentrated. The crude product was purified by flash chromatography (silica gel, gradient from 0 to 35% ethyl acetate in hexanes); yield 437 mg (57%).

HRMS: *m/z* (ESI) calculated for $C_{18}H_{26}O_4$ $[M+Na]^+$ 329.1729, found 329.1757; $[\alpha]_D^{20}$ = + 16.6 (c = 0.135, $CHCl_3$).

IR (film, cm^{-1}): ν 3427 (s), 2976 (s), 2932 (s), 2871 (s), 1731 (s), 1454 (s), 1370 (s), 1211 (s), 1061 (s), 1028 (s), 975 (s), 850 (s), 697 (s).

^{1}H NMR (300 MHz, $CDCl_3$): δ=7.37–7.26 (m, 5H, Ph), 5.82 (d, J = 12.0 Hz, 1H, H-3'), 5.27 (dd, J = 12.0, 8.9 Hz, 1H, H-2'), 5.15 (dd, J = 9.3, 5.3 Hz, 1H, H-1'), 4.67 (d, J = 11.9 Hz, 1H, O-C$\underline{H}_2$-Ph), 4.49 (d, J = 11.9 Hz, 1H, O-C$\underline{H}_2$-Ph), 4.34 (dt, J = 6.7, 5.1 Hz, 1H, H-4), 4.02 (dd, J = 8.6, 6.6 Hz, 1H, H-5_a), 3.93 (dd, J = 8.7, 6.8 Hz, 1H, H-5_b), 3.27 (s, 1H, O$\underline{H}$), 1.43 (s, 3H, C$\underline{H}_3$), 1.35 (s, 3H, C$\underline{H}_3$), 1.31 (s, 3H, C$\underline{H}_3$), 1.30 (s, 3H, C$\underline{H}_3$).

^{13}C NMR (75 MHz, $CDCl_3$): δ=143.3 (s, i-Ph), 138.3 (d, C-3'), 128.3, 127.6, 127.6 (3xd, Ph), 124.5 (d, C-2'), 109.5 (s, C-2), 76.3 (d, C-4), 74.2 (d, C-1'), 71.6 (s, C-6), 71.12 (t, O-$\underline{C}H_2$-Ph), 65.2 (t, C-5), 32.4 (q, $\underline{C}H_3$), 30.0 (q, $\underline{C}H_3$), 26.3 (q, $\underline{C}H_3$), 25.2 (q, $\underline{C}H_3$).

28: According to general procedure for acetonide hydrolysis **27** (567 mg, 1.77 mmol) in 60% AcOH (10 mL) and heated to 60 °C for 3 h. Purification by FLC (silica gel, gradient from 0 to 40% EtOAc in hexanes) provided **28** (398 mg, 84%).

HRMS: *m/z* (ESI) calculated for $C_{15}H_{22}O_4$ $[M+Na]^+$ 289.1416, found 289.1369; $[\alpha]_D^{20}$ = + 49.1 (c = 0.11, $CHCl_3$).

IR (film, cm^{-1}): ν 3393 (bs), 3069(s), 2929 (s), 2856 (s), 1705 (s), 1469 (s), 1424 (s), 1260 (s), 1106 (s), 1068 (s), 1025 (s), 739 (s), 700 (s).

^{1}H NMR (300 MHz, $CDCl_3$): δ=7.40 – 7.27 (m, 5H, Ph), 5.83 – 5.70 (m, 1H, H-5), 5.27 – 5.16 (m, 1H, H-4), 4.67 (d, J = 11.3 Hz, 1H, O-C$\underline{H}_2$-Ph), 4.45 (d, J = 11.4 Hz, 1H, O-C$\underline{H}_2$-Ph), 3.83 (dd, J = 12.1, 2.9 Hz, 1H, H-1_a), 3.70 (dd, J = 12.0, 2.5 Hz, 1H, H-1_b), 3.64 – 3.57 (m, 2H, H-2, H-3), 3.03 (bs, 1H, O$\underline{H}$), 1.38 (s, 3H, C$\underline{H}_3$), 1.35 (s, 3H, C$\underline{H}_3$).

^{13}C NMR (75 MHz, $CDCl_3$): δ=143.1 (s, i-Ph), 138.3 (d, C-5), 128.6, 128.1, 127.9 (3xd, Ph), 124.7 (d, C-4), 75.9 (d, C-3), 74.2 (d, C-2), 72.8 (s, C-6), 71.2 (t, O-$\underline{C}H_2$-Ph), 62.7 (t, C-1), 31.9 (q, $\underline{C}H_3$), 30.4 (q, $\underline{C}H_3$).

29: To a solution of methyl magnesium chloride (3 M in diethyl ether, 22 mL, 63.97 mmol, 3 equiv.) the crude aldehyde **15** (4 g, 15.99 mmol) in diethyl ether (50 mL) was added slowly at 0 °C and the mixture left at room temperature for 1 h. Treatment with sat. aq NH_4Cl (10 mL) followed; the organic phase was separated and the water phase was extracted with diethyl ether (3 x 50 mL). The combined organic layers were washed with brine (50 mL), dried (Na_2SO_4) and concentrated *in vacuo* to leave a light brown oil as a mixture of two diastereomers which was dissolved in dichloromethane (50 mL), cooled to 0 °C and freshly prepared solution of Dess-Martin periodinane (6.8 g, 16.00 mmol, 1 equiv.) in dichloromethane (200 mL) was dropped. After 1 h stirring at room temperature, the mixture was washed with saturated $NaHCO_3$ (2 x 200 mL), brine (100 mL), dried over Na_2SO_4 and concentrated *in vacuo*. The crude product was purified by flash chromatography (silica gel, gradient from 0 to 30% ethyl acetate in hexanes); yield 437 mg (56% over 3 steps from **14**). All spectroscopic data were in good with agreement with those reported in the lit.[5]

30: Following the general procedure for Wittig olefination, from **29** (3.20 g, 12.12 mmol), methyl phosphonium bromide (8.66 g, 24.24 mmol, 2 equiv.) and BuLi (2.5 M in hexanes, 8.7 mL, 21.28 mmol, 1.8 equiv.) in tetrahydrofuran (250 mL), after filtration trough a pad of silica gel (100 g, 20% ethyl acetate in hexanes) and concentration *in vacuo* the 1,2-*O*-isopropylidene protected alkene was obtained. The hydrolysis of the crude intermediate with 60% AcOH (70 mL) at 60 °C over 3 h provided **30** (932 mg, 73%) as a colourless oil after chromatography purification (silica gel, gradient from 0 to 50% ethyl acetate in hexanes).

HRMS: *m/z* (ESI) calculated for $C_{13}H_{18}O_3$ $[M+Na]^+$ 245.1154, found 245.1131; $[\alpha]_D^{20}$ = + 69.1 (*c* = 0.235, $CHCl_3$).

IR (film, cm^{-1}): ν 3380 (bs), 3025 (s), 2865 (s), 2490 (s), 1730 (s), 1040 (s), 1030 (s), 1010 (s), 870 (s), 805 (s), 777 (s), 750 (s), 697 (s).

^{1}H NMR (300 MHz,$CDCl_3$): δ = 7.40–7.27 (m, 5H, Ph), 5.13 („p", *J* = 1.5 Hz, 1H, H-5_a), 5.08 (ddt, *J* = 1.8, 0.9, 0.9 Hz, 1H, H-5_b), 4.56 (d, *J* = 11.4 Hz, 1H, O-C$\underline{H_2}$-Ph), 4.28 (d, *J* = 11.5 Hz, 1H, O-C$\underline{H_2}$-Ph), 3.80 (d, *J* = 7.9 Hz, 1H, H-3), 3.78–3.72 (m, 1H, H-2), 3.68–3.59 (m, 1H, H-1_a), 3.55–3.46 (m, 1H, H-1_b), 2.89 (bs, 1H, O$\underline{H}$), 2.16 (bs, 1H, O$\underline{H}$), 1.75 (dd, *J* = 1.5, 0.9 Hz, 3H, C$\underline{H_3}$).

^{13}C NMR (75 MHz, $CDCl_3$): δ=141.4 (s, i-Ph), 137.8 (t, C-5), 128.6, 128.1, 128.0 (3xd, Ph), 117.0 (s, C-4), 84.0 (d, C-3), 72.2 (d, C-2), 70.4 (t, O-$\underline{C}H_2$-Ph), 63.1 (t, C-1), 17.5 (q, $\underline{C}H_3$).

General procedure for preparation of diols 33-35: To a solution of aldehyde **31** (402 mg, 3 mmol, 3 equiv.) in chloroform (20 mL), L-proline (29 mg, 0.25 mmol, 0.25 equiv.) and 2-nitrosotoluene (121 mg, 1 mmol, 1 equiv.) were added at -20 °C and the mixture was stirred overnight at -18 °C. The mixture was diluted with pentane (150 mL) and washed with water (40 mL) and brine (40 mL). The organic phase was dried over Na_2SO_4 and concentrated. The resulting yellow oil was dried under high vacuum (5 min) and dissolved in THF (20 mL). The solution of **32** (520 mg, 1 mmol, 1 equiv.) was added via syringe to a cooled mixture of RMgCl (7 equiv.) and $CeCl_3$.2LiCl [prepared according to lit.[6] from anhydrous $CeCl_3$ (1.725 g, 7 mmol, 7 equiv.) and LiCl (593 mg, 14 mmol, 14 equiv) in THF (80 mL)] at -78 ^{o}C. The mixture was allowed to warm to room temperature over a period of 3 h, and stirred at rt overnight. The solvent was evaporated *in vacuo*, and the residue was quenched with saturated aq NH_4Cl (30 mL), followed with 2M HCl (50 mL). The organic layer was separated, and the aqueous layer extracted with ethyl acetate (4 x 100 mL). The combined organic layers were dried over Na_2SO_4 and concentrated. The crude product was purified by flash chromatography (gradient from 0 to 30% of ethyl acetate in hexanes).

33: Yield 99 mg (52%). $[\alpha]_D^{20}$ = 9.1 (*c* = 1.02, $CHCl_3$) [lit.[6] yield 65%, 96% *ee*, $[\alpha]_D^{20}$ = + 6.6 (*c* = 1.23, $CHCl_3$)].

34: Yield 93 mg (45%); $[\alpha]_D^{20}$ = + 10.0 (*c* = 0.1, $CHCl_3$); HRMS: *m/z* (ESI) calculated for $C_{13}H_{18}O_2[M+Na]^+$ 229.1204, found 229.1184.

IR (film, cm^{-1}): ν 3391 (bs), 3303 (s), 2914 (s), 2853 (s), 1604 (s), 1446 (s), 1097 (s), 1039 (s), 965 (s), 805 (s), 777 (s), 699 (s), 472 (s).

^{1}H NMR (300 MHz, $CDCl_3$): δ=7.36–7.20 (m, 5H, Ph), 5.65–5.52 (m, 1H, H-6), 5.51–5.39 (m, 1H, H-5), 3.70 (dd, *J* = 8.6, 4.9 Hz, 1H, H-3), 3.51 (dd, *J* = 8.6, 4.5 Hz, 1H, H-2), 2.90 (dd, *J* = 13.7, 5.0 Hz, 1H, H-1_a), 2.78 (dd, *J* = 13.6, 8.5 Hz, 1H, H-1_b), 2.32–2.23 (m, 2H, H-4), 2.08 (bs, 2H, O$\underline{H}$), 1.69 (dd, *J* = 6.1, 1.2 Hz, 3H, H-7).

^{13}C NMR (75 MHz, $CDCl_3$): δ=138.2 (s, i-Ph), 129.4 (d, C-6), 129.1 (d, C-5), 128.5, 126.6, 126.5 (3xd, Ph), 74.4 (d, C-3), 72.4 (s, C-2), 40.2 (t, C-1), 37.2 (t, C-4), 18.0 (q, C-7).

35: Yield 142 mg (69%); $[\alpha]_D^{20}$ = + 8.5 (*c* = 0.21, $CHCl_3$); HRMS: *m/z* (ESI) calculated for $C_{13}H_{18}O_2$ $[M+Na]^+$ 229.1204, found 229.1212.

IR (film, cm^{-1}): ν 3346 (bs), 3028 (s), 2937 (s), 2913 (s), 1640 (s), 1494 (s), 1405 (s), 1102 (s), 1041 (s), 999 (s), 944 (s), 914 (s), 700 (s).

^{1}H NMR (300 MHz, $CDCl_3$): δ=7.37–7.18 (m, 5H, Ph), 4.91–4.88 (m, 1H, H-6$_a$), 4.82 (dq, *J* = 2.1, 1.0 Hz, 1H, H-6$_b$), 3.76–3.62 (m, 2H, H-2, H-3), 2.92 (dd, *J* = 13.7, 4.7 Hz, 1H, H-1$_a$), 2.82 (dd, *J* = 13.6, 8.1 Hz, 1H, H-1$_b$), 2.33–2.26 (m, 2H, H-4), 2.13–2.05 (m, 2H, O<u>H</u>), 1.74 (bs, 3H, C<u>H</u>$_3$).

^{13}C NMR (75 MHz, $CDCl_3$): δ=142.3 (s, i-Ph), 138.2 (t, C-6), 129.4, 128.6, 126.5 (3xd, Ph), 113.7 (s, C-5), 74.6 (d, C-3), 70.2 (d, C-2), 42.3 (t, C-1), 40.4 (t, C-4), 22.3 (q, <u>C</u>H$_3$).

36: A mixture of diol **33** (8.55 g, 44.5 mmol), *p*-toluenesulfonic acid monohydrate (1.6 g, 8.9 mmol, 0.2 equiv.) in in dry acetone (200 mL) was stirred over 3 h at rt. The neutralisation with K_2CO_3 (10 g, 2 h stirring) and concentration provided acetonide (9.6 g) which was dissolved in the mixture of dioxane/H_2O (200 mL, 1:1). *N*-Methylmorpholine *N*-oxide (8.8 g, 82.6 mmol, 2 equiv.), OsO_4 (104 mg, 0.413 mmol, 0.01 equiv.) and pyridine (1 mL) were added. After stirring for 5 d at room temperature, the mixture was concentrated *in vacuo*, and the residue partitioned between H_2O (200 mL) and ethyl acetate (3 x 170 mL). Organic phase was washed with brine (100 mL), dried over Na_2SO_4 and concentrated to give a crude black viscous oil (11.9 g), which was dissolved in aqueous MeOH (200 mL, 75%). $NaIO_4$ (19.1 g, 90 mmol, 2 equiv.) was added in one portion and vigorously stirred for 3 h at room temperature. After solvent removal *in vacuo*, H_2O (200 mL) was added and extracted with diethyl ether (3 x 200 mL). Combined organic layer was washed with brine (100 mL), dried over Na_2SO_4 and concentrated to give a crude aldehyde (8.5 g) as light yellow oil which was used in the next reaction without further purification. For Horner-Wadsforth-Emmons olefination, sodium hydride (3.12 g, 70 mmol, 60% in mineral oil) was dispersed in THF (200 mL), cooled to -20 °C and neat triethyl phosphonoacetate (17.23 g, 80 mmol) was dropped. After stirring the mixture for 30 min rt, a solution of aldehyde (8.5 g) in THF (100 mL) was added, and stirred for 15 min. at rt. The solvent was removed *in vacuo*. The residue was diluted with ethyl acetate (400 mL), and consecutive washed with water (2 x 130 mL), brine (130 mL), dried over Na_2SO_4. Evaporation of solvent gave corresponding ethyl acrylate (12.1 g) which was subjected to DIBAL reduction in dichloromethane (300 mL). Diisobutylaluminium hydride (1M in CH_2Cl_2, 120 mL, 120 mmol) was added at -30 °C over 30 min. The mixture was stirred 45 min at -10 °C and quenched with sat. Rochelle salt solution (250 mL). After stirring at rt overnight, the mixture was transferred to a separation funnel and the layers were separated. Water phase was extracted with diethyl ether (2 x 200 mL). Combined organic layer was dried over Na_2SO_4 concentrated *in vacuo*. The crude oil was purified by flash chromatography (silica gel, gradient from 0 to 30% of ethyl acetate in hexanes) to give **36** (4.11 g, 39% over 5 steps); HRMS: *m/z* (ESI) calculated for $C_{16}H_{22}O_3$ $[M+Na]^+$ 285.1467, found 285.1469; $[\alpha]_D^{20}$ = + 6.2 (*c* = 0.082, $CHCl_3$).

IR (film, cm^{-1}): ν 3401 (bs), 2032 (s), 2871 (s), 1700 (s), 1609 (s), 1453 (s), 1270 (s), 1206 (s), 1090 (s), 1069 (s), 910 (s), 697 (s), 696 (s).

^{1}H NMR (300 MHz,$CDCl_3$): δ=7.38–7.19 (m, 5H, Ph), 5.72–5.53 (m, 2H, H-2',H-3'), 4.11–4.03 (m, 2H, H-4, H-5), 3.91 (ddd, *J* = 7.9, 6.2 Hz, 1H, H-4'$_a$), 3.77 (ddd, *J* = 8.0, 5.7 Hz, 1H, H-4'$_b$), 2.99 (dd, *J* = 13.9, 6.4 Hz, 1H, Ph-C<u>H</u>$_2$-), 2.81 (dd, *J* = 13.9, 5.9 Hz, 1H, Ph-C<u>H</u>$_2$-), 2.10 (m, 2H, H-1'), 1.50 (bs, 1H, O<u>H</u>), 1.38 (s, 3H, C<u>H</u>$_3$), 1.38 (s, 3H, C<u>H</u>$_3$).

^{13}C NMR (75 MHz, $CDCl_3$): δ=137.3 (s, i-Ph), 131.8 (d, C-3'), 129.3 (d, C-2'), 128.4, 127.8, 126.5 (3xd, Ph), 108.3 (s, C-2), 80.6 (d, C-4), 79.9 (d, C-5), 63.4 (t, C-4'), 39.4 (t, Ph-<u>C</u>H$_2$-), 35.5 (t, C-1'), 27.2 (q, <u>C</u>H$_3$), 27.2 (q, <u>C</u>H$_3$).

37: To a solution of diol **36** (2.45 g, 9.4 mmol), pyridine (2.28 mL, 0.028 mmol, 3 equiv.) in CH_2Cl_2 (30 mL) the acetanhydride (1.77 mL, 18 mmol, 2 equiv.) was added and the mixture was stirred at rt for 3 h. The mixture was diluted with diethylether (100 mL) and washed with water (40 mL) and brine (40 mL). Organic phase was dried over sodium sulphate and concentrated to give a crude oil which was dissolved in 60% AcOH (40 mL) and heated to 60 °C over 3 h. The acetic acid was removed *in vacuo* and the crude product was purified by FLC (silica gel, gradient from 0 to 50% EtOAc in hexanes). Yield 1.91 g, (77% over two steps); HRMS: *m/z* (ESI) calculated for $C_{15}H_{20}O_4$ $[M+Na]^+$ 287.1259, found 287.1261;$[\alpha]_D^{20}$ = + 13.2 (*c* = 0.051, $CHCl_3$).

IR (film, cm^{-1}): ν 3401 (bs), 2030 (s), 2870 (s), 1700 (s), 1610 (s), 1455 (s), 1270 (s), 1210 (s), 1090 (s), 1069 (s), 910 (s), 698 (s), 696 (s).

^{1}H NMR (300 MHz, $CDCl_3$): δ=7.36 – 7.21 (m, 5H, Ph), 5.89 – 5.77 (m, 1H, H-6), 5.68 (dt, *J* = 12.6, 6.1 Hz, 1H, H-5), 4.55 – 4.54 (m, 1H, H-3), 4.53 – 4.52 (m, 1H, H-2), 3.71 (td, *J* = 8.8, 4.5 Hz, 1H, H-7$_a$), 3.58 (dt, *J* = 9.3, 4.8

Hz, 1H, H-7_b), 2.90 (dd, J = 13.7, 4.7 Hz, 1H, , H-1_a), 2.77 (dd, J = 13.6, 8.4 Hz, 1H, H-1_b), 2.40 – 2.32 (m, 2H, H-4), 2.24 (bs, 1H, O$\underline{H}$), 2.23 (bs, 1H, O$\underline{H}$), 2.1 (s, O-CO-$\underline{C}H_3$).

^{13}C NMR (75 MHz, $CDCl_3$): δ=171.0 (s, O-$\underline{C}$O-CH_3), 138.0 (s, i-Ph), 131.6 (d, C-6), 129.5 (d, C-5), 128.8, 127.4, 126.7 (3xd. Ph), 74.5 (d, C-3), 72.4 (d, C-2), 65.0 (t, C-7), 40.3 (t, C-1), 37.0 (t, C-4), 21.1 (q, O-CO-$\underline{C}H_3$).

rac-**40**: To a solution of *cis*-alkene **38** (17.9 g, 0.1 mol) in dichloromethane (300 mL) *m*-chloroperbenzoic acid [a freshly prepared solution from 31.2 g of MCPBA (70%) by extraction to CH_2Cl_2 (250 mL)] was added at 0 °C over 15 min. After 30 min at rt, the reaction was quenched with sat. aq $Na_2S_2O_3$ (150 mL) and washed with sat. aq $NaHCO_3$ (2 x 100 mL). Organic phase was washed with brine (50 mL), dried over Na_2SO_4 and concentrated to give a crude racemic epoxide 21.56 g which was dissolved in THF (200 mL). H_2O (200 mL) and TFA (1 mL) were added. After 12 h at 60 °C, the mixture was concentrated *in vacuo*. The reminder was diluted with toluene (150 mL) and concentrated to remove rest of water. The residue, viscous light yellow oil (25 g) was stirred for 3 h at rt in dry acetone (400 mL) in the presence of molecular sieves (4A, 25 g) and *p*-toluenesulfonic acid monohydrate (2.1g, 0.02 mol). $NaHCO_3$ (20 g) was added and the mixture was stirred for another 2 h. Solid was filtered off and the filtrate concentrated. The crude *O*-acetonide-protected acetate (29.5 g, yellow oil) was treated with sodium methoxide (8 g, 1.4 equiv.) in methanol (300 mL). After 48 h at rt, HCl (36%, 3 mL) was dropped to the mixture (pH 7). The solvent was removed *in vacuo*, and the residue distributed between ethyl acetate (600 mL) and H_2O (200 mL). The organic layer was dried over Na_2SO_4 and concentrated. Purification by flash column chromatography on silica gel (0% to 20% ethyl acetate/hexanes) gave *rac*-**40** (8.6 g, 43% over 5 steps) as colourless oil. HRMS: *m/z* (ESI) calculated for $C_{12}H_{24}O_3$ $[M+Na]^+$ 239.1623, found 239.1628.

IR (film, cm^{-1}): ν 3375 (bs), 3030 (s), 2870 (s), 1454 (s), 1392 (s), 1059 (s), 1046 (s), 995 (s), 879 (s), 736 (s), 697 (s), 602 (s), 471 (s).

^1H NMR (300 MHz, $CDCl_3$): δ=3.87–3.63 (m, 4H, H-4, H-5, -CH_2-C$\underline{H}_2$-OH), 2.47 (t, J = 5.0 Hz, 1H, O$\underline{H}$), 1.90–1.68 (m, 3H, -C$\underline{H}_2$-CH_2-OH, H-1'$_a$), 1.58–1.46 (m, 3H, H-1'$_b$, H-2'), 1.39 (s, 6H, $(C\underline{H}_3)_2C$), 1.36–1.27 (m, 4H, H-3', H-4'), 0.89 (t, J = 6.8 Hz, 3H, H-5').

^{13}C NMR (75 MHz, $CDCl_3$): δ=108.4 (s, C-2), 81.1 (d, C-5), 80.3 (d, C-4), 61.1 (t, -CH_2-$\underline{C}H_2$-OH), 34.8 (t, -$\underline{C}H_2$-CH_2-OH), 32.6 (t, C-1'), 32.0 (t, C-3'), 27.4 (q, $\underline{C}H_3$), 27.3 (q, $\underline{C}H_3$), 25.8 (t, C-2'), 22.6 (t, C-4'), 14.1 (q, C-5').

rac-**41**: A solution of DMSO (10.93 g, 120 mmol, 3.5 equiv.) in CH_2Cl_2 (130 mL) was slowly added to oxalyl chloride (15.27 g, 120 mmol, 3 equiv.) in CH_2Cl_2 (130 mL) at –78 °C and stirred for 45 min under Ar. A solution of *rac*-**40** (8.64 g, 40 mmol) in CH_2Cl_2 (130 mL) was added dropwise at –78 °C. After 30 min stirring at -78 °C Et_3N (33.4 ml, 240 mmol, 6 equiv.) was added. After 1 h at –78 °C, the mixture was allowed to warm to room temperature over 1 h. The mixture was concentrated *in vacuo*, diluted with ether (400 mL) and washed with H_2O (2 x 150 mL). The organic phase was dried over Na_2SO_4 and concentrated. The crude aldehyde (9.82 g, R_f = 0.3, EtOAc/hexanes 1:4) was used in the next step without further purification. To a solution of diethyl allyphosphonate (9.8 g, 50 mmol, 1.2 equiv.) in THF (80 mL) BuLi (2.5 M in hexanes, 22 mL, 55 mmol) was slowly added at -78 °C. After stirring the mixture for 1 h at -78 °C, hexamethylenephosphor triamide (16 mL) and a solution of the crude aldehyde (9.82 g) in THF (50 mL) were added at room temperature and stirred at this temperature overnight. Volatiles were removed by concentration *in vacuo*. H_2O (200 mL) was added to a residue and extracted with diethyl ether (3 x 150 mL). The combined organic fraction was washed with brine (100 mL), then dried over Na_2SO_4 and concentrated. The residue was purified on silica gel (0% to 4% ethyl acetate/hexanes) to give diene *rac*-**41** (3 g, 30%) as yellow oil. HRMS: *m/z* (ESI) calculated for $C_{15}H_{26}O_2$ $[M+Na]^+$ 261.1831, found 261.1839.

IR (film, cm^{-1}): ν 2953 (bs), 2884 (s), 2856 (s), 1722 (s), 1471 (s), 1253 (s), 1090 (s), 1068 (s), 991 (s), 925 (s), 834 (s), 776 (s), 697 (s).

^1H NMR (300 MHz, $CDCl_3$): δ=6.33 (dt, J = 16.9, 10.2 Hz, 1H, H-4'), 6.13 (dd, J = 15.1, 10.4 Hz, 1H, H-3'), 5.81–5.67 (m, 1H, H-2'), 5.18 – 4.98 (m, 2H, H-5'), 3.73–3.58 (m, 2H, H-4, H-5), 2.43–2.31 (m, 2H, H-1'), 1.55–1.45 (m, 4H, H-1'', H-2'), 1.42–1.35 (m, 6H, $(C\underline{H}_3)_2C$), 1.35–1.27 (m, 4H, H-3'', H-4''), 0.89 (t, J = 6.7 Hz, 3H, H-5'').

^{13}C NMR (75 MHz, $CDCl_3$): δ=137.0 (d, C-3'), 133.5 (d, C-2'), 130.0 (d, C-4'), 116.0 (t, C-5'), 108.1 (s, C-2), 80.6 (d, C-4), 80.3 (d, C-5), 36.0 (t, C-1'), 32.9 (t, C-1''), 32.0 (t, C-3''), 27.4 (q, $\underline{C}H_3$), 27.3 (q, $\underline{C}H_3$), 25.8 (t, C-2''), 22.6 (t, C-4''), 14.1 (t, C-5'').

rac-**42**: A solution of *rac*-**41** (3g, 12.59 mmol) in 60% AcOH (70 mL) was heated at 60 °C for 3 h. The solvents were removed *in vacuo* (45 °C, 18 mbar) and the crude product was purified by flash chromatography (silica gel, 0% to 23% EtOAc/hexanes) to give diol *rac*-**42** (1.577 g, 63%) as light yellow oil. HRMS: *m/z* (ESI) calculated for $C_{12}H_{22}O_2$ $[M+Na]^+$ 221.1517, found 221.1617.

IR (film, cm^{-1}): ν 3400 (bs), 2030 (s), 2861 (s), 1722 (s), 1719 (s), 1453 (s), 1270 (s), 1206 (s), 1093 (s), 1069 (s), 912 (s), 697 (s), 608 (s).

^{1}H NMR (300 MHz, $CDCl_3$): δ= 6.34 (dt, *J* = 16.8, 10.2 Hz, 1H, H-3), 6.16 (dd, *J* = 15.1, 10.4 Hz, 1H, H-2), 5.79–5.67 (m, 1H, H-4), 5.21–4.99 (m, 2H, H-1), 3.58–3.38 (m, 2H, H-6, H-7), 2.52–2.21 (m, 2H, H-5), 2.10 (bs, 2H. O$\underline{H}$), 1.56–1.41 (m, 3H, H-8, H-10_a), 1.39–1.26 (m, 5H, H-8, H-10_b, H-11), 0.90 (t, *J* = 6.7 Hz, 3H, H-12).

^{13}C NMR (75 MHz, $CDCl_3$): δ=136.2 (d, C-3), 133.8 (d, C-2), 129.6 (d, C-4), 115.7 (t, C-1), 73.4 (d, C-6), 73.1 (d, C-7), 36.6 (t, C-5), 33.1 (t, C-8), 31.3 (t, C-10), 24.8 (t, C-9), 22.1 (t, C-11), 13.6 (q, C-12).

Palladium-catalysed cyclisations of polyols

General procedure (Method A), **45**: The mixture of alkenol **12** (416 mg, 1.167 mmol), $PdCl_2$ (20 mg, 0.116 mmol, 0.1 equiv.), $CuCl_2$ (466 mg, 3.501 mmol, 3 equiv.) and AcONa (288 mg, 3.501 mmol, 3 equiv.) in glacial AcOH (7 mL) were stirred at 25-30 °C for 23 h. Solvent was evaporated *in vacuo* and the residue distributed between 10% aq NH_3 (10 mL) and AcOEt (10 mL). Water phase was extracted with AcOEt (2 x 15 mL) and combined org. layers were washed with brine (10 mL), dried (Na_2SO_4) and concentrated. Purification of the crude product by flash chromatography (10% AcOEt in hexanes) yielded bicycle **45** (173 mg, 63%) as colourless oil; HRMS: *m/z* (ESI) calculated for $C_{21}H_{26}O_3Si$ $[M+Na]^+$ 377.1549, found 377.1556; $[\alpha]_D^{20}$ = + 25.1 (*c* = 0.213, $CHCl_3$).

IR (film, cm^{-1}): ν 2985 (s), 2929 (s), 2866 (s), 2931 (s), 1644 (s), 1454 (s), 1373 (s), 1213 (s), 1072 (s), 1023 (s), 862 (s), 737 (s), 698 (s).

^{1}H NMR (300 MHz, $CDCl_3$): δ=7.75–7.63 (m, 4H, Ph), 7.51–7.35 (m,6H, Ph), 4.30 (d, *J* = 2.7 Hz, 1H, H-7), 4.25 (d, *J* = 7.5 Hz, 1H, H-3), 4.02–3.92 (m, 3H, H-1_a, H-4_a, H-6), 3.82 (d, *J* = 8.4 Hz, 1H, H-1_b), 3.73 (d, *J* = 8.3 Hz, 1H, H-4_b), 1.07 (s, 9H, $(C\underline{H}_3)_3C$-Si).

^{13}C NMR (75 MHz, $CDCl_3$): δ=135.5 (s, i-Ph), 135.5 (s, i-Ph), 133.2, 132.9, 130.0, 129.9, 127.8, 127.8 (6xd, Ph), 77.6 (d, C-3), 77.3 (d, C-6), 74.6 (d, C-7), 73.9 (t, C-1), 72.9 (t, C-4), 26.7 (q, $(\underline{C}H_3)_3C$-Si), 19.1 (s, $(CH_3)_3\underline{C}$-Si).

46: According to general procedure, mixture of *E*-**21** (300 mg, 1.07 mmol), $PdCl_2$ (19 mg, 0.11 mmol), $CuCl_2$ (432 mg, 3.21 mmol), AcONa (264 mg, 3.21 mmol) in glacial AcOH (10 mL), 60 ^{o}C, 24 h. Yield: 90 mg (30%) as colourless oil; HRMS: *m/z* (ESI) calculated for $C_{15}H_{18}O_5$ $[M+Na]^+$ 301.1052 found 301.1022; $[\alpha]_D^{20}$ = - 72.2 (*c* = 0.419, $CHCl_3$).

IR (film, cm^{-1}): ν 3450 (bs), 3025 (s), 2930 (s), 2860 (s), 1725 (s), 1700 (s), 1161 (s), 1046 (s), 937 (s), 744 (s), 699 (s), 668 (s), 545 (s).

^{1}H NMR (300 MHz, $CDCl_3$): δ= 7.40–7.27 (m, 5H, Ph), 5.59 (s, 1H, H-1'), 5.26 (s, 1H, H-3), 4.76 (d, *J* = 11.2 Hz, 1H, O-C$\underline{H}_2$-Ph), 4.70 (d, *J* = 11.2 Hz, 1H, O-C$\underline{H}_2$-Ph), 4.42 (dd, *J* = 9.9, 3.0 Hz, 1H, H-4), 4.36–4.31 (m, *J* = 15.1 Hz, 1H, H-5_a), 4.21–4.10 (m, 3H, H-5_b, CH_3-C$\underline{H}_2$-O), 2.23 (bs, 1H, O$\underline{H}$), 1.28 (t, *J* = 7.1 Hz, 3H, C$\underline{H}_3$-CH_2-O).

^{13}C NMR (151 MHz, $CDCl_3$): δ=171.5 (s, C-2), 167.7 (s, C-2'), 137.9 (s, i-Ph), 128.3, 127.9, 127.8 (3xd, Ph), 95.4 (d, C-1'), 81.0 (d, C-3), 76.9 (d, C-4), 73.4 (t, C-5), 72.5 (t, O-$\underline{C}H_2$-Ph), 59.8 (t, CH_3-$\underline{C}H_2$-O), 14.3 (q, $\underline{C}H_3$-CH_2-O).

47 and **48**: A mixture of $PdCl_2$ (17 mg, 0.14 mmol, 0.1 equiv.), $CuCl_2$ (567 mg, 4.22 mmol, 3 equiv.), AcONa (346 mg, 4.22 mmol, 3 equiv.) and diol **24** (355 mg, 1.408 mmol) in AcOH (15 ml) was stirred at 60 °C for 12 h. Solvent was evaporated *in vacuo* and the residue distributed between 10% aq NH_3 (25 mL) and AcOEt (35 mL). Water phase was extracted with AcOEt (3 x 35 mL) and combined organic layers were washed with brine (10 mL), dried (Na_2SO_4) and concentrated. Purification of the crude product by flash chromatography (30% AcOEt in hexanes) yielded diastereomers **47** (145 mg, 41%) and **48** (87 mg, 25%).

Data for **47**: HRMS: *m/z* (ESI) calculated for $C_{13}H_{16}O_3$ $[M+Na]^+$ 243.0997, found 243.0990; $[\alpha]_D^{20}$ - 22.1 (*c* = 0.037, $CHCl_3$).

IR (film, cm^{-1}): ν 3440 (s), 3030 (s), 2985 (s), 2861 (s), 1731 (s), 1450 (s), 1375 (s), 1210 (s), 1070 (s), 1027 (s), 975 (s), 855 (s), 690 (s).

^1H NMR (300 MHz, $CDCl_3$): δ=7.40 – 7.26 (m, 5H, Ph), 5.96 (ddd, *J* = 16.8, 10.4, 6.2 Hz, 1H, H-1'), 5.39 (dt, *J* = 17.2, 1.5 Hz, 1H, H-2'$_a$), 5.19 (dt, *J* = 10.4, 1.4 Hz, 1H, H-2'$_b$), 4.62 (s, 2H, 1H, O-C<u>H</u>$_2$-Ph), 4.33 – 4.27 (m, 2H, H-2, H-4), 4.02 (dd, *J* = 10.0, 4.0 Hz, 1H, H-5$_a$), 3.91 (d, *J* = 10.0 Hz, 1H, H-5$_b$), 3.79 – 3.75 (m, 1H, H-3), 1.74 (d, *J* = 6.9 Hz, 1H, O<u>H</u>).

^{13}C NMR (151 MHz, $CDCl_3$): δ=137.6 (s, i-Ph), 136.8 (d, C-1'), 128.5, 127.9, 127.7 (3xd, Ph), 116.4 (t, C-2'), 89.5 (d, C-2), 84.6 (d, C-3), 76.5 (d, C-4), 74.1 (t, O-<u>C</u>H$_2$-Ph), 72.0 (t, C-5).

Data for **48**: HRMS: *m/z* (ESI) calculated for $C_{13}H_{16}O_3$ $[M+Na]^+$ 243.0997, found 243.0999; $[\alpha]_D^{20}$ = -31.6 (*c* = 0.160, $CHCl_3$).

IR (film, cm^{-1}): ν 3430 (s), 3030 (s), 2985 (s), 2865 (s), 1720 (s), 1453 (s), 1370 (s), 1200 (s), 1075 (s), 1025 (s), 975 (s), 856 (s), 690 (s).

^1H NMR (300 MHz, $CDCl_3$): δ=.36 – 7.26 (m, 5H, Ph), 6.03 (ddd, *J* = 17.5, 10.4, 7.3 Hz, 1H, H-1'), 5.38 (dd, *J* = 9.8, 8.6 Hz, 1H, H-2'$_a$), 5.29 (d, *J* = 10.4 Hz, 1H, H-2'$_b$), 4.60 (d, *J* = 2.8 Hz, 2H, O-C<u>H</u>$_2$-Ph), 4.51 (dd, *J* = 7.2, 4.2 Hz, 1H, H-2), 4.39 – 4.36 (m, 1H, H-4), 4.19 (dd, *J* = 9.8, 4.5 Hz, 1H, H-5$_a$), 3.85 (dd, *J* = 4.1, 1.5 Hz, 1H, H-3), 3.70 (dd, *J* = 9.8, 1.9 Hz, 1H, H-5$_b$), 1.81 (bs, 1H, O<u>H</u>).

^{13}C NMR (151 MHz, $CDCl_3$): δ= 137.8 (s, *i*-Ph), 133.6(d, C-1'), 128.4, 127.8, 127.5(3xd, Ph), 118.2 (t, C-2'), 85.7 (d, C-2), 81.7 (d, C-3), 75.5 (d, C-4), 73.4 (t, O-<u>C</u>H$_2$-Ph), 72.2 (t, C-5).

49 and **50**: According to general procedure, benzylated tetraol **28** (230 mg, 0.86 mmol), $PdCl_2$ (15 mg, 0.09 mmol), $CuCl_2$ (348 mg, 2.59 mmol), AcONa (213 mg, 2.59 mmol) in AcOH (15 mL), 12 h at 60 °C. Separation on silica-gel (10% AcOEt in hexanes) provided **49** (75 mg, 35%); HRMS: *m/z* (ESI) calculated for $C_{15}H_{20}O_3$ $[M+Na]^+$ 271.1310, found 271.1320; $[\alpha]_D^{20}$ + 7.7 (*c* = 0.37, $CHCl_3$). IR (film, cm^{-1}): ν 3429 (s), 3030 (s), 2985 (s), 2860 (s), 1721 (s), 1453 (s), 1370 (s), 1211 (s), 1072 (s), 1027 (s), 976 (s), 856 (s), 697 (s). ^1H NMR (6300 MHz, $CDCl_3$): δ=7.37–7.29 (m, 5H, Ph), 5.31 (ddd, *J* = 9.0, 2.6, 1.3 Hz, 1H, H-1'), 4.59 (bs, 2H, O-C<u>H</u>$_2$-Ph), 4.48 (dd, *J* = 8.9, 4.3 Hz, 1H, H-2), 4.30 (dt, *J* = 3.8, 1.7 Hz, 1H, H-4), 3.94 (dt, *J* = 6.9, 3.4 Hz, 1H, H-5$_a$), 3.86–3.83 (m, 1H, H-5$_b$), 3.69 (ddd, *J* = 4.3, 1.4, 1.0 Hz, 1H, H-3), 1.90 (bs, 1H, O<u>H</u>), 1.76 (d, *J* = 1.2 Hz, 3H, C<u>H</u>$_3$), 1.75 (d, *J* = 1.2 Hz, 3H, C<u>H</u>$_3$). ^{13}C NMR (151 MHz, $CDCl_3$): δ=137.8 (s, i-Ph), 137.7 (d, C-1'), 128.4, 127.8, 127.6 (3xd, Ph), 123.4 (s, C-2'), 91.0 (d, C-2), 80.8 (d, C-3), 76.7 (d, C-4), 73.6 (t, O-<u>C</u>H$_2$-Ph), 72.1 (t, C-5), 25.8 (q, <u>C</u>H$_3$), 18.3 (q, <u>C</u>H$_3$). The second fraction with R_f 0.25 contained pure diastereomer **50** (49 mg, 23%); HRMS: *m/z* (ESI) calculated for $C_{15}H_{20}O_3$ $[M+Na]^+$ 271.1310, found 271.1320; $[\alpha]_D^{20}$ = + 5.2 (*c* = 0.092, $CHCl_3$). IR (film, cm^{-1}): ν 3430 (s), 2030(s), 2860 (s), 1720 (s), 1450 (s), 1370 (s), 1210 (s), 1211 (s), 1073 (s), 1033 (s), 860 (s), 737 (s), 690 (s). ^1H NMR (300 MHz, $CDCl_3$): δ= 7.39–7.27 (m, 5H, Ph), 5.49 (dd, *J* = 8.9, 1.2 Hz, 1H, H-1'), 4.76 (dd, *J* = 8.8, 3.9 Hz, 1H, H-2), 4.58 (bs, 2H, O-C<u>H</u>$_2$-Ph), 4.40–4.36 (m, 1H, H-4), 4.20–4.15 (m, 1H, H-5$_a$), 3.78–3.74 (m, 1H, H-5$_b$), 3.64 (dt, *J* = 9.8, 2.0 Hz, 1H, H-3), 2.11 (bs, 1H, O<u>H</u>), 1.79 (bs, 3H, C<u>H</u>$_3$), 1.71 (bs, 3H, C<u>H</u>$_3$). ^{13}C NMR (151 MHz, $CDCl_3$): δ=138.0 (s, *i*-Ph), 137.8(d, C-1'), 128.3, 127.7, 127.5 (3xd, Ph), 119.7 (s, C-2'), 85.4 (d, C-2), 76.8 (d, C-3), 75.9 (d, C-4), 73.1 (t, O-<u>C</u>H$_2$-Ph), 72.2 (t, C-5), 26.0 (q, <u>C</u>H$_3$), 18.4 (q, <u>C</u>H$_3$).

51 and **52**: According to general procedure, *syn*-diol **33** (270 mg, 1.407 mmol), $PdCl_2$ (25 mg, 0.14 mmol), $CuCl_2$ (571 mg, 4.216 mmol), AcONa (345 mg, 4.216 mmol) in AcOH (10 mL), 24 h at 30 °C. After work up

purification (silica gel, gradient from 0 to 30% ethyl acetate in hexanes) yielded bicyclic product **51** (40 mg, 30%) and furan derivative **52** (60 mg, 25%).

Data for **51**: $[\alpha]_D^{20}$ =- 3.1 (c = 0.07, $CHCl_3$).

IR (film, cm^{-1}): ν 3405 (s), 3026 (s), 2928 (s), 2726 (s), 1598 (s), 1453 (s), 1263 (s), 996 (s), 745 (s), 699 (s), 613 (s), 586 (s), 440 (s).

^{1}H NMR (300 MHz,$CDCl_3$): δ=7.32–7.17 (m, 5H, Ph), 4.53 (d, J = 2.6 Hz, 1H, H-1), 4.27 (d, J = 2.9 Hz, 1H, H-4_a), 4.08–4.00 (m, 1H, H-3), 3.91 (d, J = 7.9 Hz, 1H, H-4_b), 3.78 (d, J = 8.4 Hz, 1H, H-6), 3.05–2.92 (m, 2H, H-7), 1.95 (dd, J = 9.9, 2.5 Hz, 1H, H-1'$_a$), 1.88 (dd, J = 10.1, 2.5 Hz, 1H, H-1'$_b$).

^{13}C NMR (75 MHz, $CDCl_3$): δ=138.1 (s, i-Ph), 129.3, 128.4, 126.2 (3xd, Ph), 84.9 (d, C-1), 77.3 (d, C-6), 76.6 (d, C-3), 74.6 (t, C-4), 37.4 (t, C-7), 36.9 (t, C-1').

Spectroscopic data of **52** were in good agreement with those reported in the literature.[7]

53: According to general procedure, diol **35** (350 mg, 1.69 mmol), $PdCl_2$ (31 mg, 0.17 mmol), $CuCl_2$ (688 mg, 5.09 mmol), AcONa (418 mg, 5.09 mmol) in AcOH (15 mL), 4 h at 60 °C, FLC (1% EtOAc in CH_2Cl_2): tetrahydropyran **53** (112 mg, 33%) as colourless oil; HRMS: m/z (ESI) calculated for $C_{13}H_{17}O_2Cl$ $[M+Na]^+$ 263.0815, found 263.0967; $[\alpha]_D^{20}$ = + 4.3 (c = 0.32, $CHCl_3$).

IR (film, cm^{-1}): ν 3442 (bs), 3026 (s), 2932 (s), 2865 (s), 1495 (s), 1450 (s), 1061 (s), 1046 (s), 937 (s), 743 (s), 698 (s), 668 (s), 539 (s).

^{1}H NMR (300 MHz, $CDCl_3$): δ= 7.34–7.19 (m, 5H, Ph), 3.89 (dd, J = 11.2, 2.5 Hz, 1H, H-6_a), 3.78–3.72 (m, 1H, H-3), 3.66–3.59 (m, 1H, H-2), 3.61 (dd, J = 11.3, 0.9 Hz, 1H, H-6_b), 2.96 (dd, J = 13.8, 7.1 Hz, 1H, H-1'$_a$), 2.87 (dd, J = 13.8, 7.3 Hz, 1H, H-1'$_b$), 2.33 (ddd, J = 14.1, 3.2, 2.6 Hz, 1H, H-4_a), 2.15 (ddd, J = 14.1, 3.8, 0.8 Hz, 1H, H-4_b), 1.86 (s, 3H, $C\underline{H}_3$), 1.64 (d, J = 5.8 Hz, 1H, O$\underline{H}$).

^{13}C NMR (75 MHz, $CDCl_3$): δ=137.7 (s, i-Ph), 129.2, 128.5, 126.5 (3xd, Ph), 80.9 (t, C-6), 77.6 (d, C-2), 67.2 (d, C-3), 64.4 (s, C-5), 46.4 (t, C-4), 36.7 (t, C-1'), 30.1 (q, $\underline{C}H_3$).

54 and **55**: According to general procedure, mixture of **30** (460 mg, 2.07 mmol), $PdCl_2$ (37 mg, 0.21 mmol), $CuCl_2$ (835 mg, 6.21 mmol), AcONa (509 mg, 6.21 mmol) in AcOH (15 mL), 60 ^{o}C., 4 h, FLC (16% ethyl acetate in hexanes): **54** (201 mg, 38%) and **55** (185 mg, 35%).

Data for **54**: HRMS: m/z (ESI) calculated for $C_{13}H_{17}O_3Cl$ $[M+Na]^+$ 279.0764, found 279.0651; $[\alpha]_D^{20}$ = + 36.2 (c = 0.34, $CHCl_3$).

IR (film, cm^{-1}): ν 3389 (bs), 2929 (s), 2855 (s), 1710 (s), 1470 (s), 1437 (s), 1266 (s), 1101 (s), 1026 (s), 823 (s), 739 (s), 699 (s), 511 (s).

^{1}H NMR (300 MHz, $CDCl_3$): δ=7.46–7.28 (m, 5H, Ph), 5.09 (d, J = 11.3 Hz, 1H, O-C$\underline{H}_2$-Ph), 4.72 (d, J = 11.2 Hz, 1H, O-C$\underline{H}_2$-Ph), 4.00 (ddd, J = 11.2, 5.1, 1.0 Hz, 1H, H-2_a), 3.79 (dd, J = 11.6, 1.0 Hz, 1H, H-6_a), 3.66 (ddd, J = 9.5, 8.3, 5.2 Hz, 1H, H-3), 3.58–3.52 (m, 2H, H-4, H-6_b), 3.26 (dd, J = 11.2, 9.6 Hz, 1H, H-2_b), 2.26 (bs, 1H, O$\underline{H}$); 1.64 (s, 3H, $C\underline{H}_3$).

^{13}C NMR (75 MHz, $CDCl_3$): δ=138.1 (s, i-Ph), 128.6, 128.1, 128.0 (3xd, Ph), 88.2 (d, C-4), 76.4 (t, C-6), 75.8 (t, O-$\underline{C}H_2$-Ph), 70.6 (s, C-5), 68.8 (t, C-2), 68.3 (d, C-4), 22.5 (q, $\underline{C}H_3$).

Data for **55**: HRMS: m/z (ESI) calculated for $C_{13}H_{17}O_3Cl$ $[M+Na]^+$ 279.0764, found 279.0712; $[\alpha]_D^{20}$= - 0.3 (c = 0.34, $CHCl_3$).

IR (film, cm^{-1}): ν 3390 (bs), 2929 (s), 2857 (s), 1702 (s), 1470 (s), 1437 (s), 1265 (s), 1100 (s), 1025 (s), 823 (s), 739 (s), 697 (s), 510 (s).

^{1}H NMR (300 MHz, $CDCl_3$): δ=7.39–7.29 (m, 5H, Ph), 4.66 (bs, 2H, O-C$\underline{H}_2$-Ph), 4.37–4.34 (m, 1H, H-4), 4.05 (dd, J = 9.9, 5.2 Hz, 1H, H-5_a), 3.91 (d, J = 2.8 Hz, 1H, H-3), 3.72 (dd, J = 9.9, 3.7 Hz, 1H, H-5_b), 3.64 (d, J = 11.2 Hz, 1H, H-1'$_a$), 3.50 (d, J = 11.2 Hz, 1H, H-1'$_b$), 2.02 (bs, 1H, O$\underline{H}$), 1.33 (s, 3H, $C\underline{H}_3$).

^{13}C NMR (151 MHz, $CDCl_3$): δ=137.9 (s, i-Ph), 128.5, 127.9, 127.5 (3xd, Ph), 87.3 (d, C-3), 84.3 (s, C-2), 76.8 (d, C-4), 72.8 (t, O-$\underline{C}H_2$-Ph), 71.7 (t, C-5), 50.1 (t, C-1'), 18.9 (q, $\underline{C}H_3$).

56 and **57**: A solution of diol **37** (1.00 mg, 3.79 mmol), $Pd(PPh_3)_4$ (437mg, 0.379 mmol, 0.1 equiv.) and PPh_3 (199 mg, 0.758 mmol, 0.2 equiv.) in dry THF (10 mL) was refluxed for 5 h. The solvent was removed *in vacuo* and the residue purified by FLC (silica gel, gradient from 0 to 30% EtOAc in hexanes) to provide 772 mg (84%) of viscous oil as a mixture of two inseparable diastereomers **56** and **57** in the ratio 1:3.

Data for the minor isomer: ^{1}H NMR (300 MHz, $CDCl_3$, mixture of **56**/**57** in the ratio 1/3): δ=7.37–7.23 (m, 5H, Ph), 6.11–5.96 (m, 1H, H-1'), 5.40–5.14 (m, 2H, H-2'), 4.45–4.34 (m, 1H, H-2), 4.14–4.13 (m, 1H, H-5), 3.93 (ddd, J = 7.1, 3.1 Hz, 1H, H-3), 3.09–2.96 (m, 1H, Ph-C$\underline{H_2}$), 2.48–2.36 (m, 1H, Ph-C$\underline{H_2}$), 1.99 (bs, 1H, O$\underline{H}$), 1.86–1.77 (m, 1H, H-4_a), 1.64 (d, J = 8.1 Hz, 1H, H-4_b).

^{13}C NMR (151 MHz, $CDCl_3$): δ=138.51 (s, i-Ph), 137.95 (d, C-1'), 128.93, 128.09, 125.91 (3xd, Ph), 114.88 (t, C-2'), 82.95 (d, C-2), 77.75 (d, C-5), 71.84 (d, C-3), 42.09 (t, C-4), 34.02 (t, Ph-$\underline{C}H_2$).

Data for the major isomer: ^{1}H NMR (300 MHz, $CDCl_3$): δ=7.33–7.20 (m, 5H, Ph), 5.85 (ddd, J = 16.9, 10.2, 6.5 Hz, 1H, H-1'), 5.30–5.05 (m, 2H, H-2'), 4.72 (dd, J = 15.6, 6.7 Hz, 1H, H-2), 4.21–4.15 (m, 1H, H-3), 4.11 (ddd, J = 9.3, 6.5, 2.8 Hz, 1H, H-5), 3.06–2.98 (m, 2H, Ph-C$\underline{H_2}$), 2.16 (ddd, J = 13.5, 6.5, 1.0 Hz, 1H, H-4_a), 1.92–1.84 (m, 1H, H-4_b), 1.67 (d, J = 6.0 Hz, 1H, O$\underline{H}$).

^{13}C NMR (151 MHz, $CDCl_3$): δ=138.5 (s, i-Ph), 137.9 (d, C-1'), 128.7, 128.0, 125.9 (3xd, Ph), 114.8 (t, C-2'), 82.9 (d, C-2), 77.7 (d, C-5), 72.3 (d, C-3), 41.4 (t, C-4), 34.9 (t, Ph-$\underline{C}H_2$).

General procedure (Method B), **52**: A solution of *syn*-diol **33** (550 mg, 2.86 mmol) in THF (5 mL) was treated with BuLi (2M in hexanes, 2.86 mL, 5.73 mmol, 2 equiv.) at 0 °C and stirred for additional 5 min at rt. A solution of $CuCl_2$ (1154 mg, 8.59 mmol, 3 equiv.) in 0,5M LiCl in THF (17 mL) and $PdCl_2(MeCN)_2$ (75 mg, 0.29 mmol, 0.1 equiv.) were added sequentially. After stirring at rt overnight, the mixture was diluted with EtOAc (100 mL) and 10% aq. NH_3 (25 mL) was added. Organic layer was washed with brine (25 mL), dried (Na_2SO_4) and concentrated. Purification by flash chromatography (gradient from 0 to 5% ethyl acetate in hexanes) yielded furan derivative **52**[7] (320 mg, 65%).

55: Prepared according to Method B, from **30** (300 mg, 1.35 mmol) using BuLi (2M in hexanes, 1.35 mL, 2.7 mmol), a solution of $CuCl_2$ (544 mg, 4.05 mmol) in 0,5M LiCl in THF (15 mL) and $PdCl_2(MeCN)_2$ (35 mg, 0.14 mmol. After work-up and purification by flash chromatography (gradient from 0 to 25% ethyl acetate in hexanes) the title compound **55** (243 mg, 70%) was isolated; HRMS: *m/z* (ESI) calculated for $C_{13}H_{17}O_3Cl$ $[M+Na]^+$ 279.0764, found 279.0712; $[\alpha]_D^{20}$ = - 0.3 (c = 0.34, $CHCl_3$).

IR (film, cm^{-1}): ν 3390 (bs), 2929 (s), 2857 (s), 1702 (s), 1470 (s), 1437 (s), 1265 (s), 1100 (s), 1025 (s), 823 (s), 739 (s), 697 (s), 510 (s).

^{1}H NMR (300 MHz, $CDCl_3$): δ=7.39–7.29 (m, 5H, Ph), 4.66 (bs, 2H, O-C$\underline{H_2}$-Ph), 4.37–4.34 (m, 1H, H-4), 4.05 (dd, J = 9.9, 5.2 Hz, 1H, H-5_a), 3.91 (d, J = 2.8 Hz, 1H, H-3), 3.72 (dd, J = 9.9, 3.7 Hz, 1H, H-5_b), 3.64 (d, J = 11.2 Hz, 1H, H-$1'_a$), 3.50 (d, J = 11.2 Hz, 1H, H-$1'_b$), 2.02 (bs, 1H, O$\underline{H}$), 1.33 (s, 3H, C$\underline{H_3}$).

^{13}C NMR (151 MHz, $CDCl_3$): δ=137.9 (s, i-Ph), 128.5, 127.9, 127.5 (3xd, Ph), 87.3 (d, C-3), 84.3 (s, C-2), 76.8 (d, C-4), 72.8 (t, O-$\underline{C}H_2$-Ph), 71.7 (t, C-5), 50.1 (t, C-1'), 18.9 (q, $\underline{C}H_3$).

58: According to Method B, from *threo*-**9** (200 mg, 0.96 mmol), BuLi (2M in hexanes, 0.96 mL, 1.92 mmol), $CuCl_2$ (387 mg, 2.88 mmol), $PdCl_2(MeCN)_2$ (25 mg, 0.1 mmol) in 0,5M LiCl in THF (15 mL) the chloroderivative **58** (178 mg, 77%) was prepared as a colourless oil; HRMS: *m/z* (ESI) calculated for $C_{12}H_{15}O_3Cl$ $[M+Na]^+$ 265.0607, found 265.0601; $[\alpha]_D^{20}$ = +15.6 (c = 0.550, $CHCl_3$).

IR (film, cm^{-1}): ν 3392 (s), 2929 (s), 2856 (s), 1705 (s), 1471 (s), 1427 (s), 1265 (s), 1105(s), 1027 (s), 822 (s), 738 (s), 699 (s), 503 (s).

^{1}H NMR (300 MHz, $CDCl_3$): δ=7.39–7.29 (m, 5H, Ph), 4.66 (bs, 2H, O-CH_2-Ph), 4.37–4.34 (m, 1H, H-4), 4.05 (dd, J = 9.9, 5.2 Hz, 1H, H-5_a), 3.91 (d, J = 2.8 Hz, 1H, H-3), 3.72 (dd, J = 9.9, 3.7 Hz, 1H, H-5_b), 3.64 (d, J = 11.2 Hz, 1H, H-1'$_a$), 3.50 (d, J = 11.2 Hz, 1H, H-1'$_b$), 2.02 (bs, 1H, OH), 1.33 (s, 3H, CH_3).

^{13}C NMR (151 MHz, $CDCl_3$): δ=137.9 (s, i-Ph), 128.5, 127.9, 127.5 (3xd, Ph), 87.3 (d, C-3), 84.3 (s, C-2), 76.8 (d, C-4), 72.8 (t, O-CH_2-Ph), 71.7 (t, C-5), 50.1 (t, C-1'), 18.9 (q, CH_3).

General procedure (Method C), **45**: To a mixture of $Pd(OAc)_2$ (21 mg, 0.093 mmol, 0,08 equiv.), Me_4NCl (128 mg, 1.16 mmol, 1 equiv.), AcONa (191 mg, 2,334 mmol, 2 equiv.) and $PhI(OAc)_2$ (789 mg, 2.45 mmol, 2,1 equiv.) a solution of **12** (416 mg, 1.167 mmol) in glacial acetic acid (7 mL) was added and the mixture was left to stir overnight at rt. The solvent was removed *in vacuo* and the residue diluted with H_2O (10 mL) and extracted with EtOAc (3 x 15 mL). The organic layer was dried (Na_2SO_4) and concentrated. Purification of the crude product by flash chromatography (10% AcOEt in hexanes) yielded bicycle **45** (165 mg, 40%) as colourless oil.

60: To a solution of diastereomers **56** and **57** (692 mg, 2.64 mmol, **56**/**57**, 1/3) in acetonitrile (10 mL) the iodine (2010 mg, 7.92 mmol, 3 equiv.) was added and left to stir at rt overnight. The mixture was diluted with EtOAc (100 mL), washed with sat. $Na_2S_2O_3$ (40 mL), brine (40 mL) and dried over Na_2SO_4. Purification by flash chromatography (gradient from 0 to 10% ethyl acetate in hexanes) yielded bicyclic derivative **60** (104 mg, 52%, calculated to *syn*-diastereomer **56**). HRMS: *m/z* (ESI) calculated for $C_{13}H_{15}O_2I$ $[M+Na]^+$ 350.0014, found 353.0003; $[\alpha]_D^{20}$ = - 12.3 (c = 0.420, $CHCl_3$).

IR (film, cm^{-1}): ν 3028 (s), 2927 (s), 2856 (s), 1700 (s), 1641 (s), 1471 (s), 1250 (s), 1093(s), 1090 (s), 826 (s), 774 (s), 698 (s), 503 (s).

^{1}H NMR (300 MHz, $CDCl_3$): δ= 7.36 – 7.19 (m, 5H, Ph), 4.70 (d, J = 6.8 Hz, 1H, H-7), 4.49 – 4.44 (m, 1H, H-3_a), 4.33 – 4.29 (m, 2H, H-1, H-7), 4.11 – 4.07 (m, 2H, H-3_b, H-5), 3.20 (dd, J = 13.5, 6.1 Hz, 1H, H-1'$_a$), 3.12 (dd, J = 13.5, 8.7 Hz, 1H, H-1'$_b$), 2.11 (dd, J = 12.3, 1.5 Hz, 1H, H-8_a), 2.05 (ddd, J = 12.3, 6.8, 2.6 Hz, 1H, H-8_b).

^{13}C NMR (151 MHz, $CDCl_3$): δ=138.1 (s, i-Ph), 129.4, 128.5, 126.5 (3xd, Ph), 84.3 (d, C-5), 79.7 (d, C-7), 73.2 (d, C-1), 69.0 (t, C-3), 40.7 (t, C-8), 34.7 (t, C-1'), 29.5 (d, C-4).

59: To a suspension of hexanes-washed NaH (60% in paraffin, 22.49 mg, 0.94 mmol) in dry DMF (1 mL) was added the solution of chloroderivative **55** (69 mg, 0.31 mmol) in DMF (2 mL). The reaction mixture was stirred at 50 °C for 2 h and then diluted with EtOAc (100 mL) and washed with water (2 x 30 mL). The organic layer was dried over Na_2SO_4 and concentrated under reduced pressure. The crude product was purified by FLC (silica gel, gradient from 0 to 30% EtOAc in hexanes) to yield bicyclic product **59** (63 mg, 92 %) as a colourless oil. HRMS: *m/z* (ESI) calculated for $C_{13}H_{16}O_3$ $[M+Na]^+$ 243.0997, found 243.0918; $[\alpha]_D^{20}$ = - 101.8 (c = 0.53, $CHCl_3$).

IR (film, cm^{-1}): ν 2984 (s), 2939 (s), 2866 (s), 2932 (s), 1644 (s), 1456 (s), 1375 (s), 1213 (s), 1073 (s), 1033 (s), 862 (s), 737 (s), 688 (s).

^{1}H NMR (300 MHz, $CDCl_3$): δ= 7.39 – 7.29 (m, 5H, Ph), 4.69 (d, J = 12.2 Hz, 1H, O-CH_2-Ph), 4.65 (d, J = 12.2 Hz, 1H, O-CH_2-Ph), 4.12 – 4.10 (m, 1H, H-1_a), 4.09 – 4.07 (m, 1H, H-6), 3.96 (d, J = 8.2 Hz, 1H, H-4_a), 3.85 (d, J = 8.0 Hz, 1H, H-4_b), 3.82 – 3.79 (m, 1H, H-1_b), 3.77 (d, J = 2.3 Hz, 1H, H-7), 1.34 (s, 3H, CH_3).

^{13}C NMR (151 MHz, $CDCl_3$): δ= 137.8(s, i-Ph), 128.5, 128.0, 127.8 (3xd, Ph), 82.5 (d, C-7), 81.3 (d, C-6), 77.6 (s, C-3), 72.8 (t, O-CH_2-Ph), 72.5 (t, C-1, C-4), 12.2 (q, CH_3).

44: Prepared as described above from **58** (100 mg, 0.41 mmol), NaH (60% in paraffin, 40 mg, 1.24 mmol, 3 equiv.) in DMF was added (3 mL). After work-up and purification by flash chromatography (gradient from 0 to 30% ethyl acetate in hexanes) the title compound **44** (77 mg, 90%) was isolated. All physical and spectroscopic data of **44** were in good agreement with those reported in the literature.[2b]

4. References

1. Babjak, M.; Zálupský, P.; Gracza, T. *Arkivoc* **2005**, (v), 45-57.

2. a) Babjak, M.; Remen, L.; Szolcsányi, P.; Zalupsky, P.; Miklos, D.; Gracza, T. *J. Organomet. Chem*. **2006**, *691*, 928–940; b) Babjak, M.; Remen, L.; Karlubíková, O.; Gracza, T. *Synlett* **2005**, 1609–1611; c) Palík, M.; Karlubíková, O.; Lásiková, A.; Kožíšek, J.; Gracza, T. *Eur. J. Org. Chem.* **2009**, 709–715.
3. Chandrasekhaar, S.; Parida, B. B.; Rambabu, Ch. *J. Org. Chem*., **2008**, *73*, 7826–7828.
4. Fürstner, A.; Jumbam, D.; Teslic, J.; Weidmann, H. *J. Org. Chem*. **1991**, *56*, 2213-2217.
5. Li, F.; Li, Zh.-M.; Yang, H.; Jäger, V. *Zeitsch. für Naturforschung, B*: *Chemical Sciences* **2008**, *63*, 431–446.
6. Jiao, P.; Kawasaki, M.; Yamamoto, H. *Angew. Chem. Int. Ed*. **2009**, *48*, 3333-3336.
7. Hall, S. S.; Farahat, S. E. *J. Heterocycl. Chem*. **1987**, *24*, 1205–1213.
