# Supplementary material for: Palladium-catalysed cyclisation of alkenols: Synthesis of oxaheterocycles as core intermediates of natural compounds
Source: Beilstein J Org Chem. 2014 Sep 3;10:2077–86. doi: 10.3762/bjoc.10.216 (PMC4168889; doi:10.3762/bjoc.10.216)
Supplement: File 2 — 1H NMR and 13C NMR spectra of selected compounds. [file Beilstein_J_Org_Chem-10-2077-s002.pdf]

Supporting Information File 2
for

Palladium-catalysed cyclisation of alkenols: Synthesis of oxaheterocycles as core intermediates of natural compounds

Miroslav Palík[1], Jozef Kožíšek[2], Peter Koóš[3] and Tibor Gracza*[1]

Address: [1]Department of Organic Chemistry Slovak University of Technology, Radlinského 9, SK-812 37 Bratislava, Slovakia, [2]Department of Physical Chemistry, Slovak University of Technology, Radlinského 9, SK-812 37 Bratislava, Slovakia and[3]Georganics Ltd., Koreničova 1, SK-811 03 Bratislava, Slovakia

Email: Tibor Gracza - tibor.gracza@stuba.sk

* Corresponding author

^{1}H NMR and ^{13}C NMR spectra of selected compounds

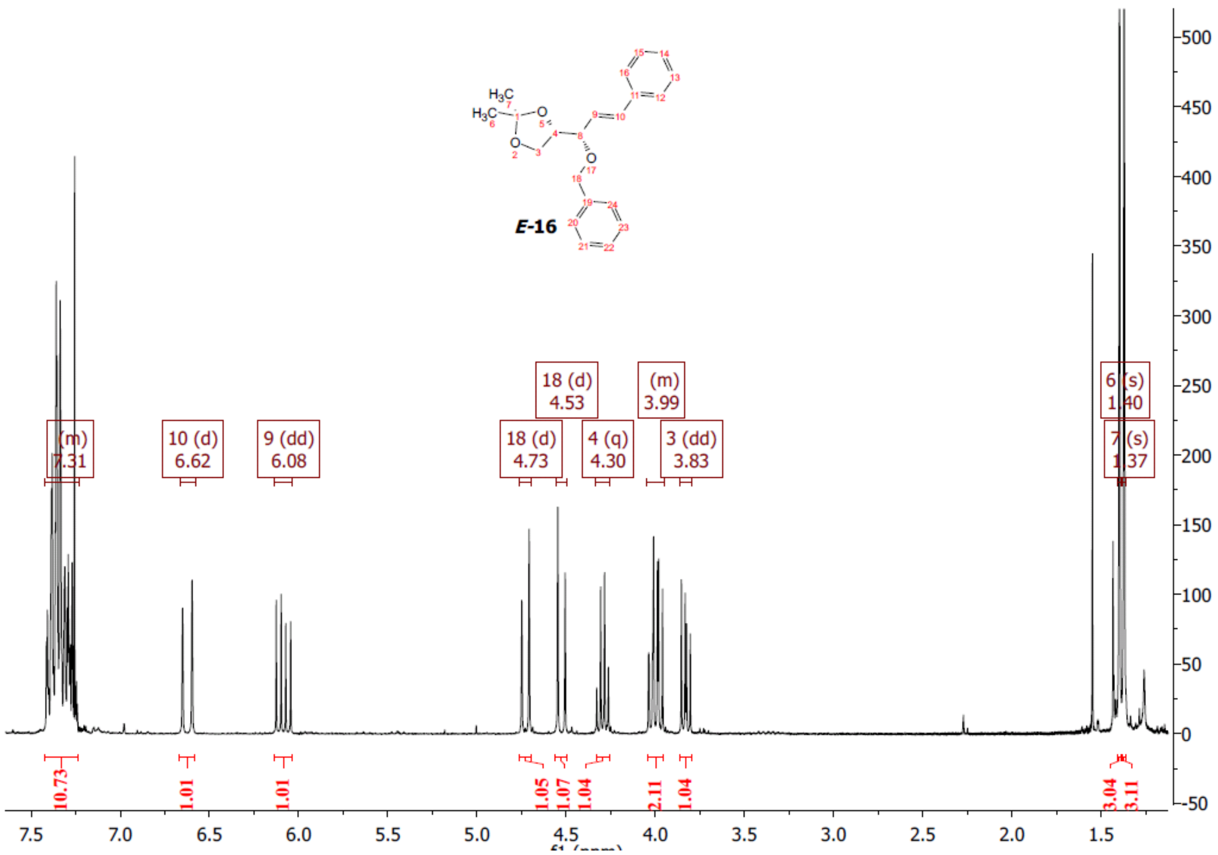

E-16
(m)
7.31
10 (d)
6.62
9 (dd)
6.08
18 (d)
4.73
18 (d)
4.53
4 (q)
4.30
(m)
3.99
3 (dd)
3.83
6 (s)
1.40
7 (s)
1.37
10.73
1.01
1.01
1.05
1.07
1.04
2.11
1.04
3.04
3.11

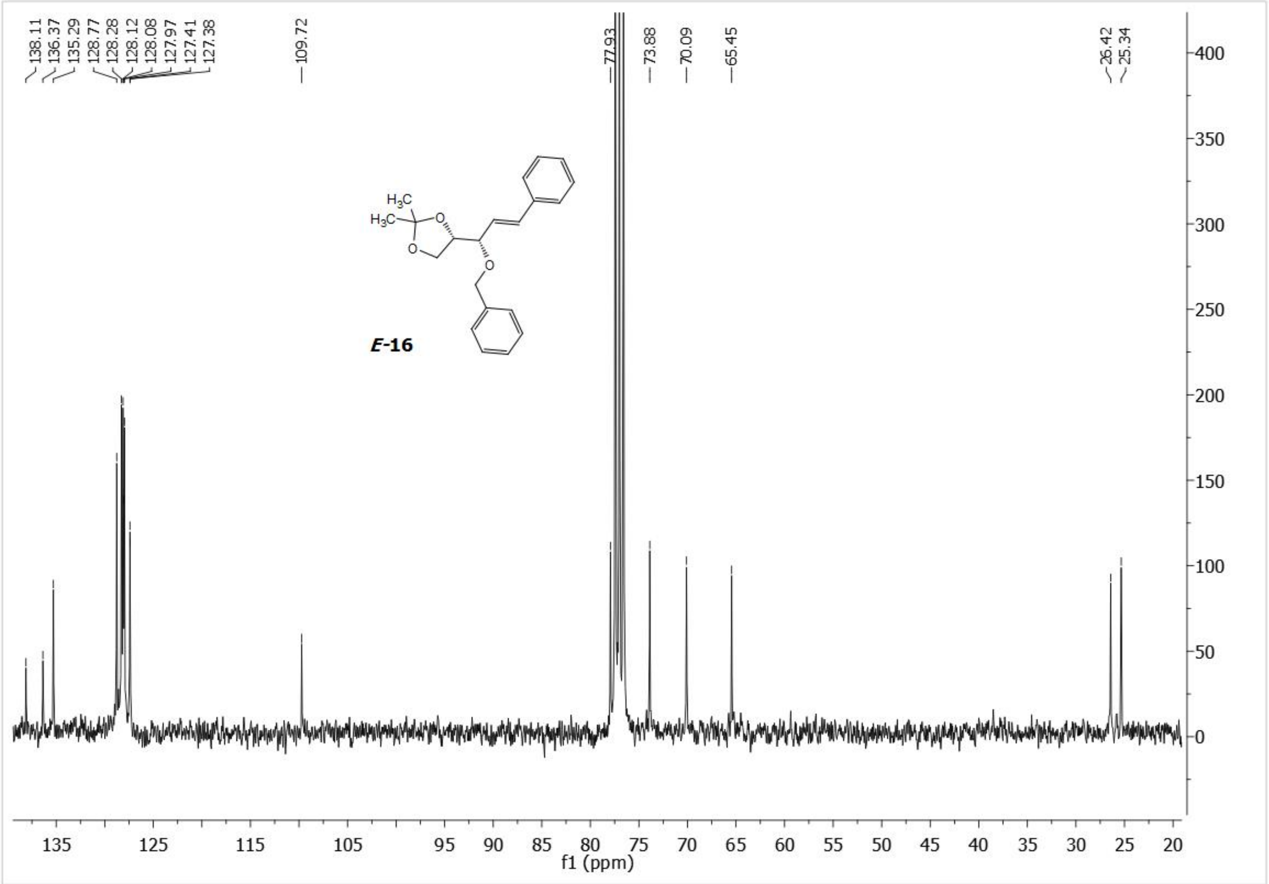

138.11
136.37
135.29
128.77
128.28
128.12
128.08
127.97
127.41
127.38
109.72
77.93
73.88
70.09
65.45
26.42
25.34
E-16
f1 (ppm)

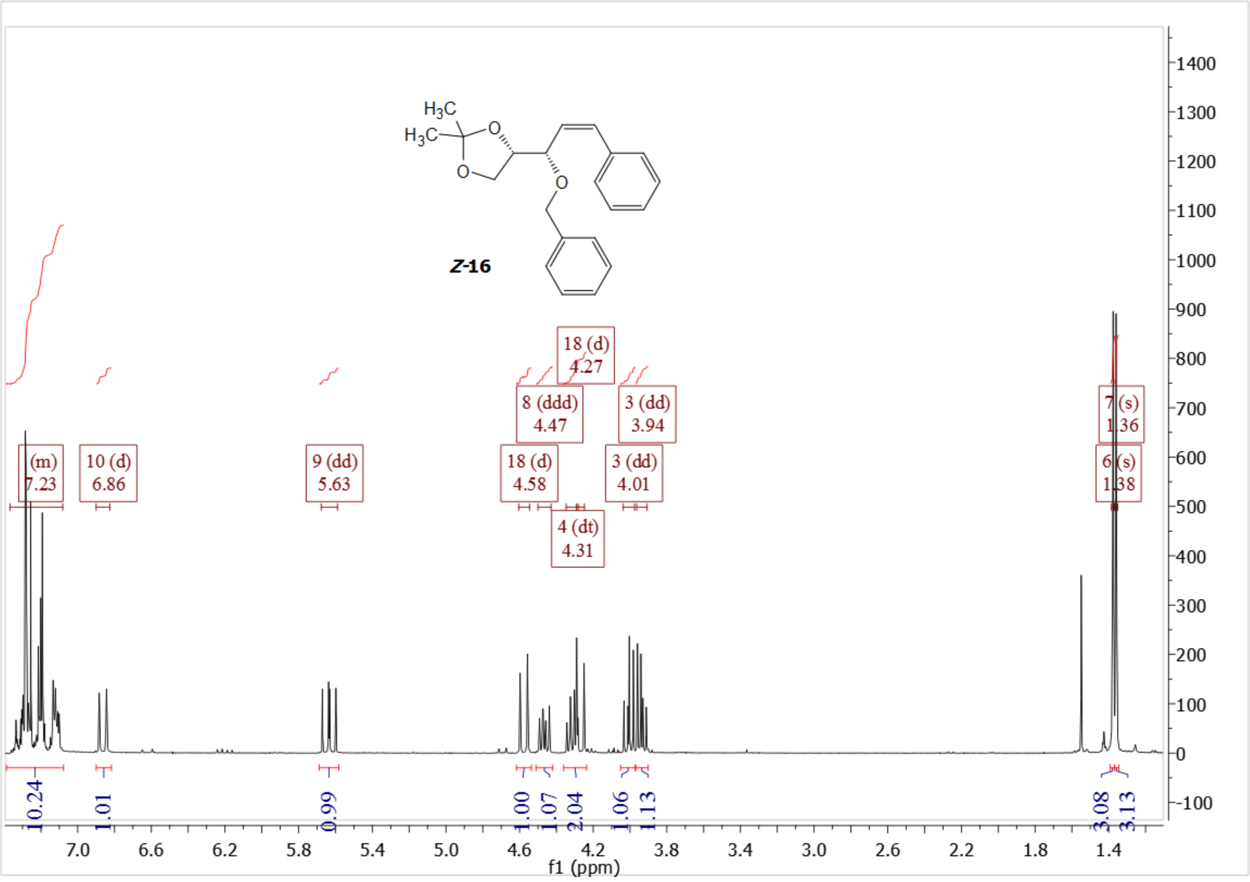
Z-16
f1 (ppm)

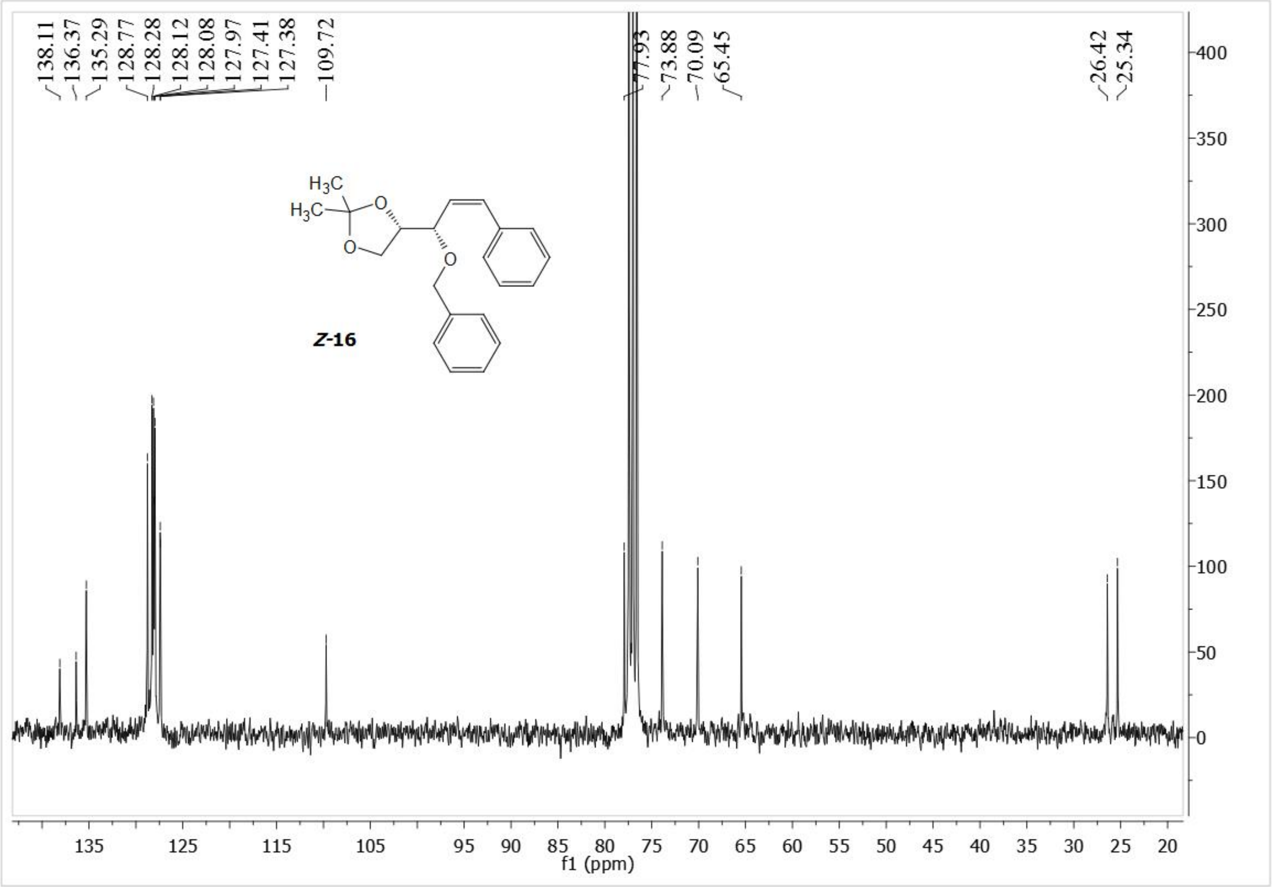
Z-16
f1 (ppm)

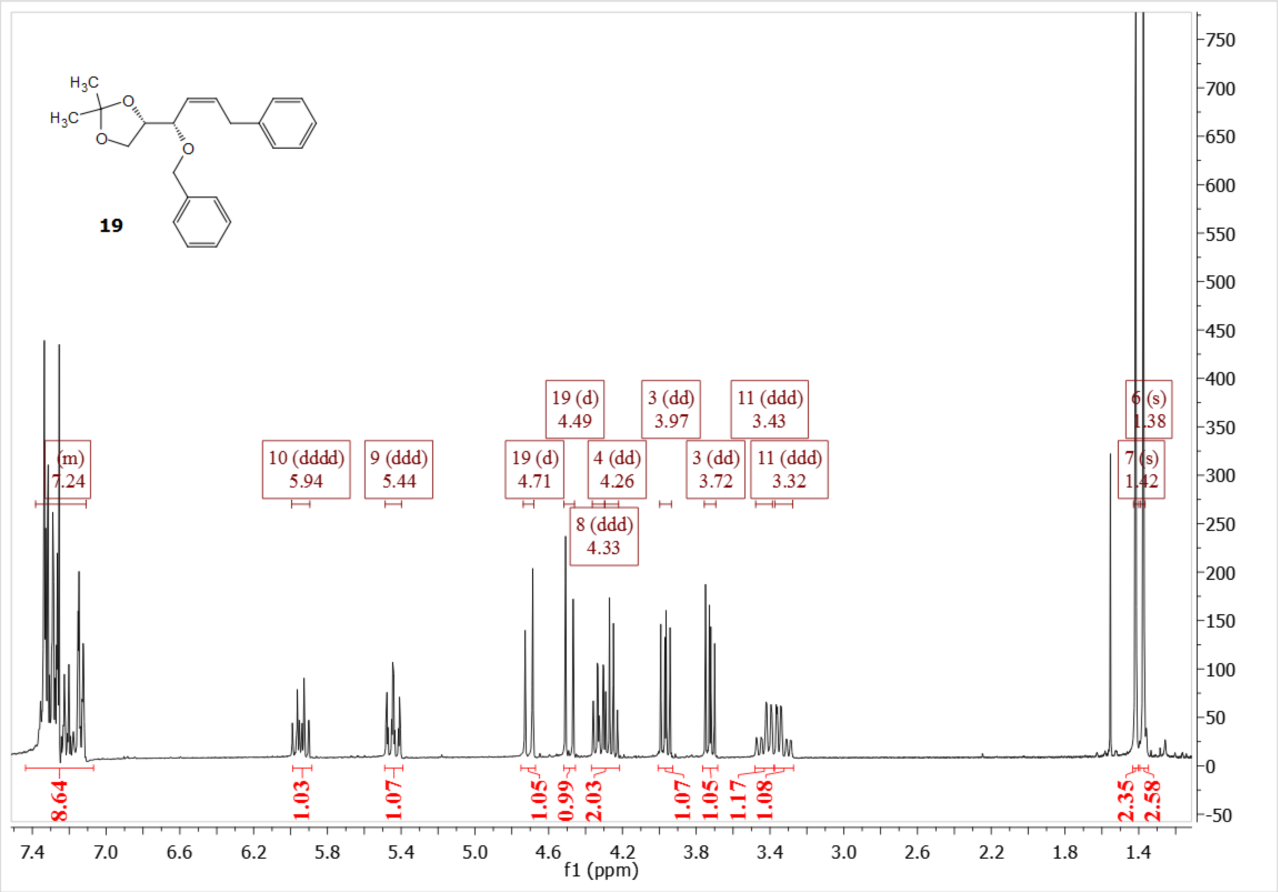

19
(m) 7.24
10 (dddd) 5.94
9 (ddd) 5.44
19 (d) 4.71
19 (d) 4.49
4 (dd) 4.26
8 (ddd) 4.33
3 (dd) 3.97
3 (dd) 3.72
11 (ddd) 3.43
11 (ddd) 3.32
6 (s) 1.38
7 (s) 1.42
8.64
1.03
1.07
1.05
0.99
2.03
1.07
1.05
1.17
1.08
2.35
2.58
f1 (ppm)

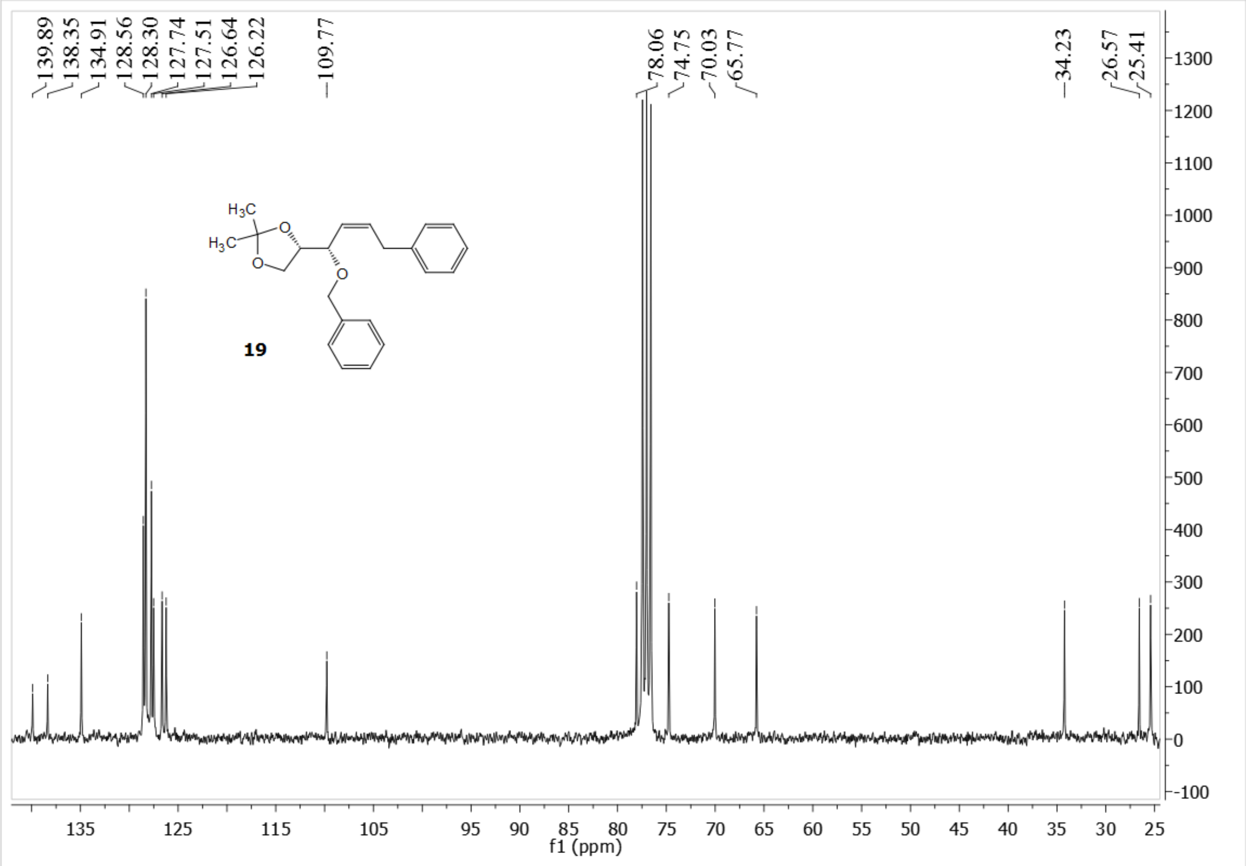

139.89
138.35
134.91
128.56
128.30
127.74
127.51
126.64
126.22
109.77
78.06
74.75
70.03
65.77
34.23
26.57
25.41
19
f1 (ppm)

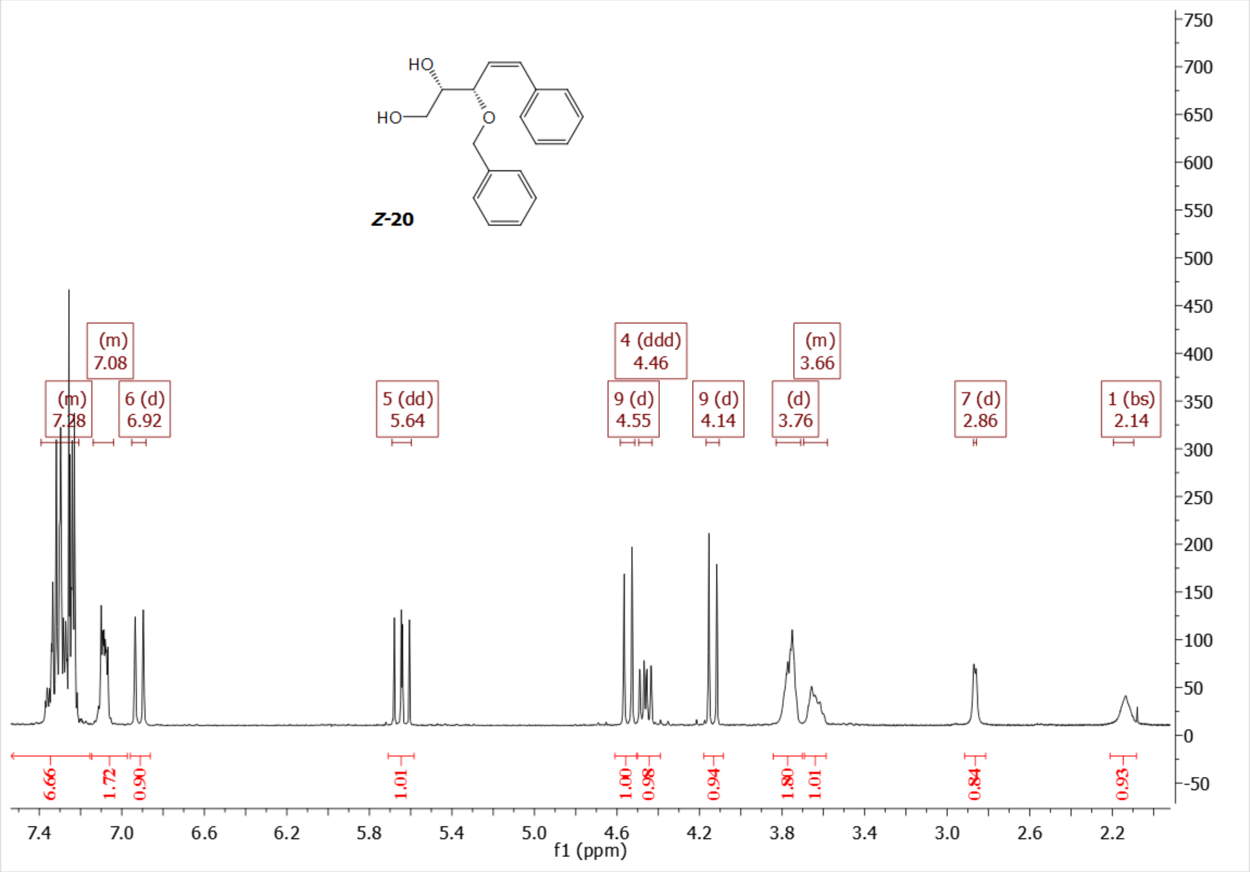
HO
HO
O
Z-20
(m)
7.28
(m)
7.08
6 (d)
6.92
5 (dd)
5.64
9 (d)
4.55
4 (ddd)
4.46
9 (d)
4.14
(d)
3.76
(m)
3.66
7 (d)
2.86
1 (bs)
2.14
6.66
1.72
0.90
1.01
1.00
0.98
0.94
1.80
1.01
0.84
0.93
f1 (ppm)

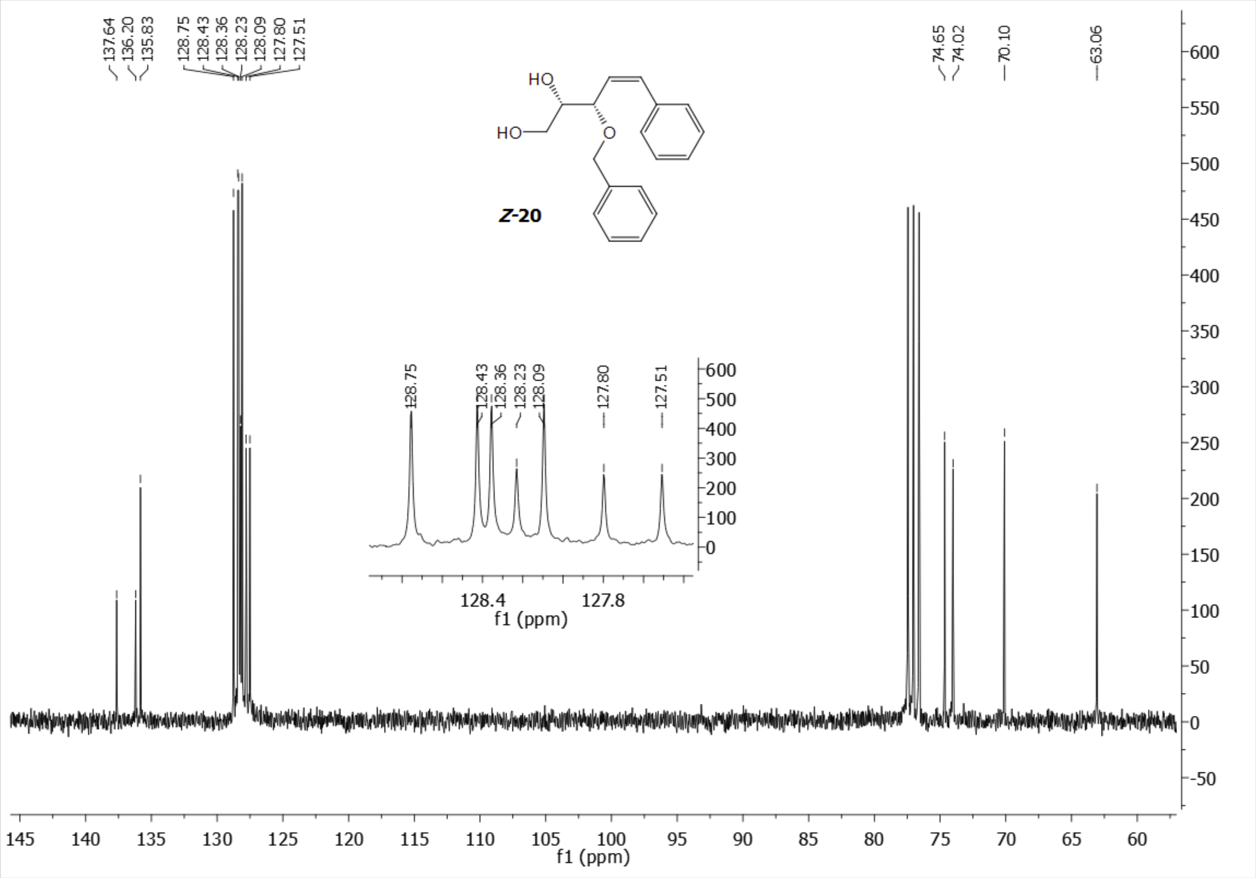
137.64
136.20
135.83
128.75
128.43
128.36
128.23
128.09
127.80
127.51
74.65
74.02
70.10
63.06
HO
HO
O
Z-20
f1 (ppm)

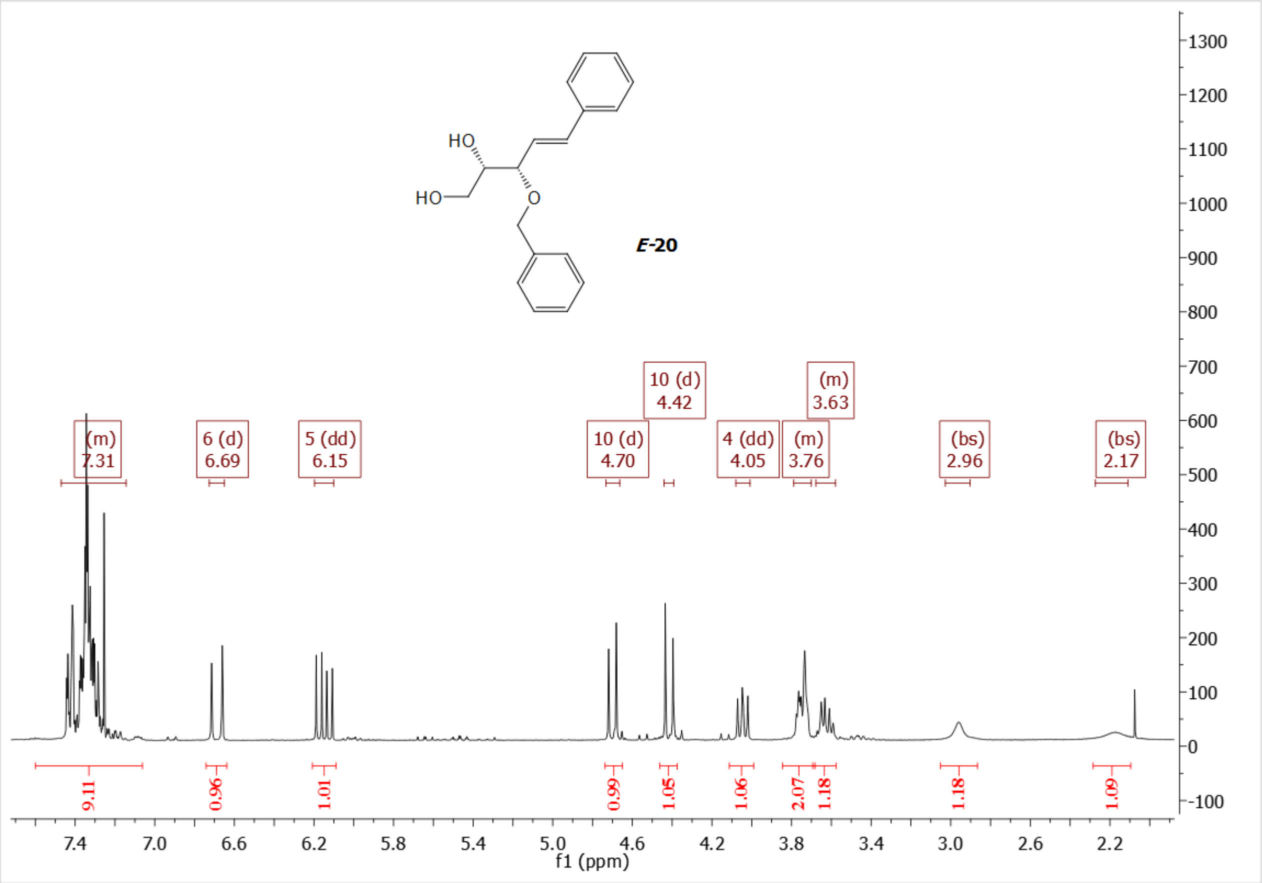
HO
HO
O
E-20
(m)
7.31
6 (d)
6.69
5 (dd)
6.15
10 (d)
4.70
10 (d)
4.42
4 (dd)
4.05
(m)
3.76
(m)
3.63
(bs)
2.96
(bs)
2.17
9.11
0.96
1.01
0.99
1.05
1.06
2.07
1.18
1.18
1.09
f1 (ppm)

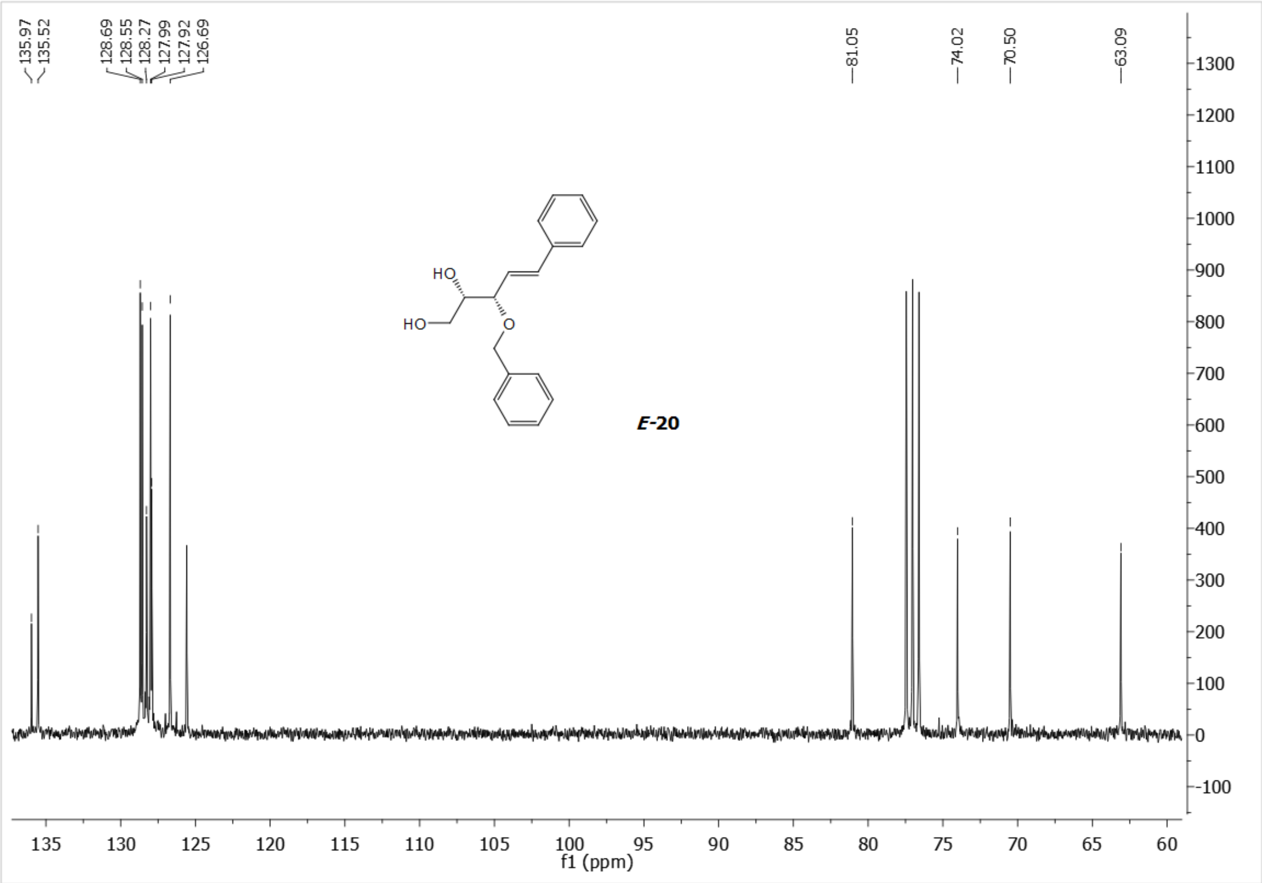
135.97
135.52
128.69
128.55
128.27
127.99
127.92
126.69
81.05
74.02
70.50
63.09
HO
HO
O
E-20
f1 (ppm)

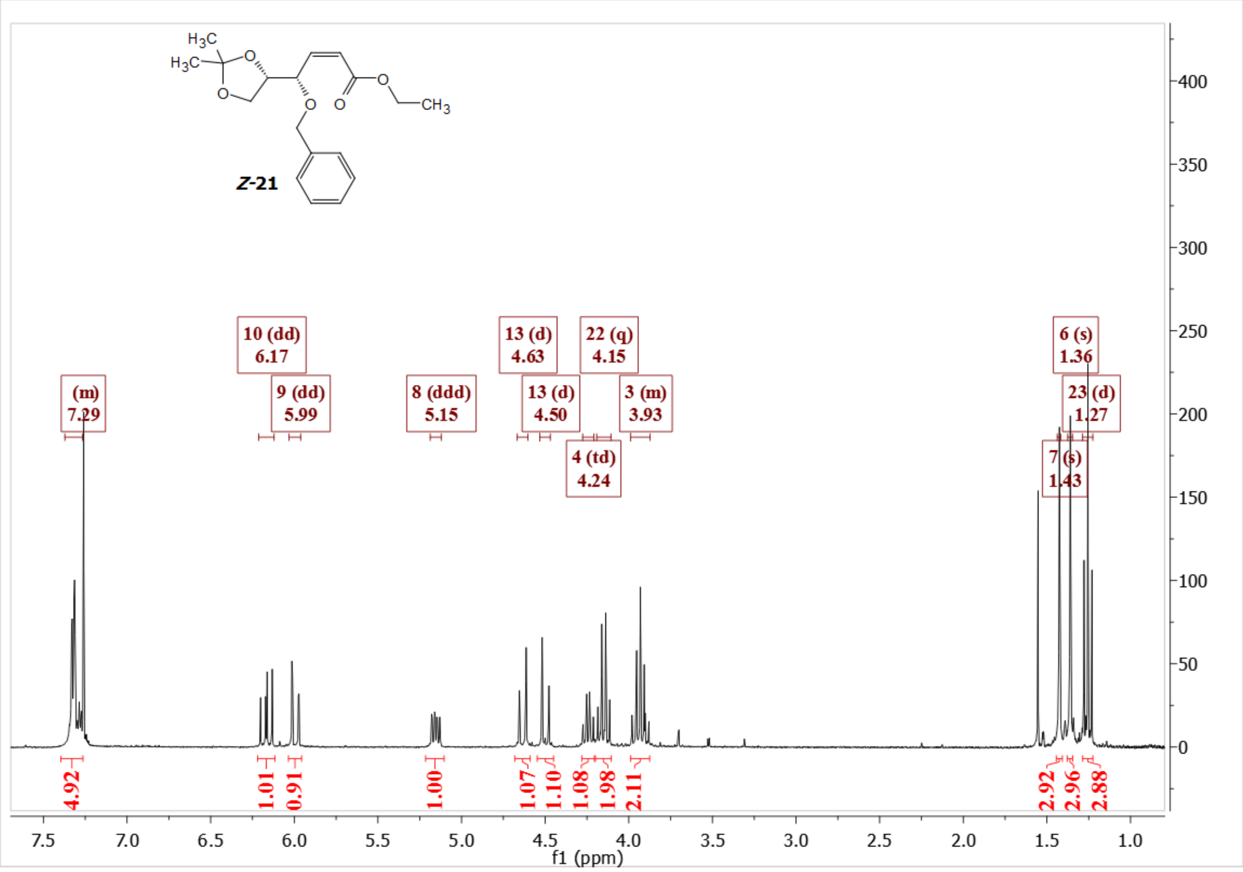
Z-21
10 (dd) 6.17
(m) 7.29
9 (dd) 5.99
8 (ddd) 5.15
13 (dd) 4.63
13 (d) 4.50
22 (q) 4.15
4 (td) 4.24
3 (m) 3.93
6 (s) 1.36
23 (d) 1.27
7 (s) 1.43
4.92
1.01
0.91
1.00
1.07
1.10
1.08
1.98
2.11
2.92
2.96
2.88
f1 (ppm)

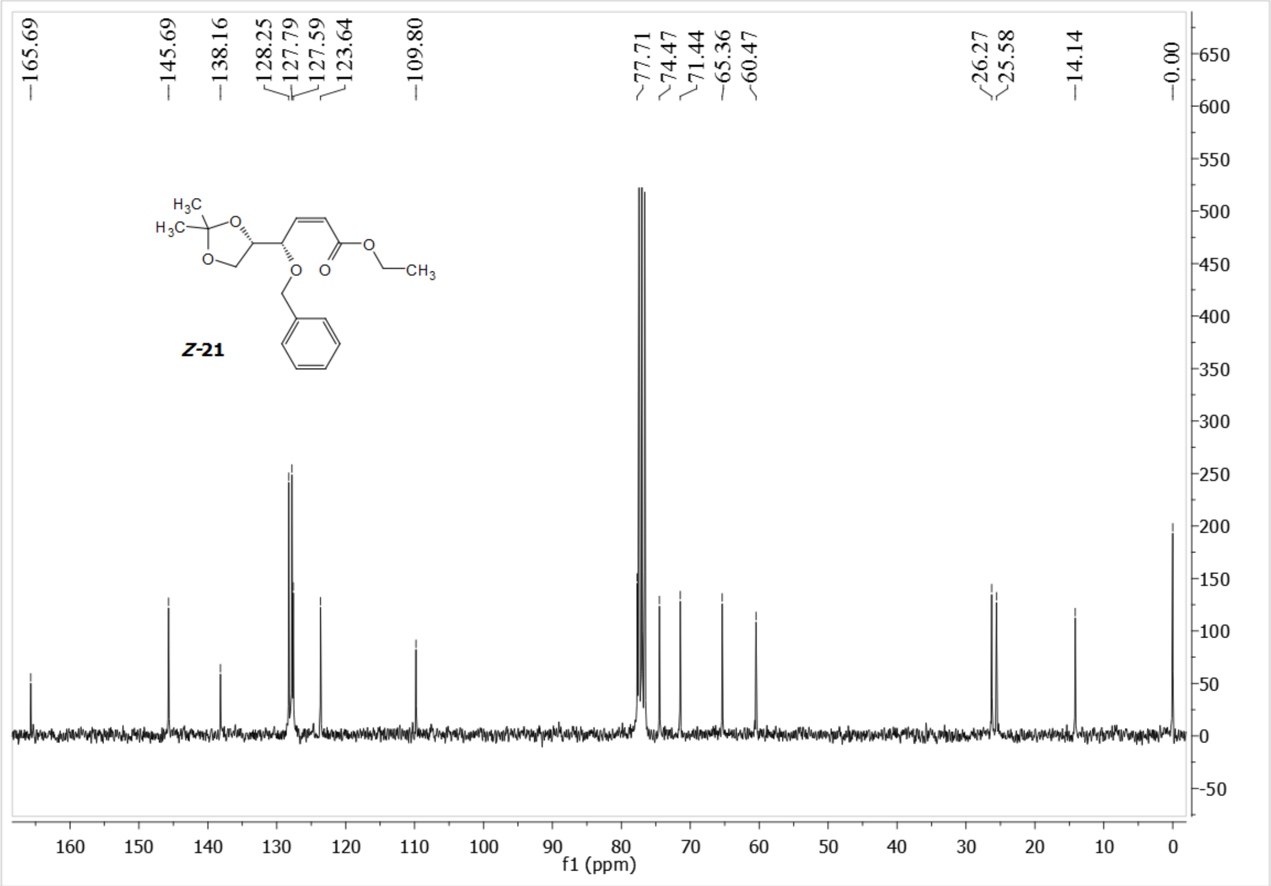
165.69
145.69
138.16
128.25
127.79
127.59
123.64
109.80
77.71
74.47
71.44
65.36
60.47
26.27
25.58
14.14
0.00
Z-21
f1 (ppm)

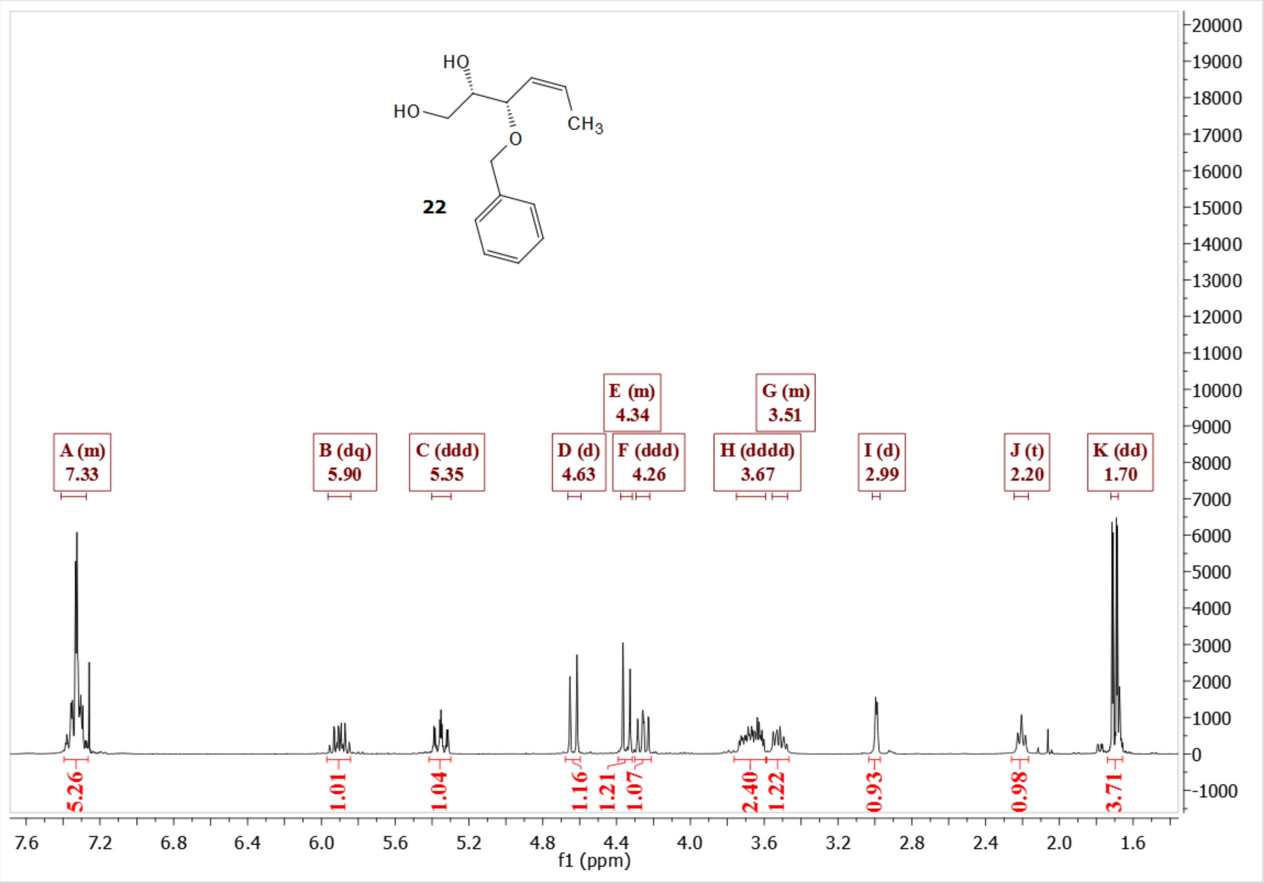
HO
HO
CH3
O
22
A (m)
7.33
B (dq)
5.90
C (ddd)
5.35
D (d)
4.63
E (m)
4.34
F (ddd)
4.26
H (dddd)
3.67
G (m)
3.51
I (d)
2.99
J (t)
2.20
K (dd)
1.70
5.26
1.01
1.04
1.16
1.21
1.07
2.40
1.22
0.93
0.98
3.71
f1 (ppm)

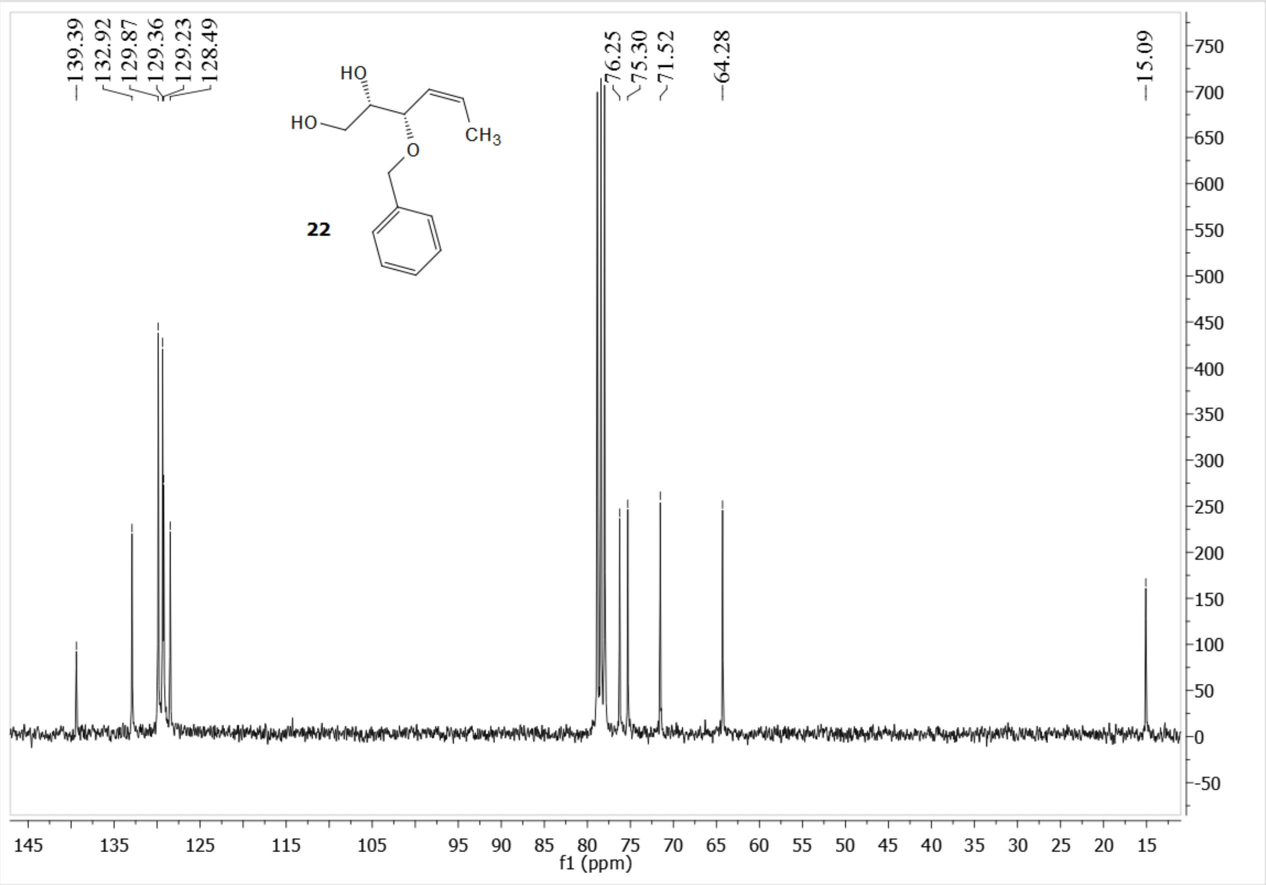
139.39
132.92
129.87
129.36
129.23
128.49
76.25
75.30
71.52
64.28
15.09
HO
HO
CH3
O
22
f1 (ppm)

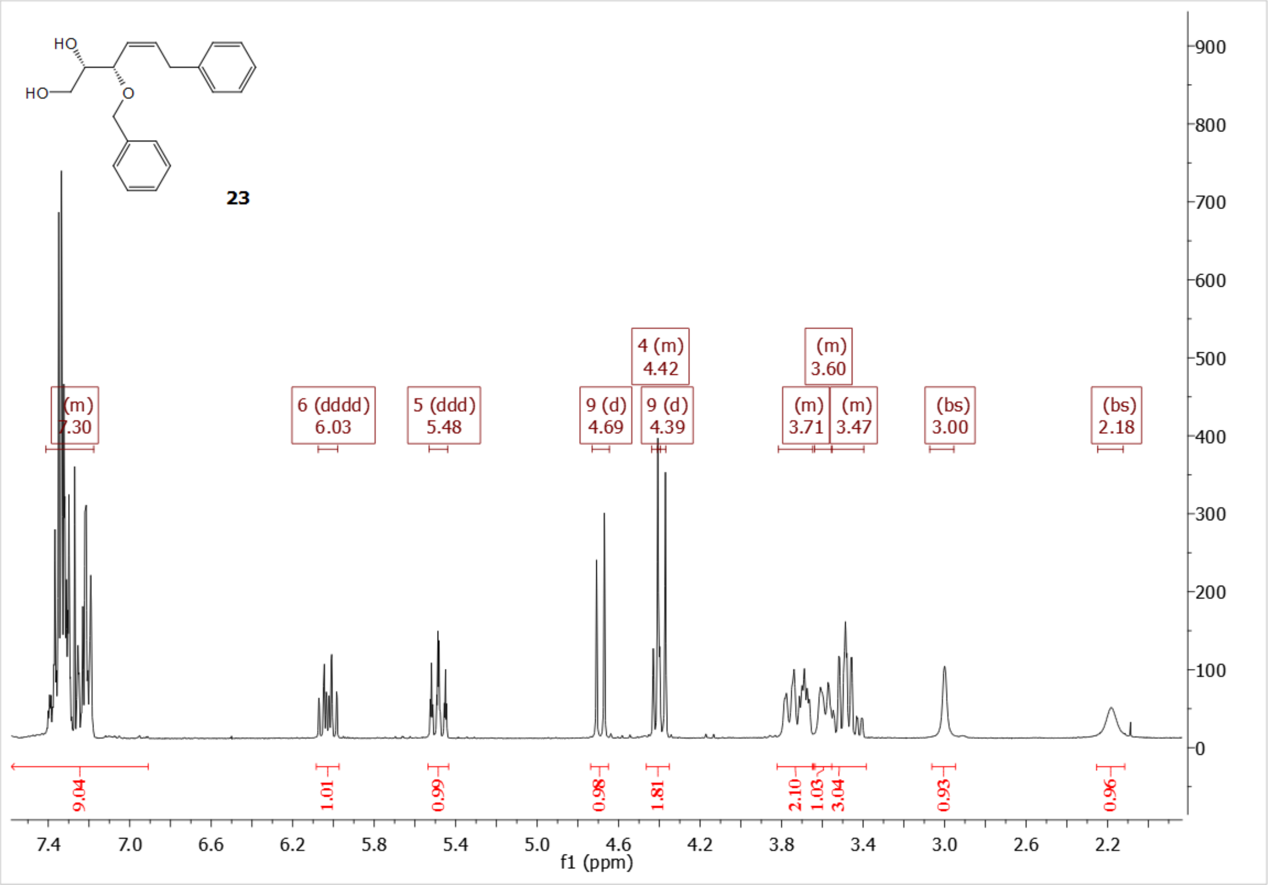

HO
HO
O
23
(m)
7.30
6 (dddd)
6.03
5 (ddd)
5.48
9 (d)
4.69
4 (m)
4.42
9 (d)
4.39
(m)
3.71
(m)
3.60
(m)
3.47
(bs)
3.00
(bs)
2.18
9.04
1.01
0.99
0.98
1.81
2.10
1.03
3.04
0.93
0.96
f1 (ppm)

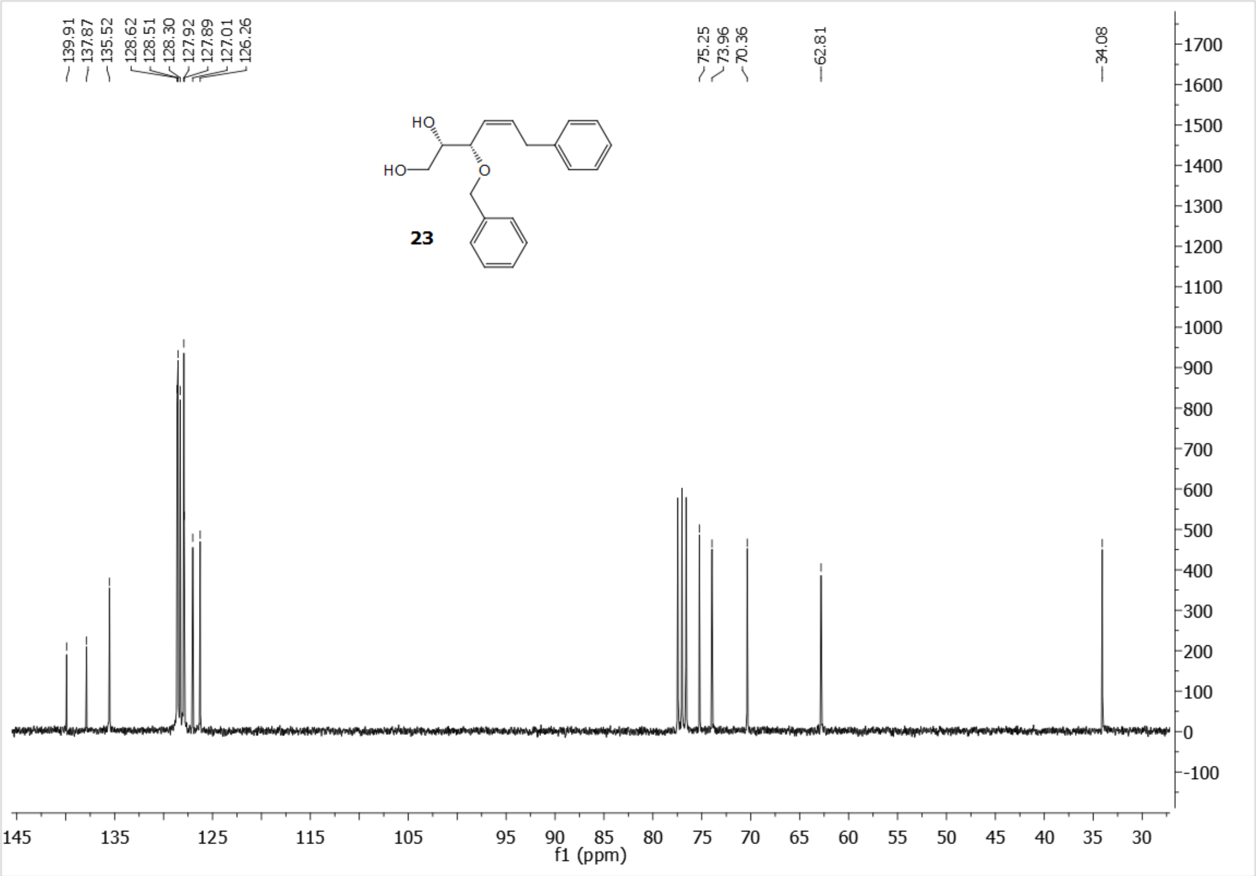

139.91
137.87
135.52
128.62
128.51
128.30
127.92
127.89
127.01
126.26
75.25
73.96
70.36
62.81
34.08
HO
HO
O
23
f1 (ppm)

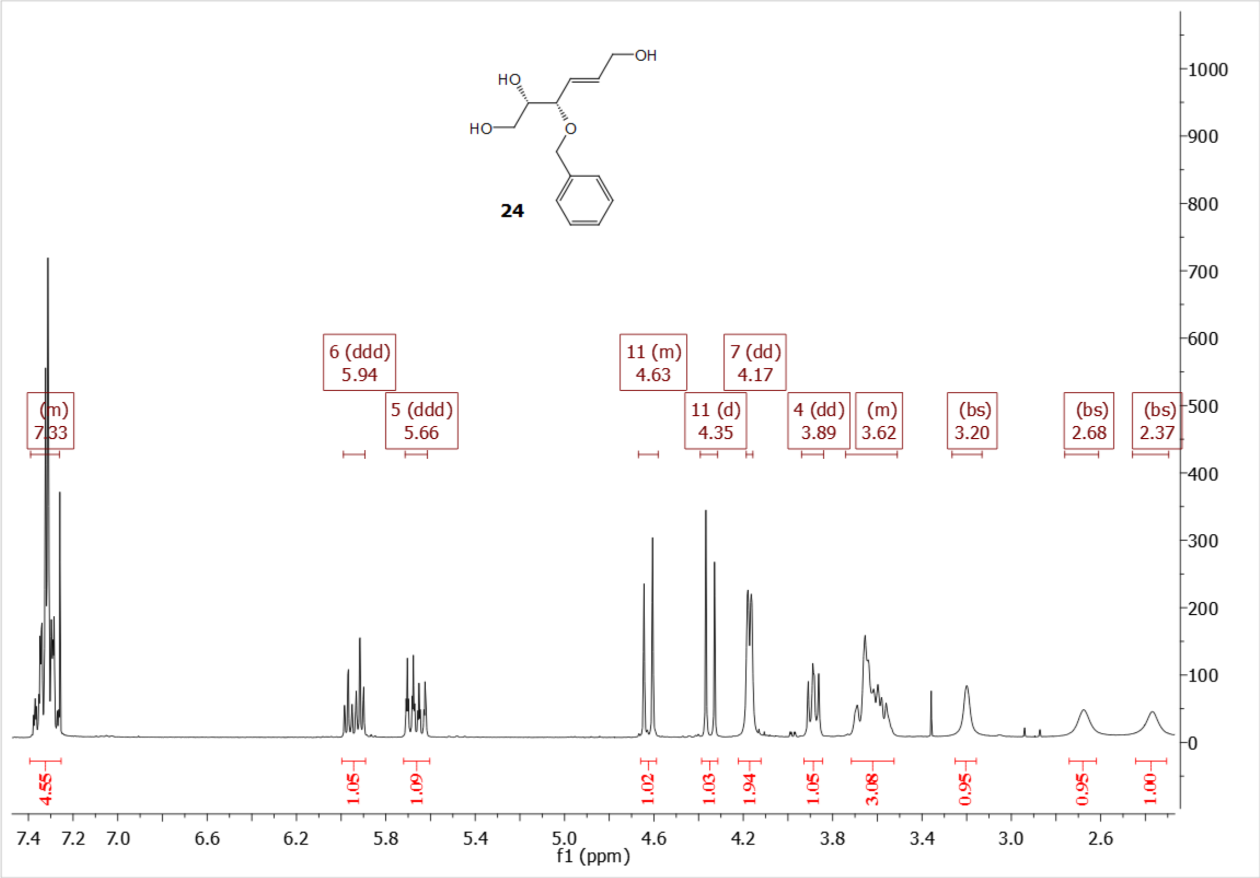

OH
HO
HO
O
24
(m)
7.33
6 (ddd)
5.94
5 (ddd)
5.66
11 (m)
4.63
11 (d)
4.35
7 (dd)
4.17
4 (dd)
3.89
(m)
3.62
(bs)
3.20
(bs)
2.68
(bs)
2.37
4.55
1.05
1.09
1.02
1.03
1.94
1.05
3.08
0.95
0.95
1.00
f1 (ppm)

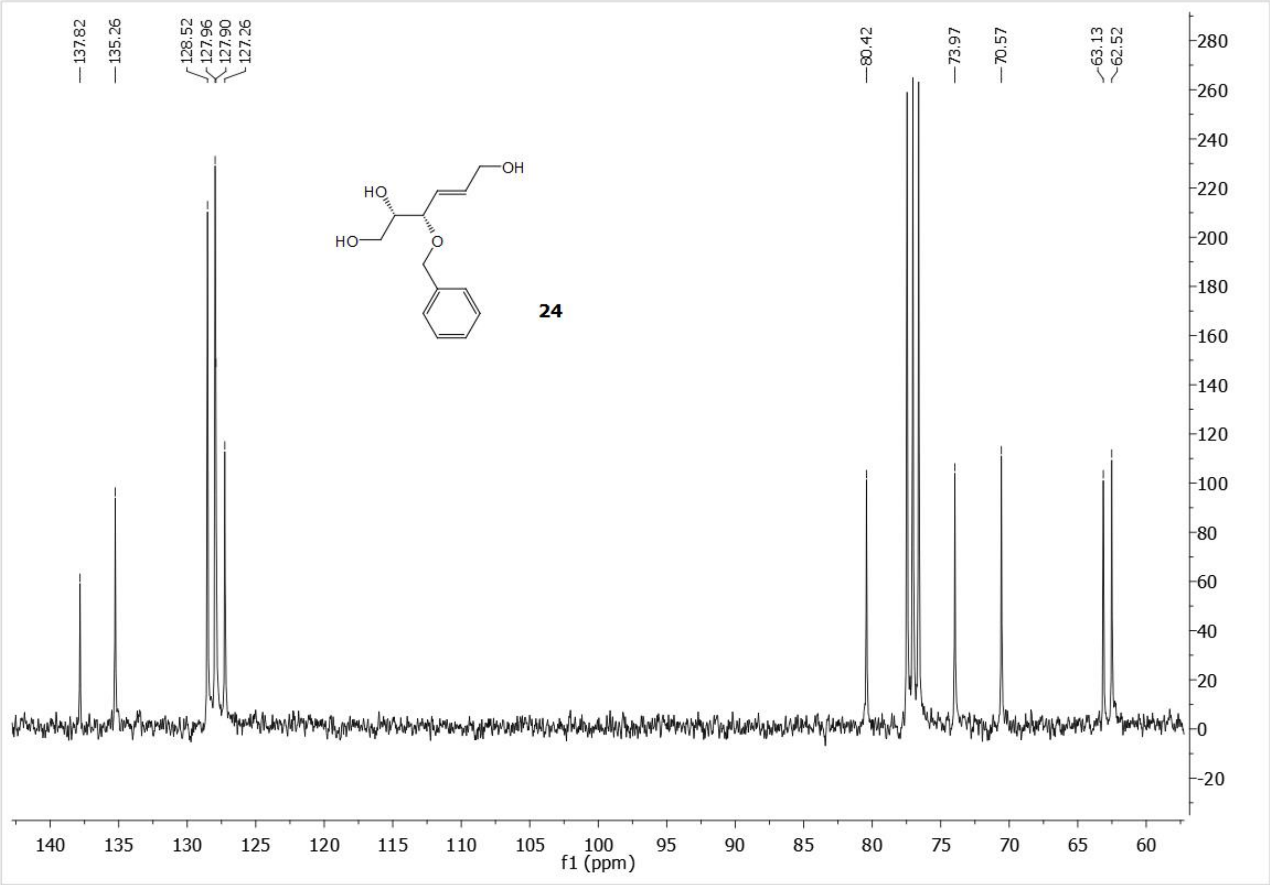

137.82
135.26
128.52
127.96
127.90
127.26
80.42
73.97
70.57
63.13
62.52
OH
HO
HO
O
24
f1 (ppm)

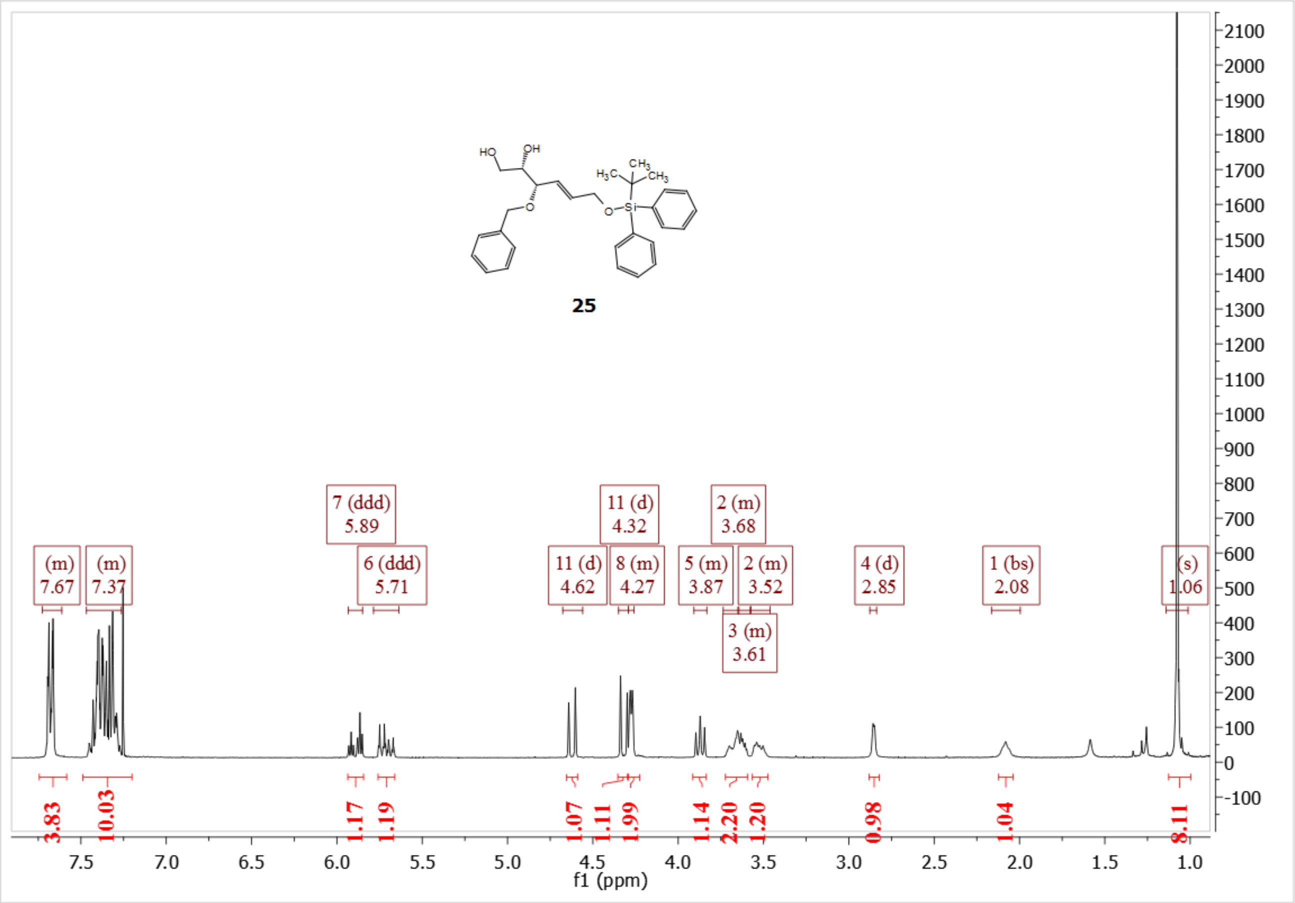
25
7 (ddd) 5.89
11 (d) 4.32
2 (m) 3.68
(m) 7.67
(m) 7.37
6 (ddd) 5.71
11 (d) 4.62
8 (m) 4.27
5 (m) 3.87
2 (m) 3.52
3 (m) 3.61
4 (d) 2.85
1 (bs) 2.08
(s) 1.06
3.83
10.03
1.17
1.19
1.07
1.11
1.99
1.14
2.20
1.20
0.98
1.04
8.11
f1 (ppm)

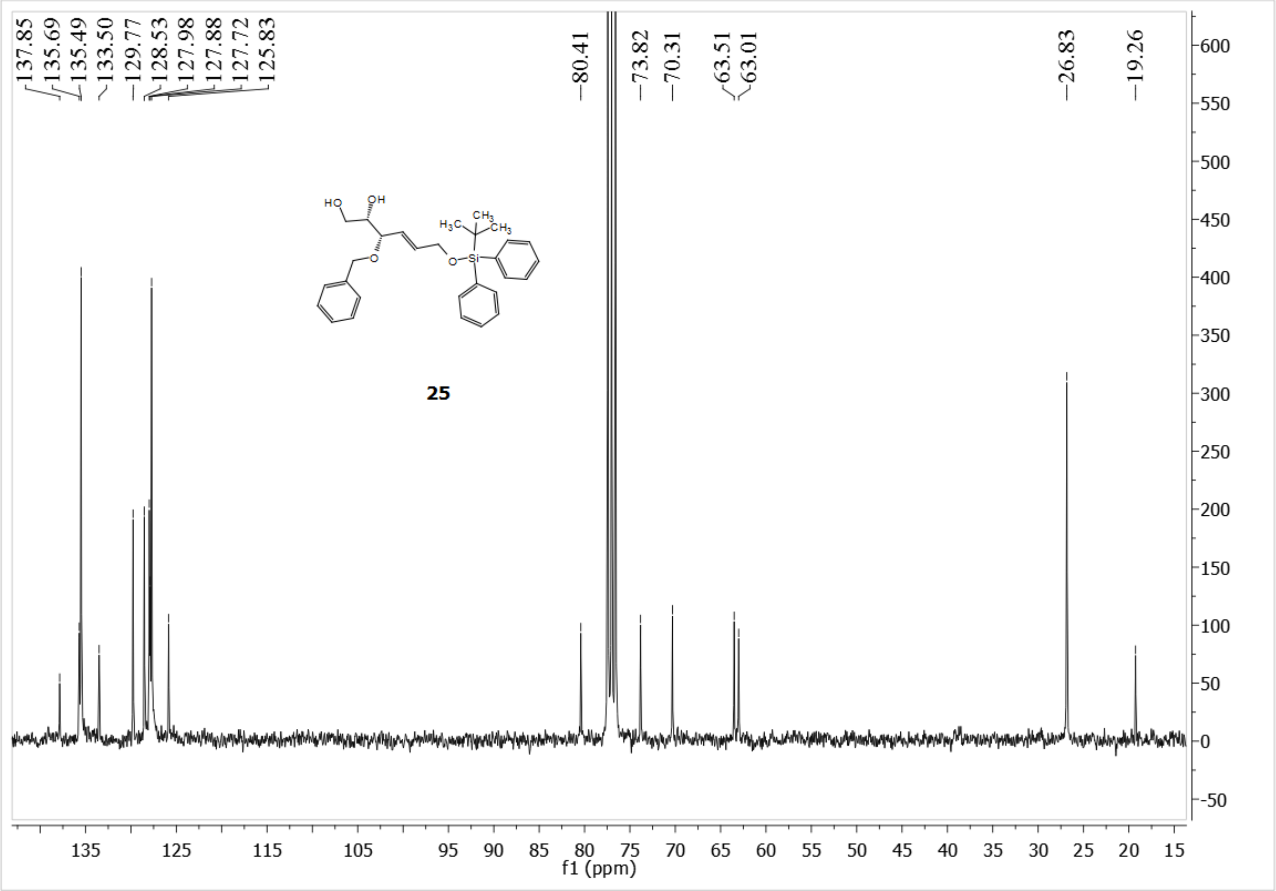
137.85
135.69
135.49
133.50
129.77
128.53
127.98
127.88
127.72
125.83
80.41
73.82
70.31
63.51
63.01
26.83
19.26
25
f1 (ppm)

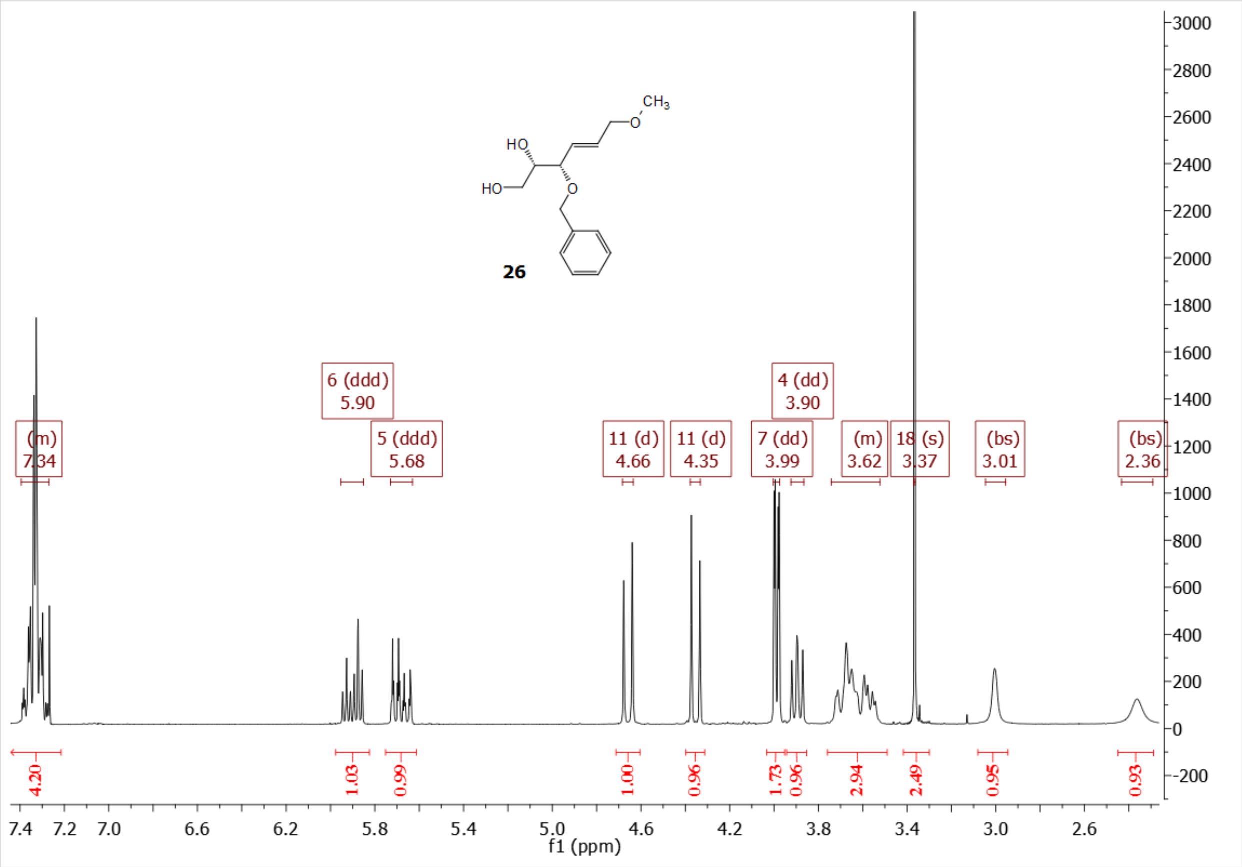

CH3
HO
HO
O
O
26
(m)
7.34
6 (ddd)
5.90
5 (ddd)
5.68
11 (d)
4.66
11 (d)
4.35
7 (dd)
3.99
4 (dd)
3.90
(m)
3.62
18 (s)
3.37
(bs)
3.01
(bs)
2.36
4.20
1.03
0.99
1.00
0.96
1.73
0.96
2.94
2.49
0.95
0.93
f1 (ppm)

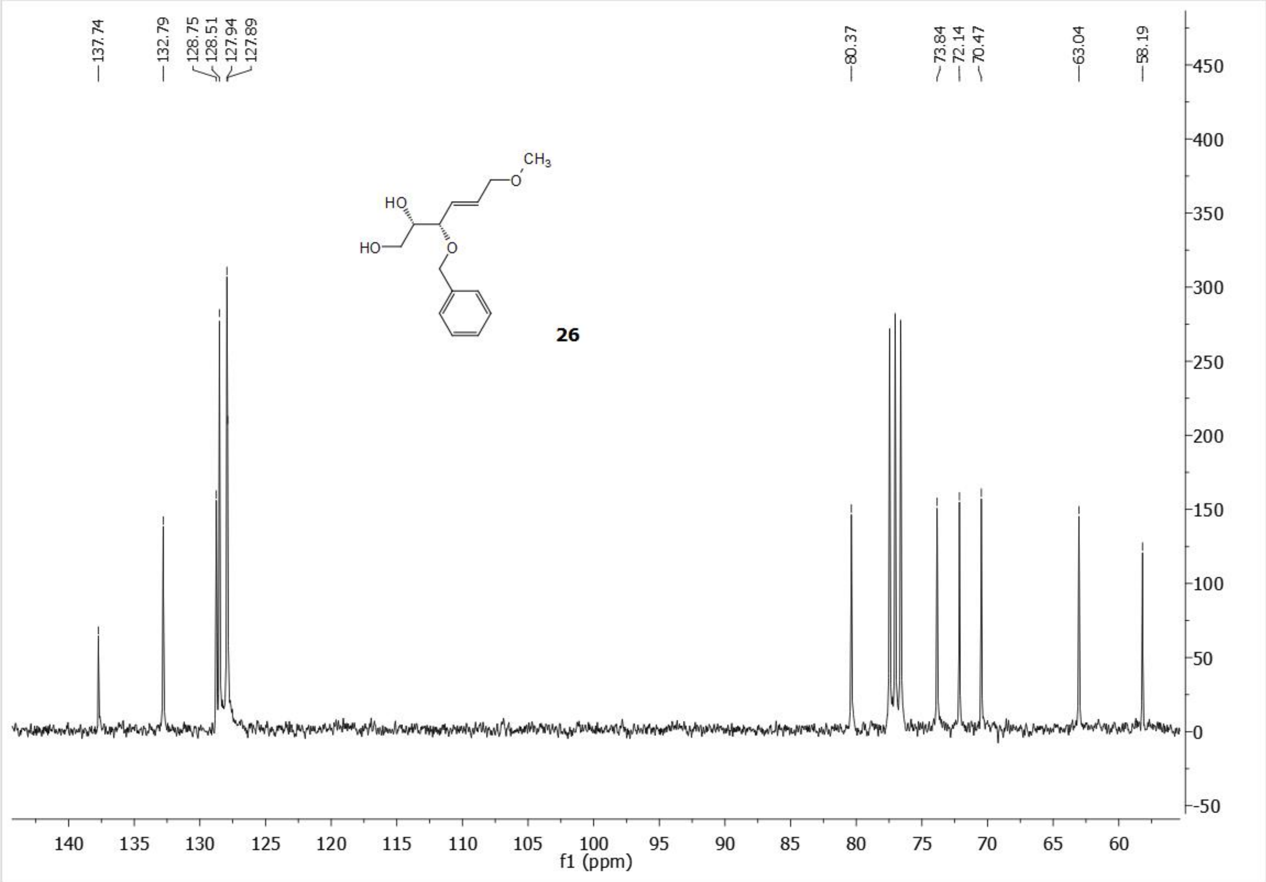

137.74
132.79
128.75
128.51
127.94
127.89
80.37
73.84
72.14
70.47
63.04
58.19
CH3
HO
HO
O
O
26
f1 (ppm)

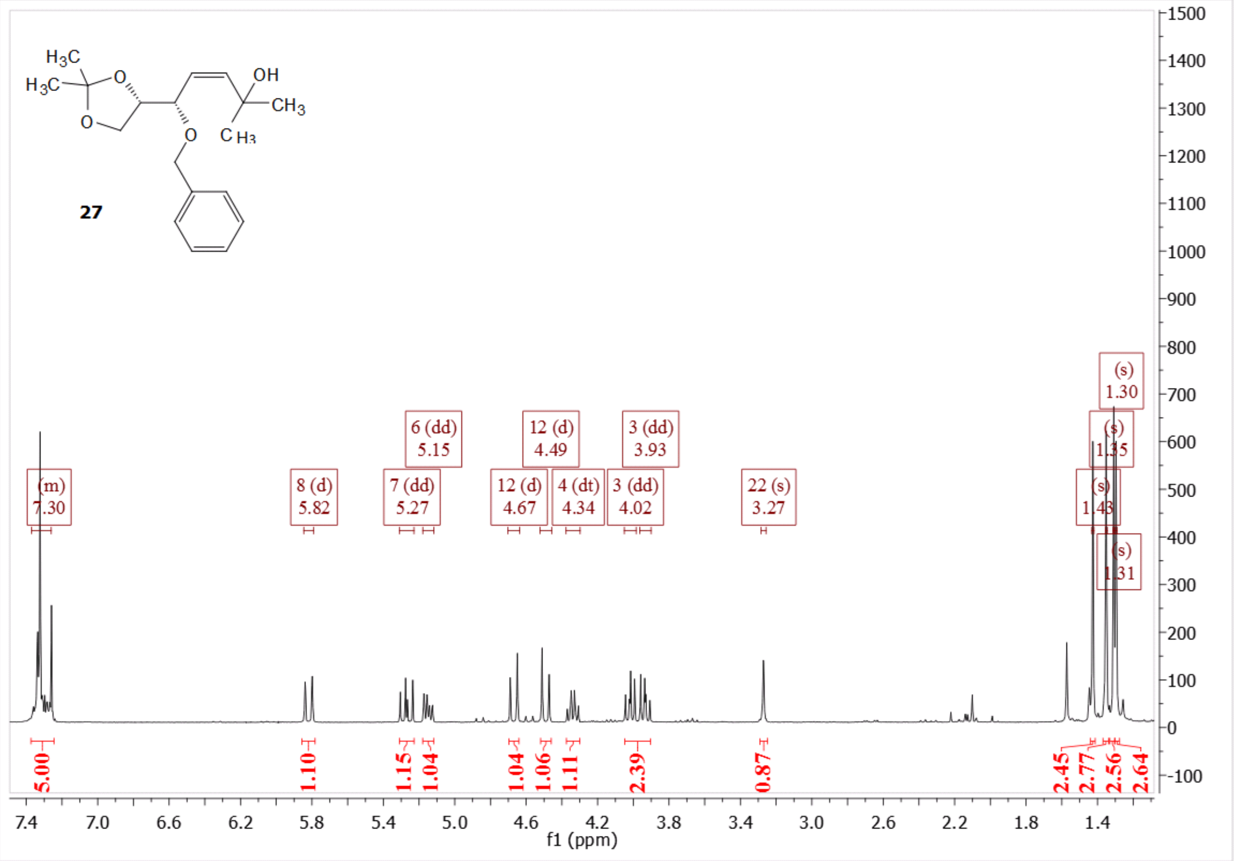
27
(m) 7.30
8 (d) 5.82
7 (dd) 5.27
6 (dd) 5.15
12 (d) 4.67
12 (d) 4.49
4 (dt) 4.34
3 (dd) 4.02
3 (dd) 3.93
22 (s) 3.27
(s) 1.43
(s) 1.35
(s) 1.31
(s) 1.30
5.00
1.10
1.15
1.04
1.04
1.06
1.11
2.39
0.87
2.45
2.77
2.56
2.64
f1 (ppm)

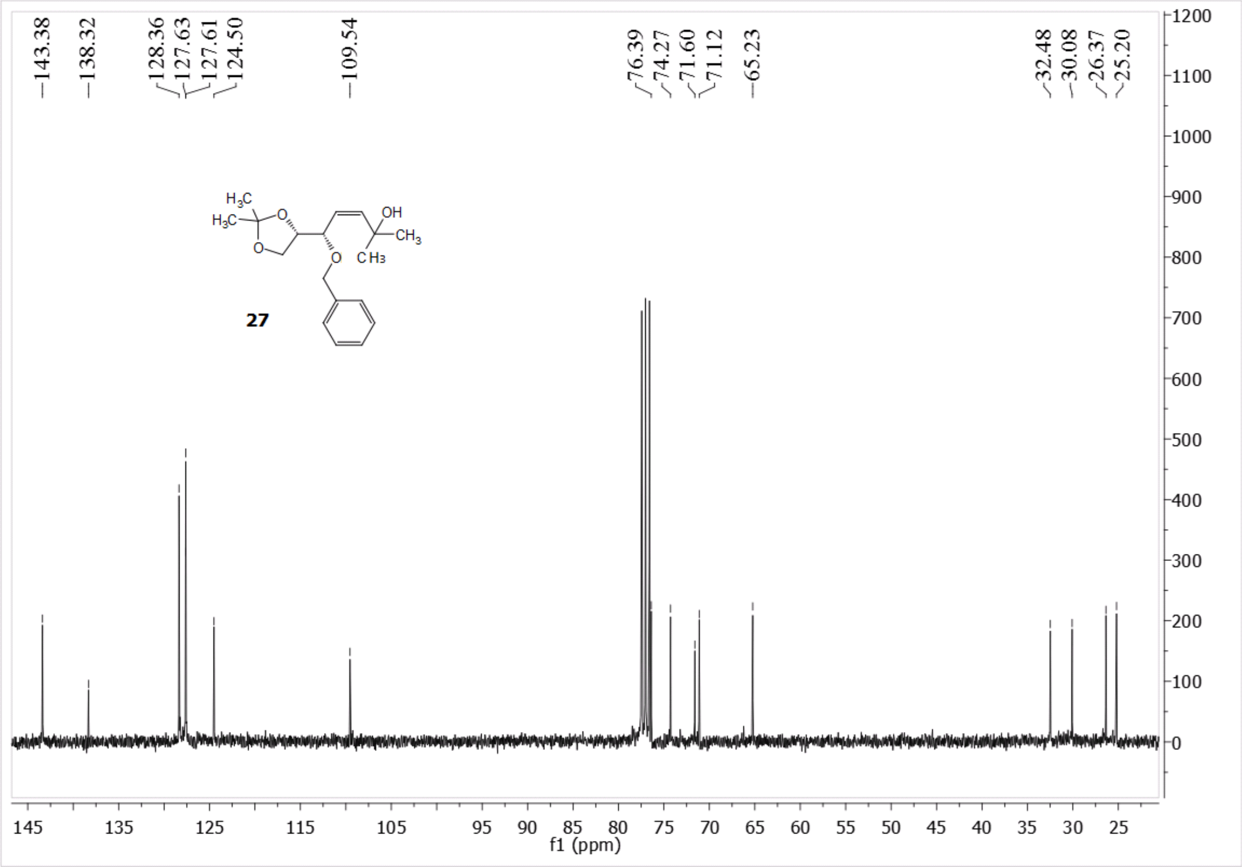
143.38
138.32
128.36
127.63
127.61
124.50
109.54
76.39
74.27
71.60
71.12
65.23
32.48
30.08
26.37
25.20
27
f1 (ppm)

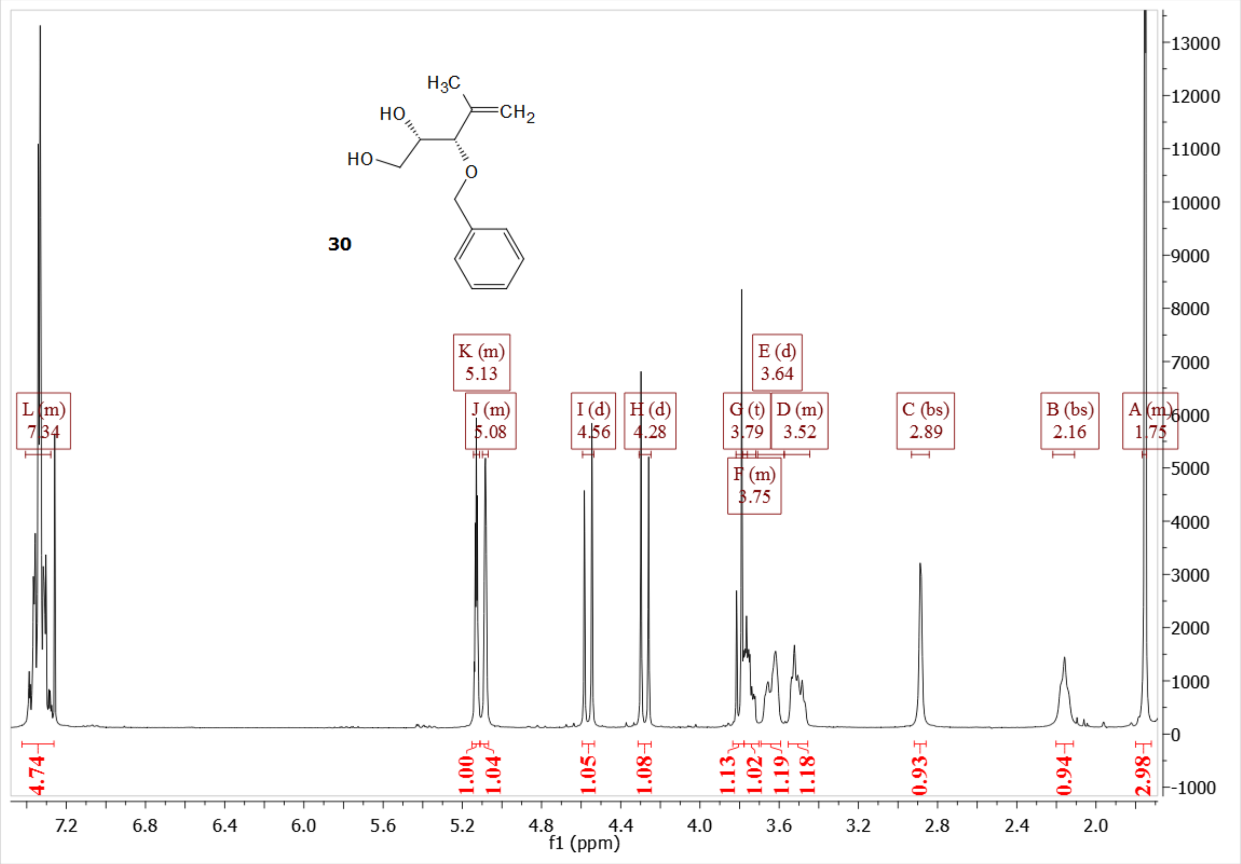
30
L (m) 7.34
K (m) 5.13
J (m) 5.08
I (d) 4.56
H (d) 4.28
G (t) 3.79
E (d) 3.64
F (m) 3.75
D (m) 3.52
C (bs) 2.89
B (bs) 2.16
A (m) 1.75
4.74
1.00
1.04
1.05
1.08
1.13
1.02
1.19
1.18
0.93
0.94
2.98
f1 (ppm)

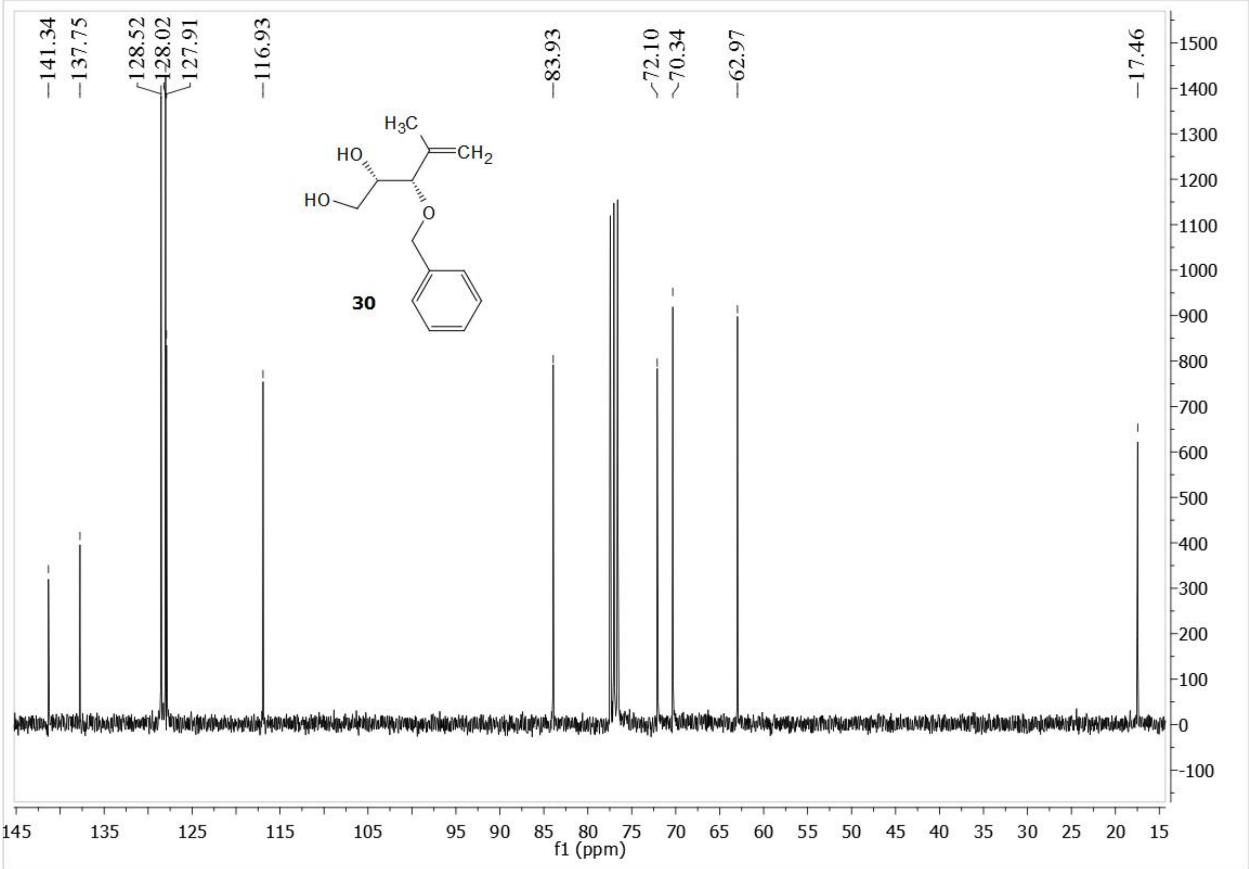
141.34
137.75
128.52
128.02
127.91
116.93
83.93
72.10
70.34
62.97
17.46
30
f1 (ppm)

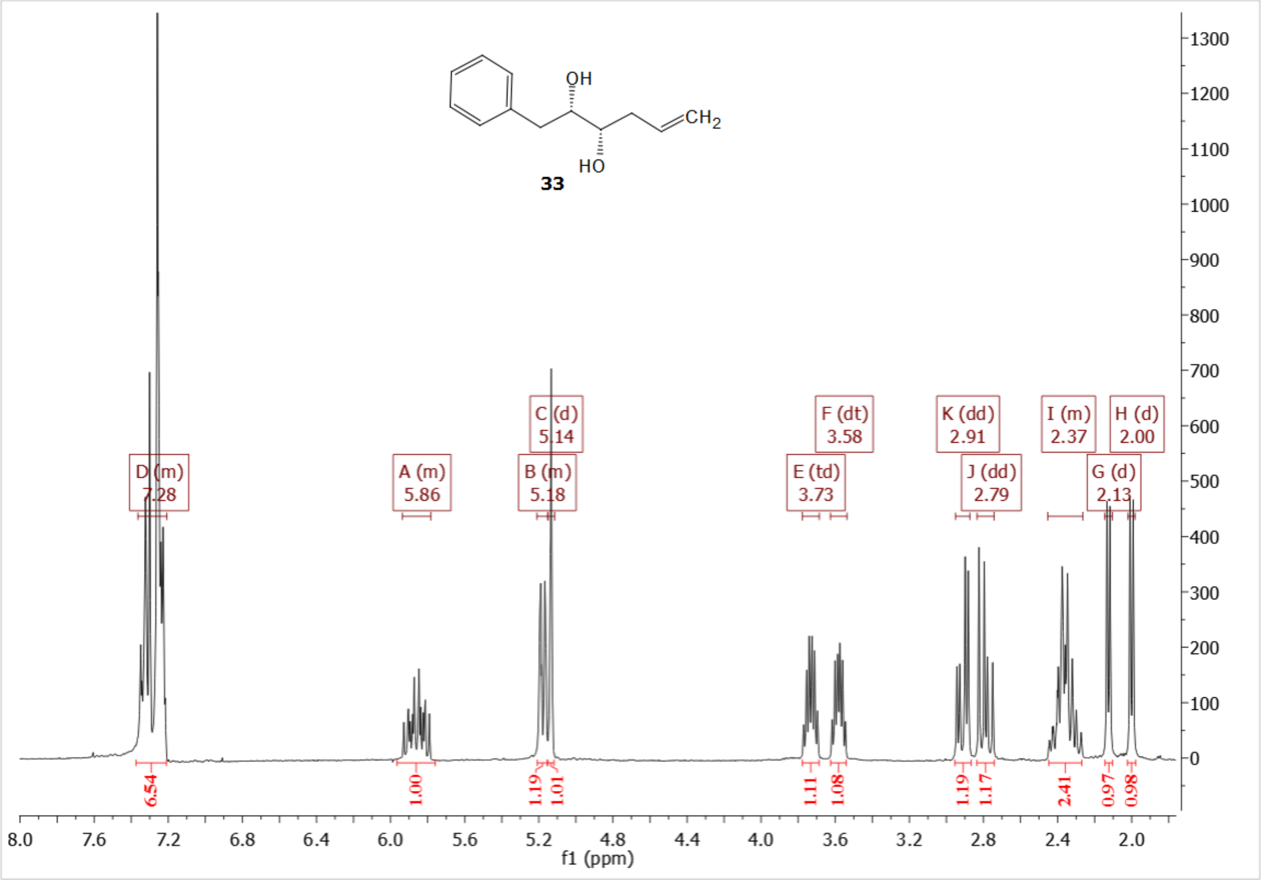

OH
CH2
HO
33
D (m) 7.28
A (m) 5.86
B (m) 5.18
C (d) 5.14
E (td) 3.73
F (dt) 3.58
K (dd) 2.91
J (dd) 2.79
I (m) 2.37
G (d) 2.13
H (d) 2.00
6.54
1.00
1.19
1.01
1.11
1.08
1.19
1.17
2.41
0.97
0.98
f1 (ppm)

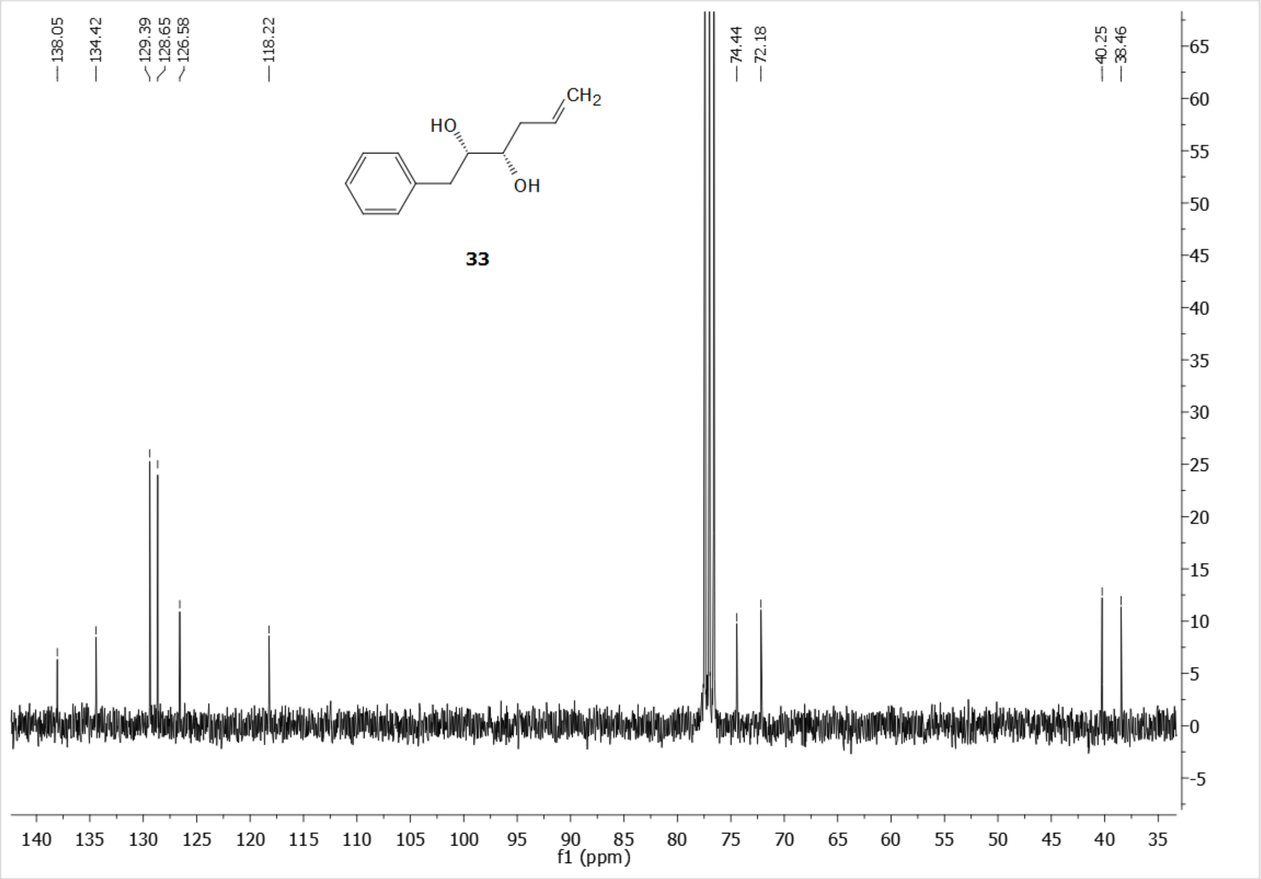

138.05
134.42
129.39
128.65
126.58
118.22
74.44
72.18
40.25
38.46
HO
CH2
OH
33
f1 (ppm)

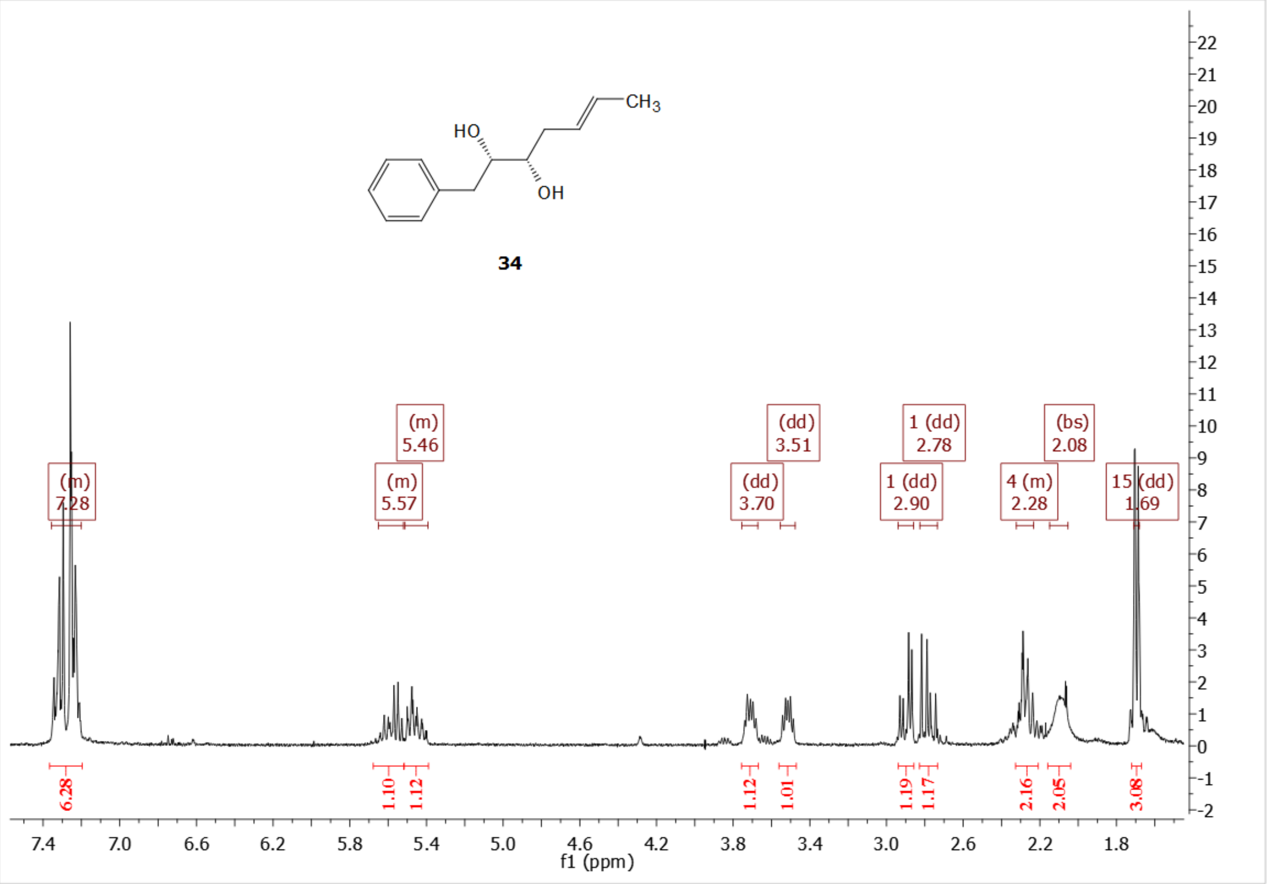
HO
OH
CH3
34
(m)
7.28
(m)
5.57
(m)
5.46
(dd)
3.70
(dd)
3.51
1 (dd)
2.90
1 (dd)
2.78
4 (m)
2.28
(bs)
2.08
15 (dd)
1.69
6.28
1.10
1.12
1.12
1.01
1.19
1.17
2.16
2.05
3.08
f1 (ppm)

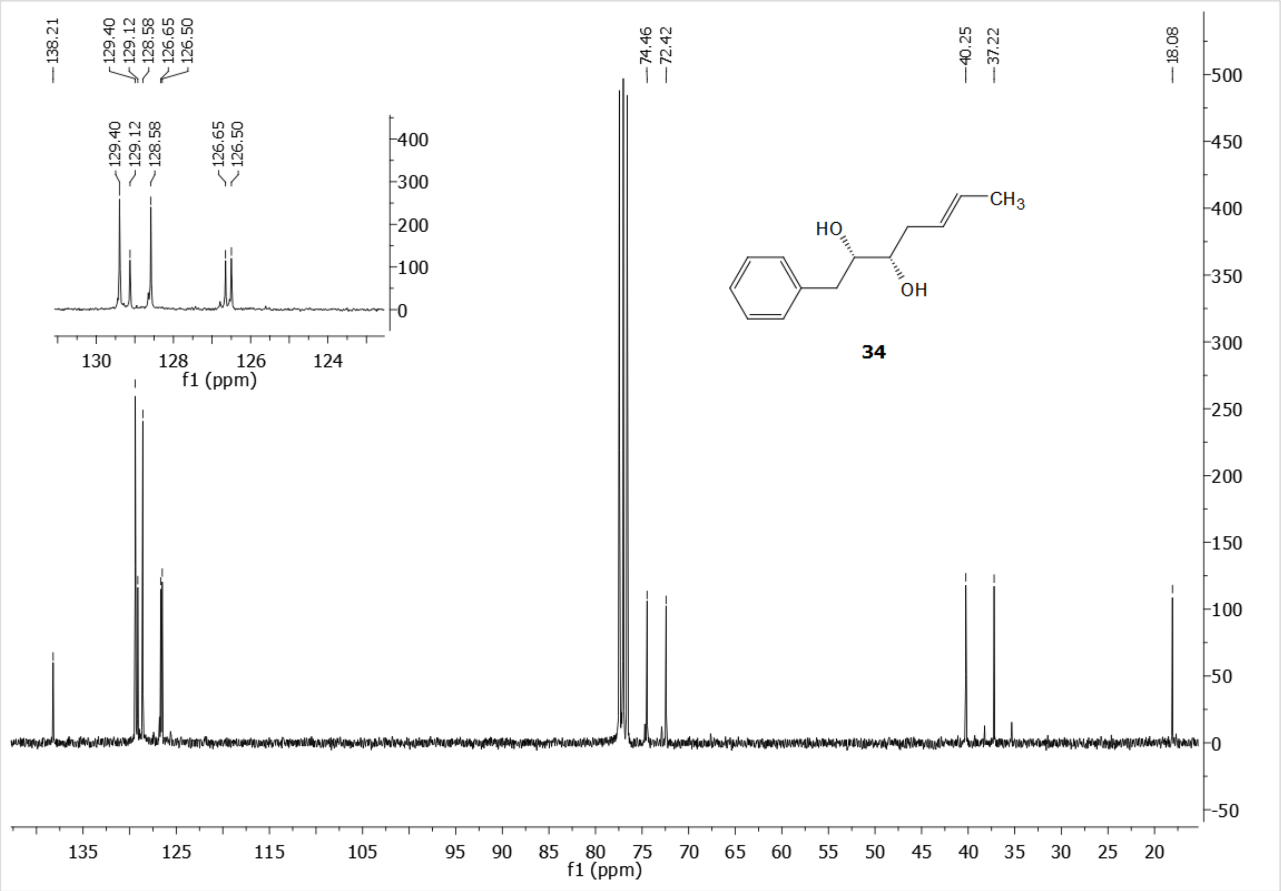
138.21
129.40
129.12
128.58
126.65
126.50
74.46
72.42
40.25
37.22
18.08
HO
OH
CH3
34
f1 (ppm)

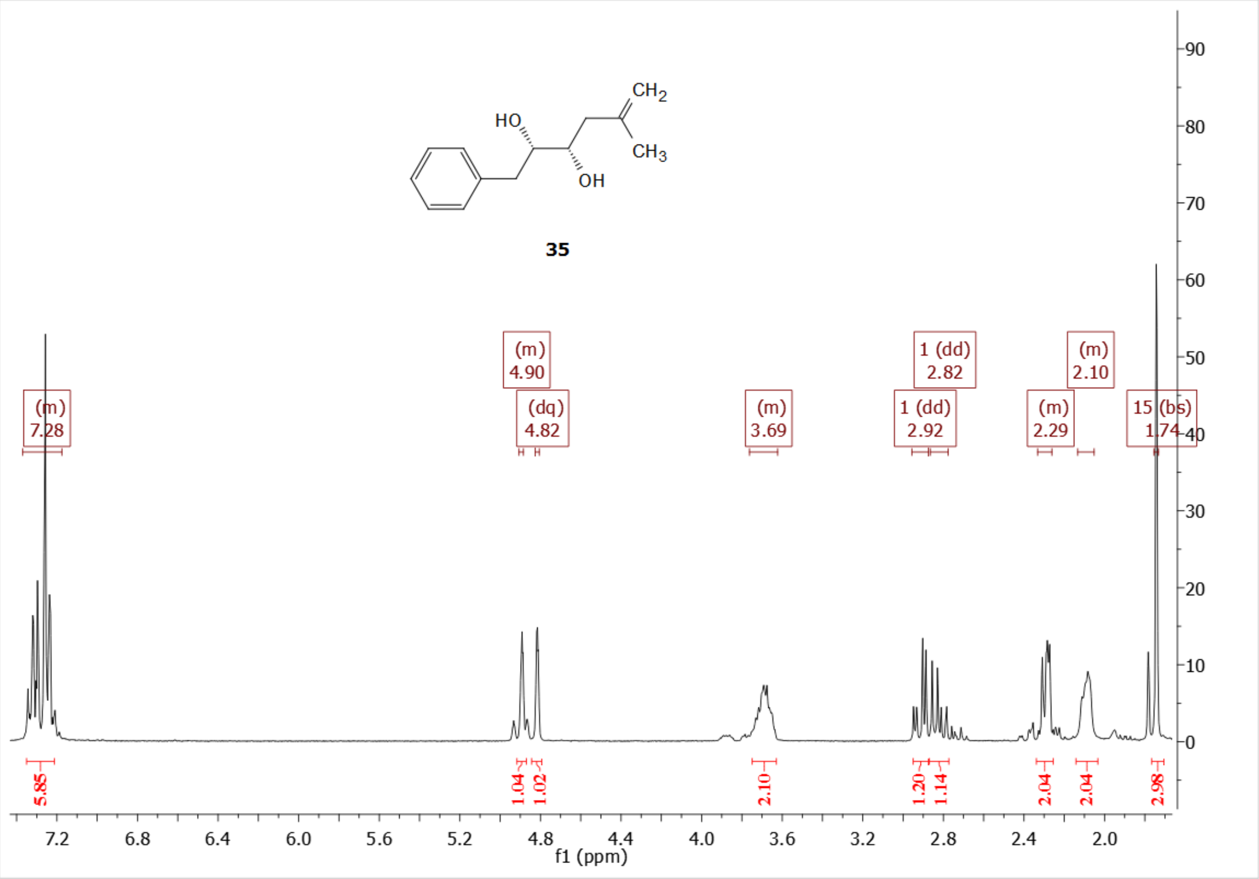
HO
OH
CH2
CH3
35
(m) 7.28
(m) 4.90
(dq) 4.82
(m) 3.69
1 (dd) 2.92
1 (dd) 2.82
(m) 2.29
(m) 2.10
15 (bs) 1.74
5.85
1.04
1.02
2.10
1.20
1.14
2.04
2.04
2.98
f1 (ppm)

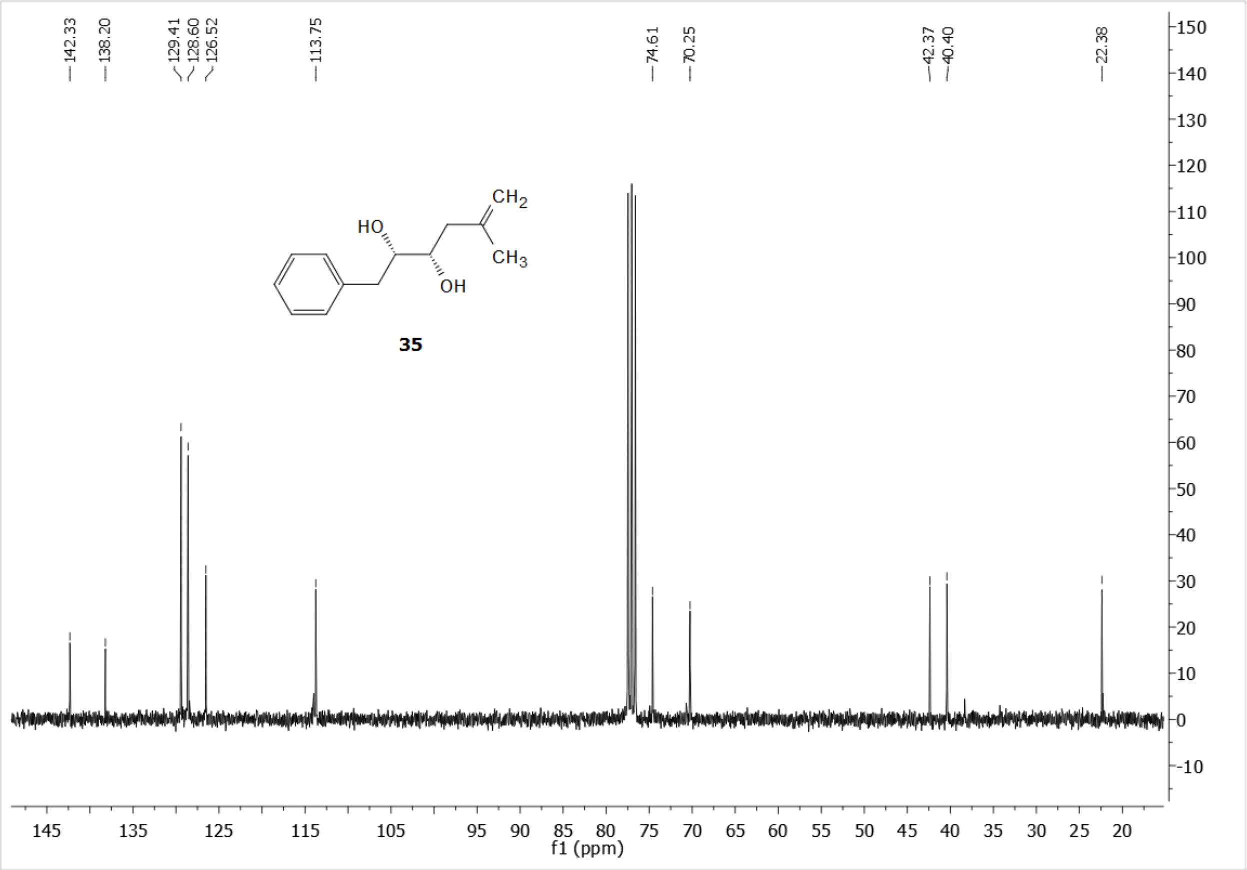
142.33
138.20
129.41
128.60
126.52
113.75
74.61
70.25
42.37
40.40
22.38
HO
OH
CH2
CH3
35
f1 (ppm)

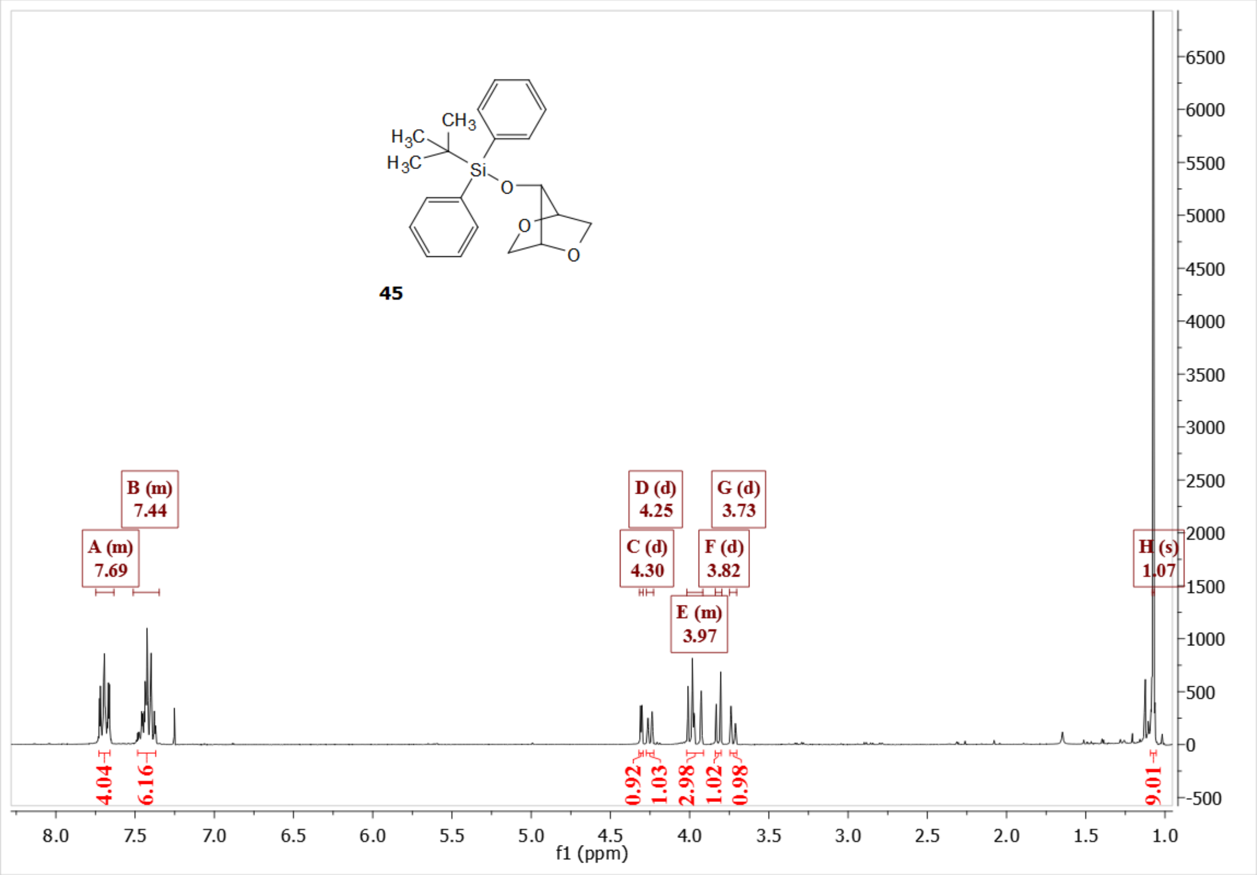

CH3
H3C
H3C
Si
O
O
O
45
A (m)
7.69
B (m)
7.44
C (d)
4.30
D (d)
4.25
E (m)
3.97
F (d)
3.82
G (d)
3.73
H (s)
1.07
4.04
6.16
0.92
1.03
2.98
1.02
0.98
9.01
f1 (ppm)

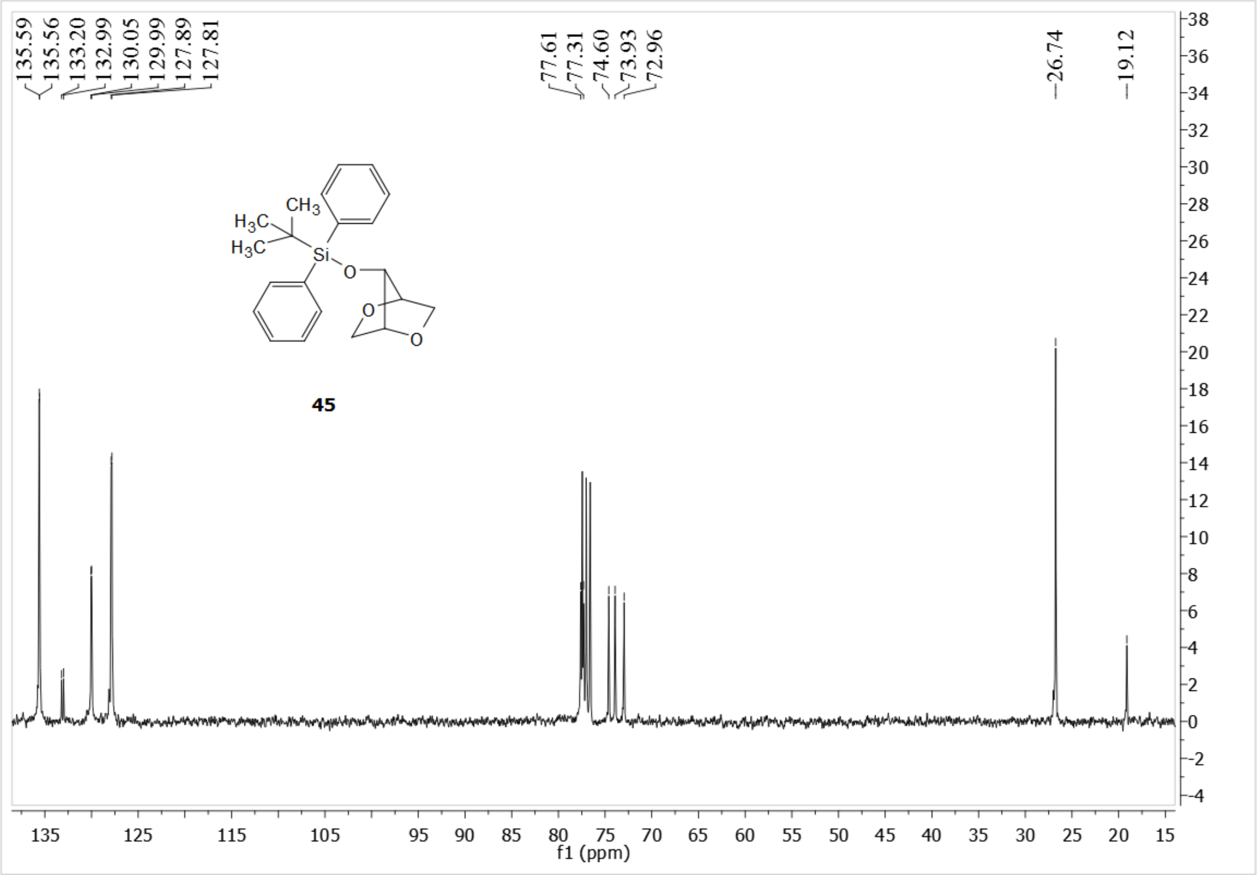

135.59
135.56
133.20
132.99
130.05
129.99
127.89
127.81
77.61
77.31
74.60
73.93
72.96
26.74
19.12
45
f1 (ppm)

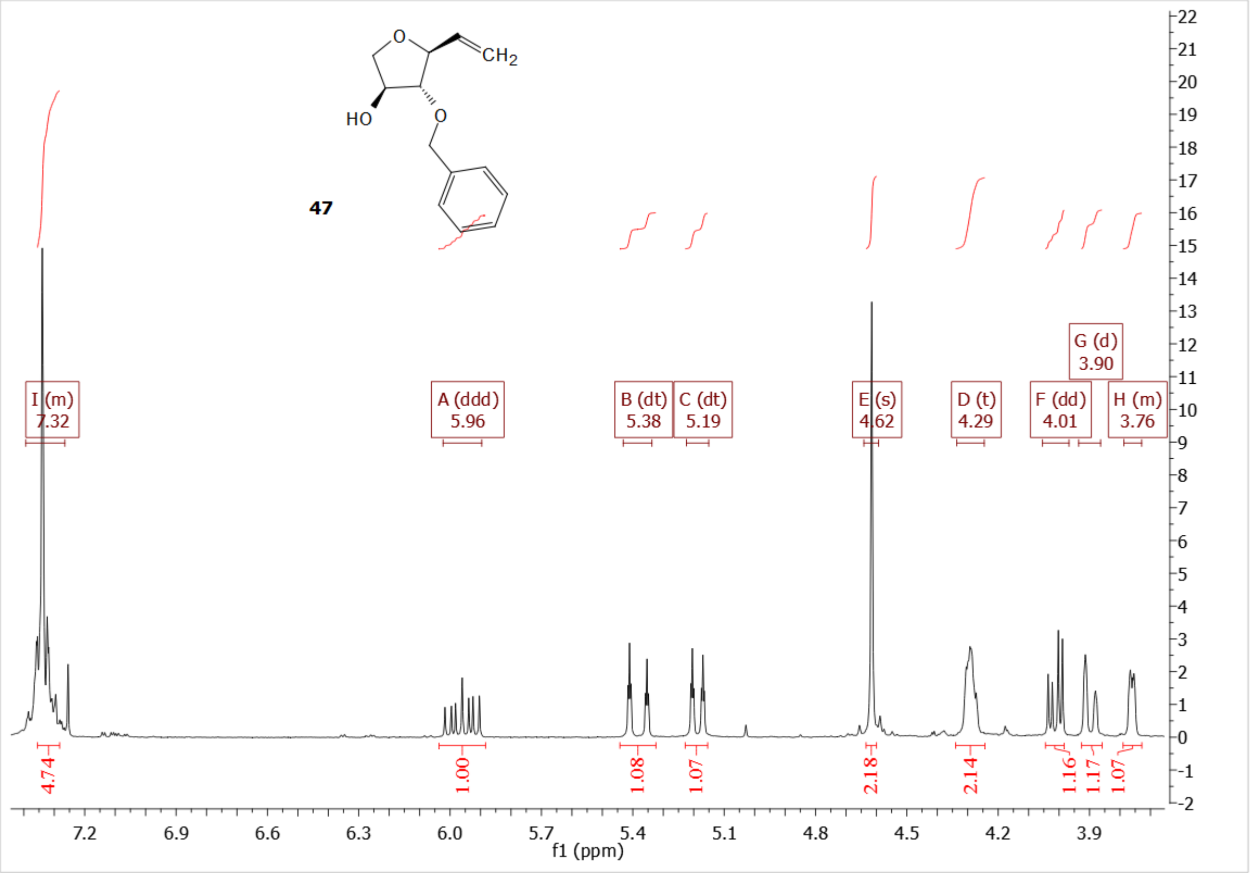

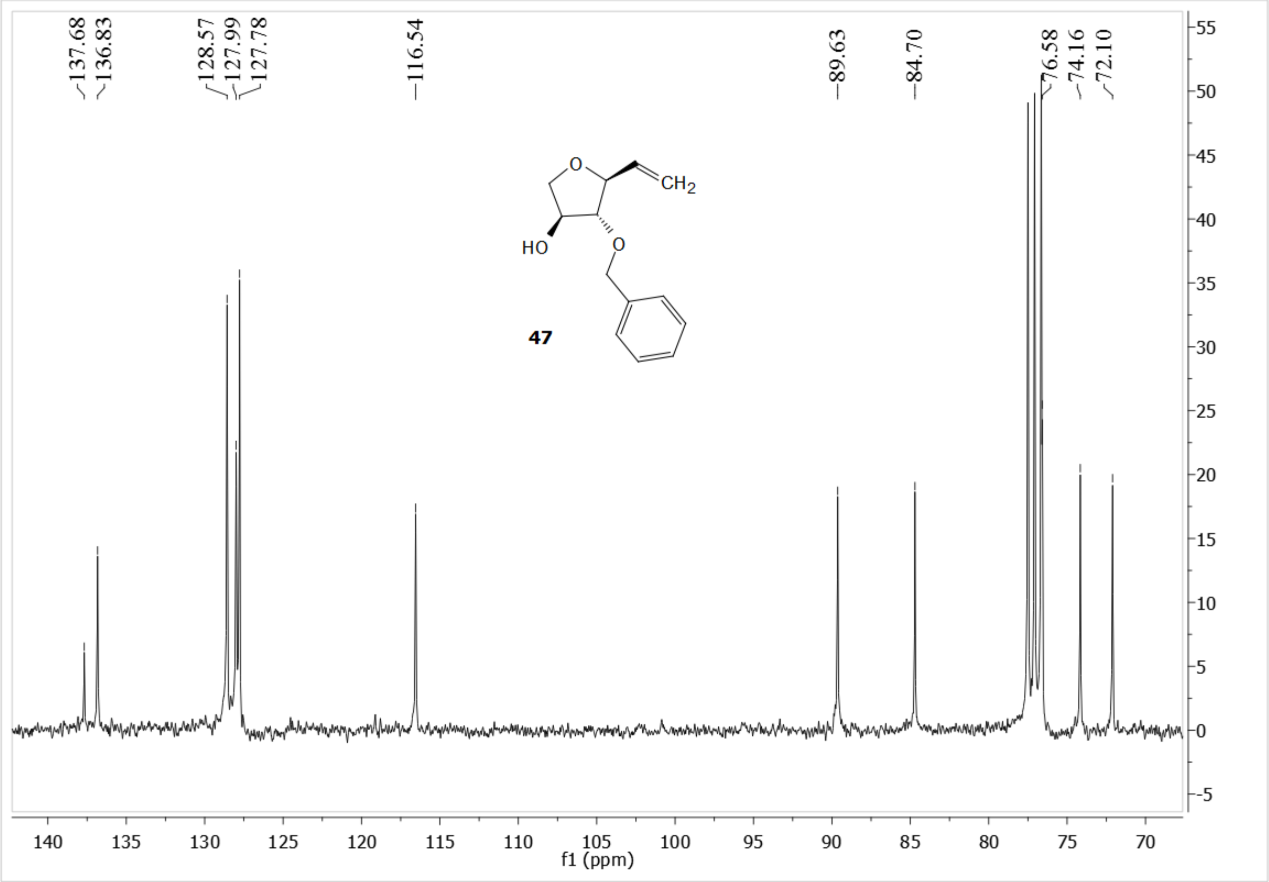

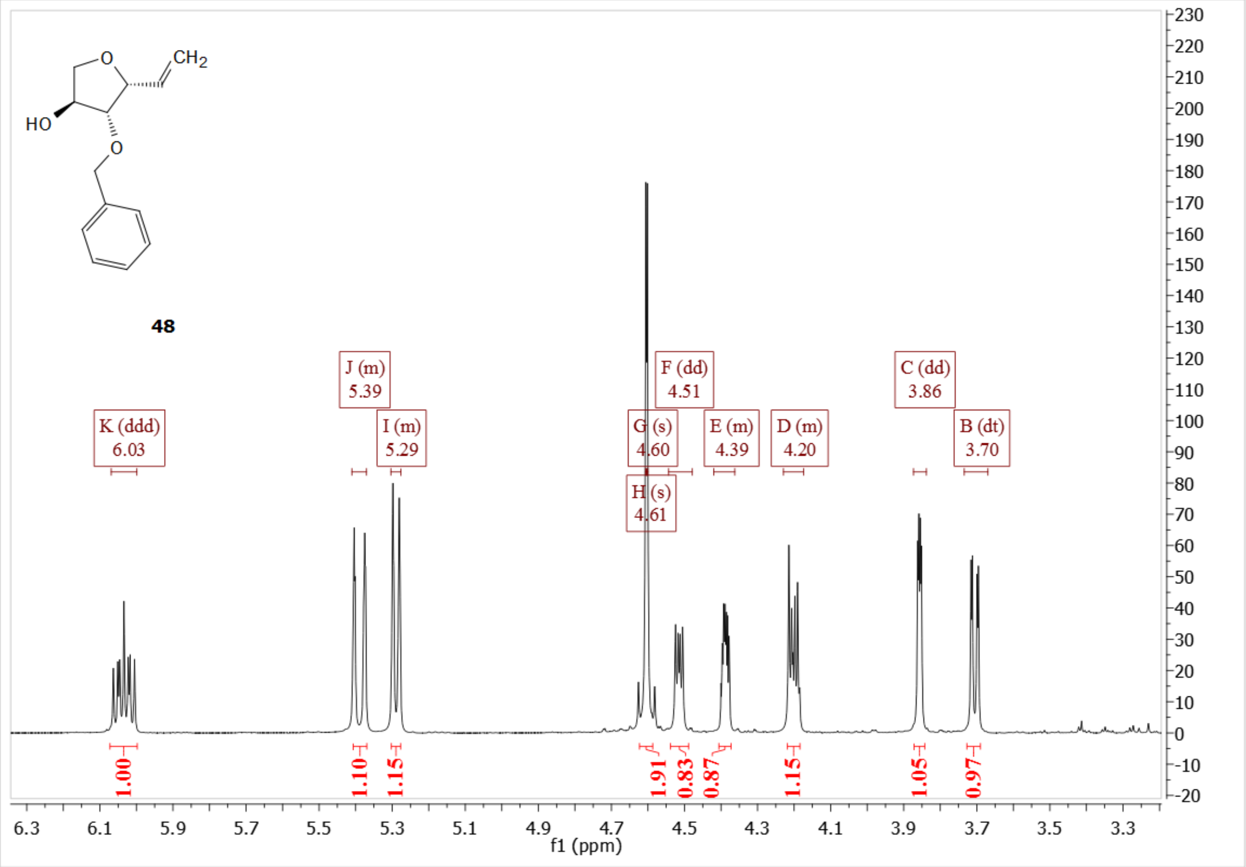
CH2
HO
O
O
48
K (ddd)
6.03
J (m)
5.39
I (m)
5.29
G (s)
4.60
H (s)
4.61
F (dd)
4.51
E (m)
4.39
D (m)
4.20
C (dd)
3.86
B (dt)
3.70
1.00
1.10
1.15
1.91
0.83
0.87
1.15
1.05
0.97
f1 (ppm)

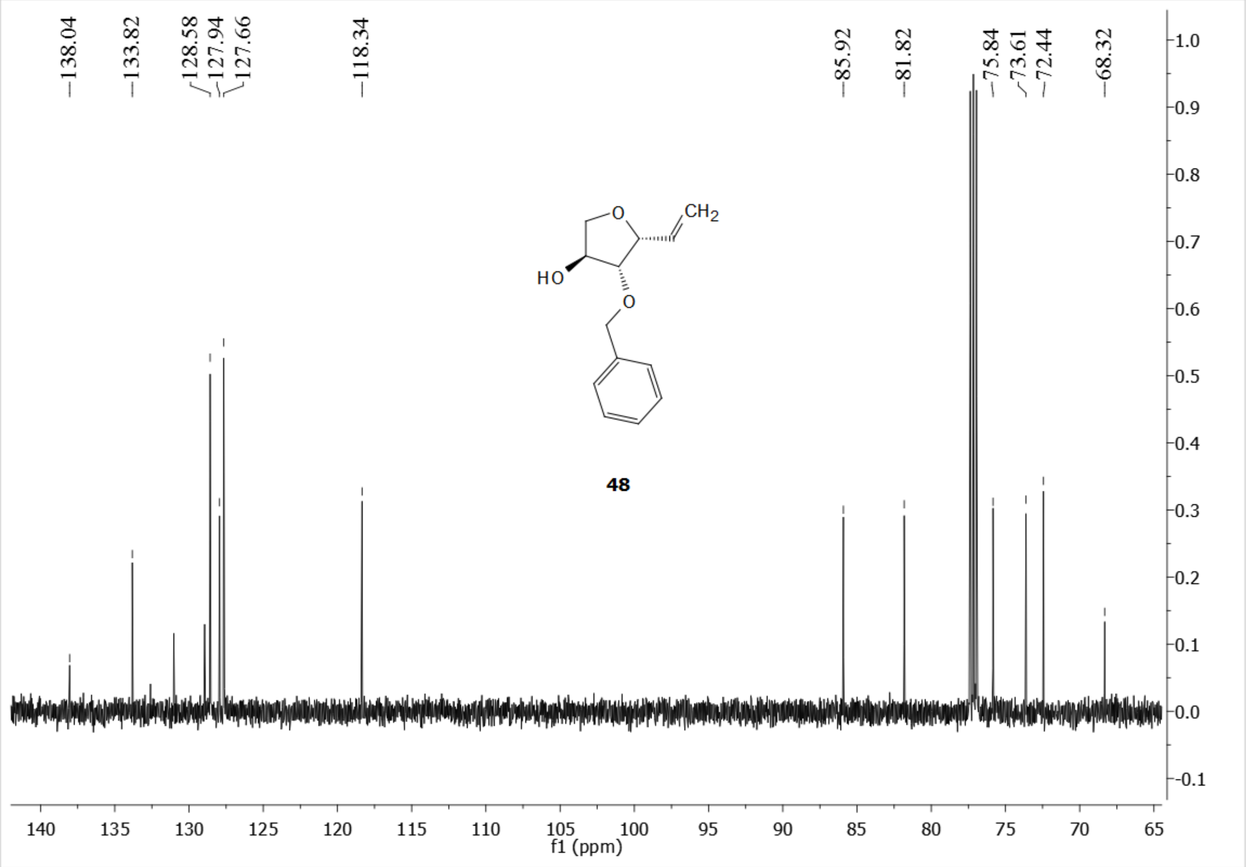
138.04
133.82
128.58
127.94
127.66
118.34
85.92
81.82
75.84
73.61
72.44
68.32
CH2
HO
O
O
48
f1 (ppm)

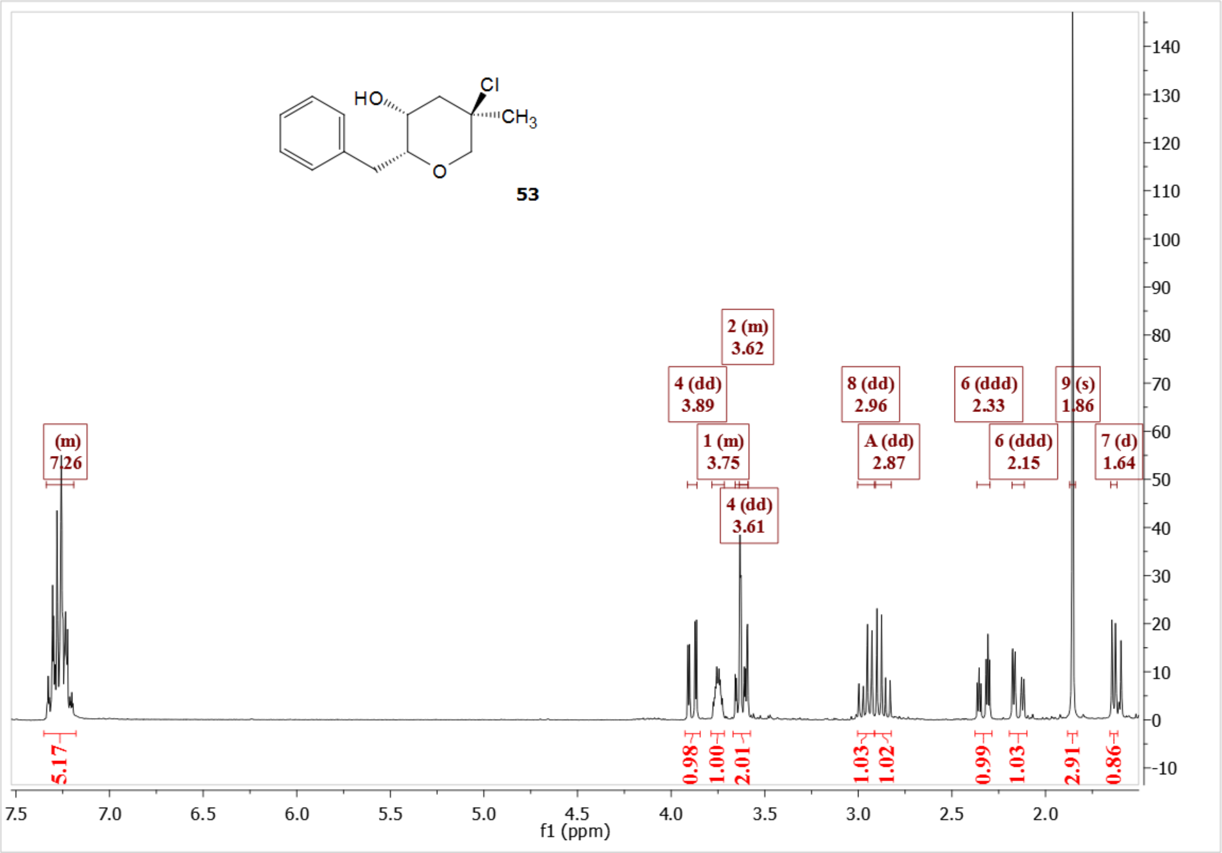

HO
Cl
CH3
O
53
2 (m)
3.62
4 (dd)
3.89
8 (dd)
2.96
6 (ddd)
2.33
9 (s)
1.86
(m)
7.26
1 (m)
3.75
A (dd)
2.87
6 (ddd)
2.15
7 (d)
1.64
4 (dd)
3.61
5.17
0.98
1.00
2.01
1.03
1.02
0.99
1.03
2.91
0.86
f1 (ppm)

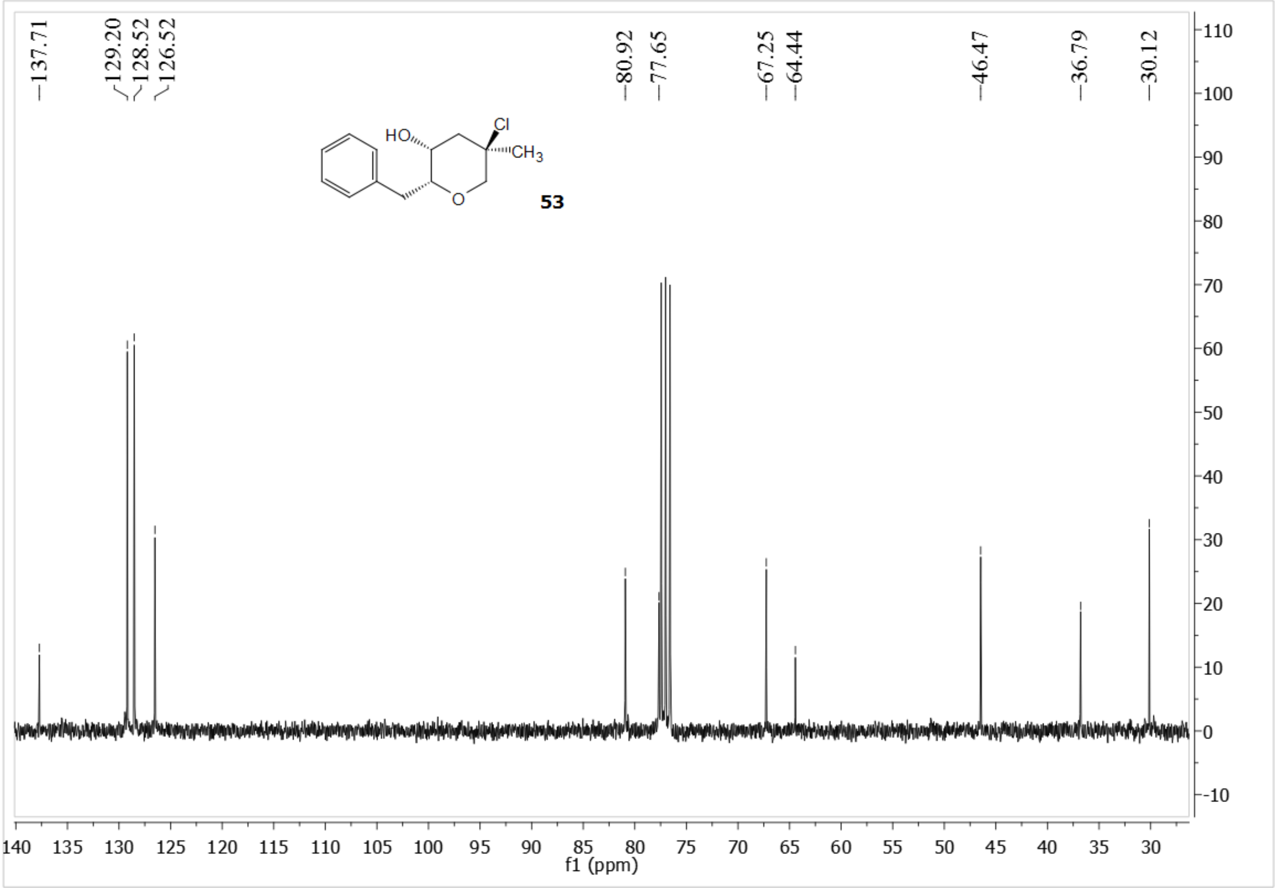

137.71
129.20
128.52
126.52
80.92
77.65
67.25
64.44
46.47
36.79
30.12
HO
Cl
CH3
O
53
f1 (ppm)

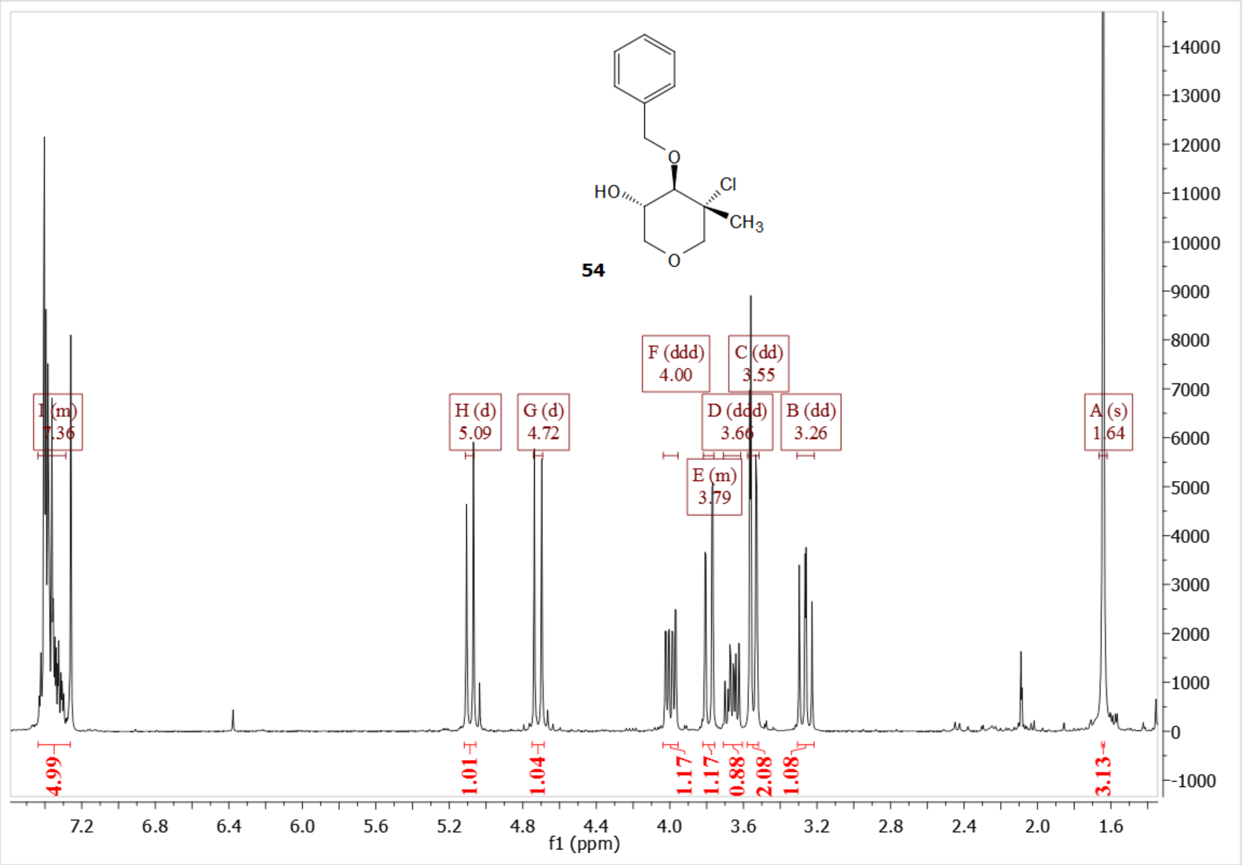
54
HO
Cl
CH3
O
I (m) 7.36
H (d) 5.09
G (d) 4.72
F (ddd) 4.00
E (m) 3.79
D (ddd) 3.66
C (dd) 3.55
B (dd) 3.26
A (s) 1.64
4.99
1.01
1.04
1.17
1.17
0.88
2.08
1.08
3.13
f1 (ppm)

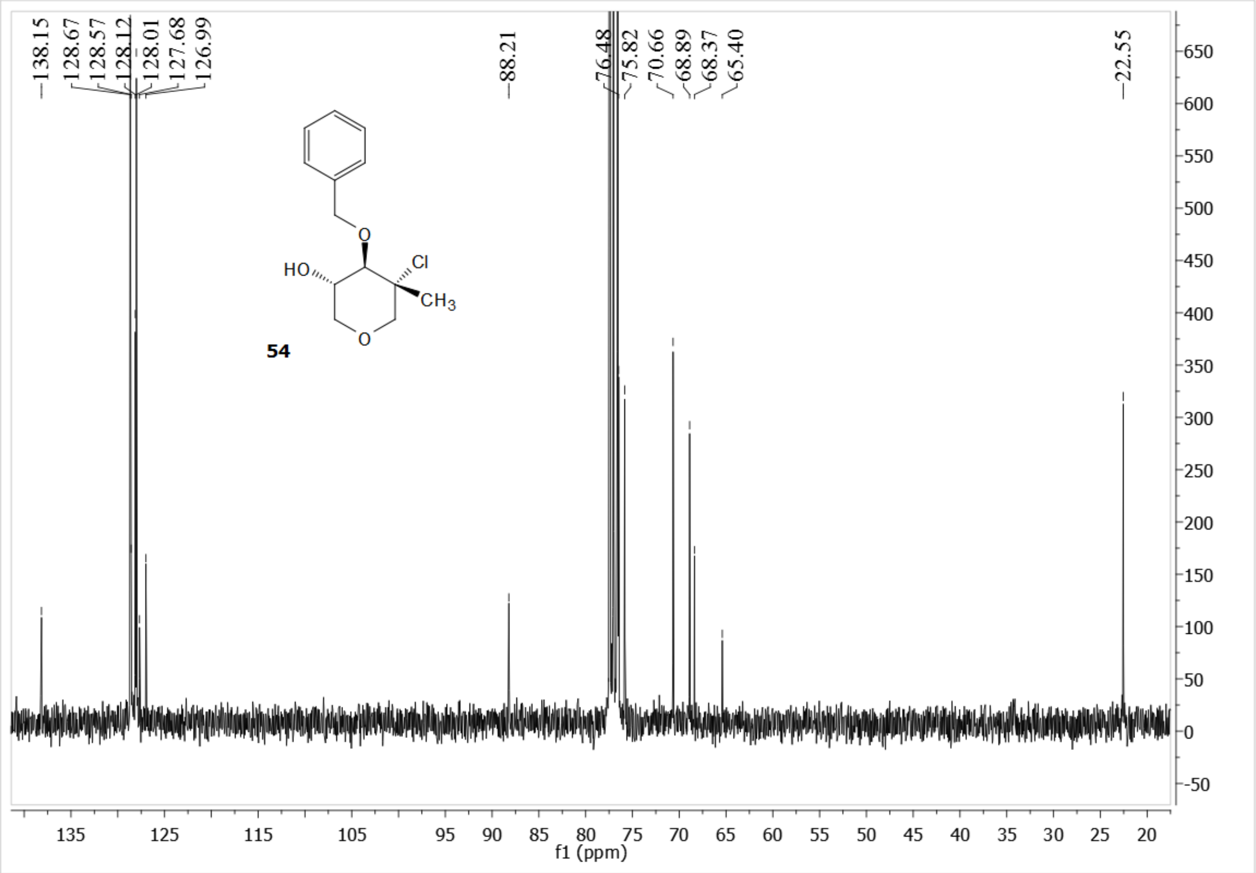
138.15
128.67
128.57
128.12
128.01
127.68
126.99
88.21
76.48
75.82
70.66
68.89
68.37
65.40
22.55
54
HO
Cl
CH3
O
f1 (ppm)

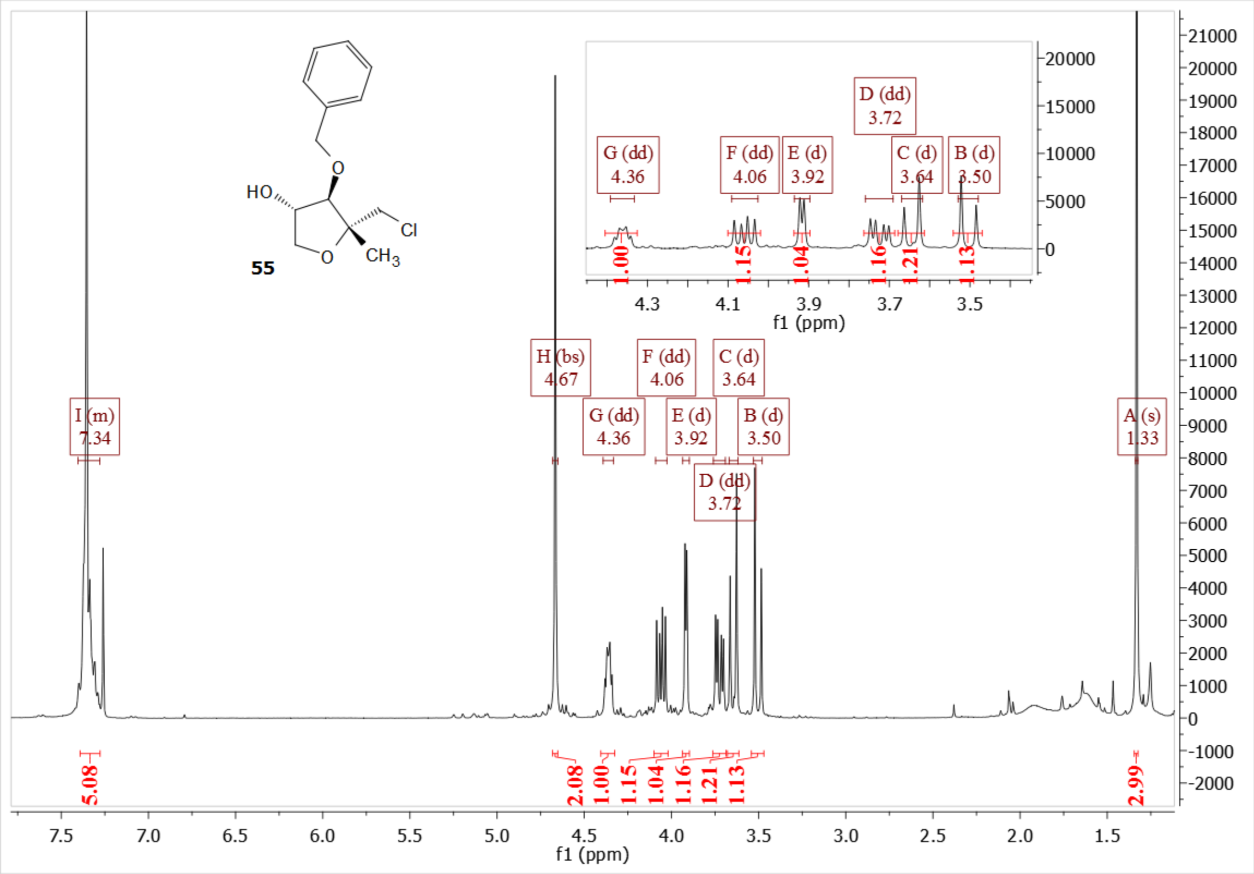

55
HO
O
Cl
CH3
I (m) 7.34
H (bs) 4.67
G (dd) 4.36
F (dd) 4.06
E (d) 3.92
D (dd) 3.72
C (d) 3.64
B (d) 3.50
A (s) 1.33
5.08
2.08
1.00
1.15
1.04
1.16
1.21
1.13
2.99
f1 (ppm)

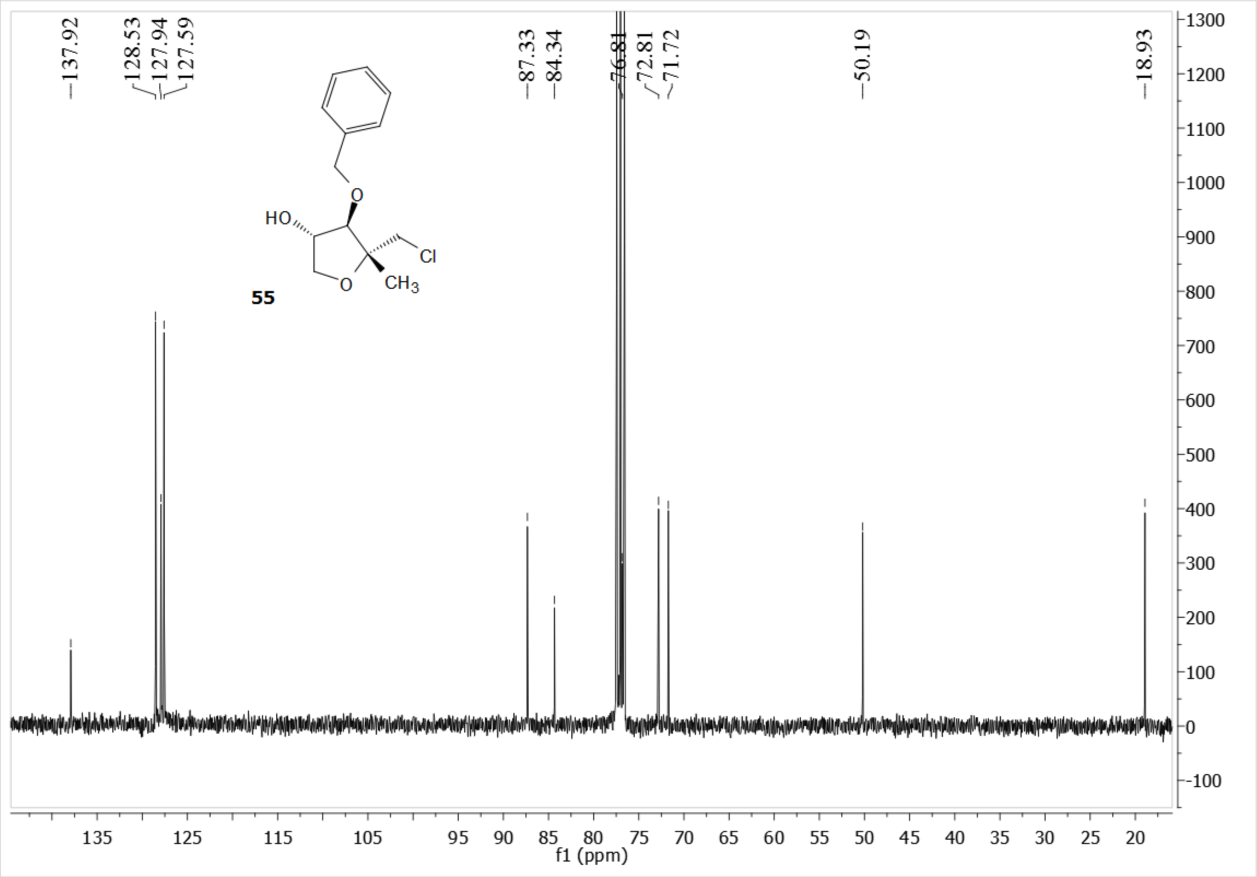

137.92
128.53
127.94
127.59
87.33
84.34
76.81
72.81
71.72
50.19
18.93
55
HO
O
Cl
CH3
f1 (ppm)

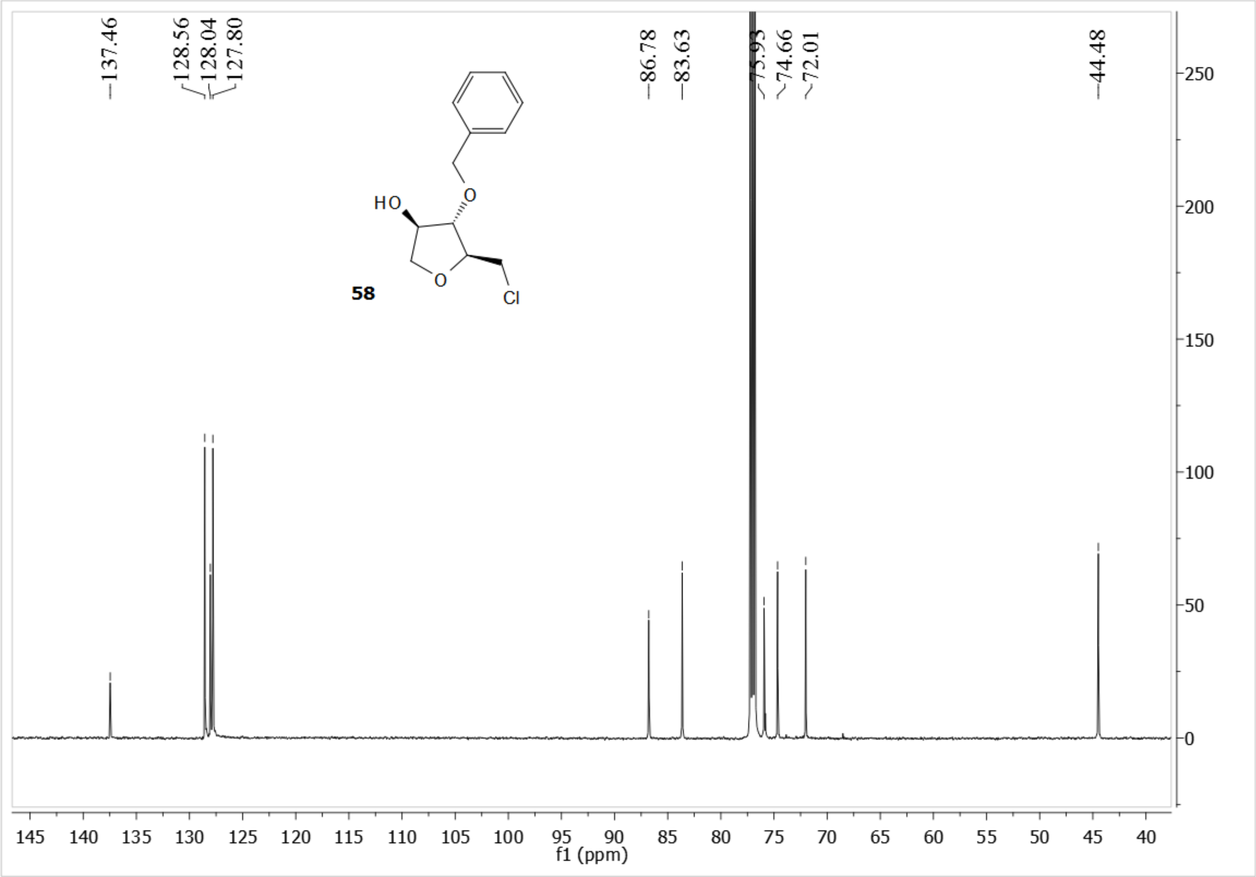

137.46
128.56
128.04
127.80
86.78
83.63
74.66
72.01
44.48
HO
O
O
Cl
58
f1 (ppm)

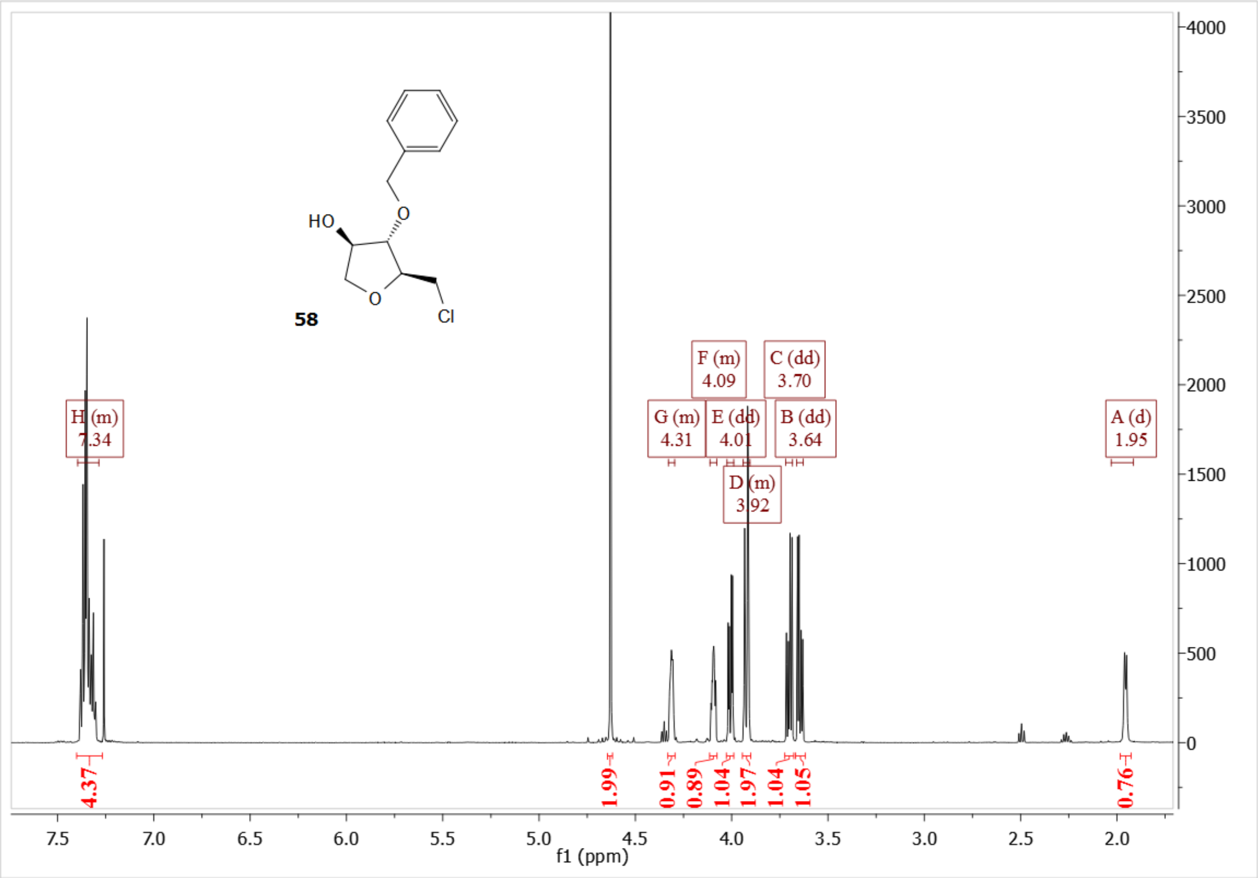

HO
O
O
Cl
58
H (m)
7.34
G (m)
4.31
F (m)
4.09
E (dd)
4.01
D (m)
3.92
C (dd)
3.70
B (dd)
3.64
A (d)
1.95
4.37
1.99
0.91
0.89
1.04
1.97
1.04
1.05
0.76
f1 (ppm)

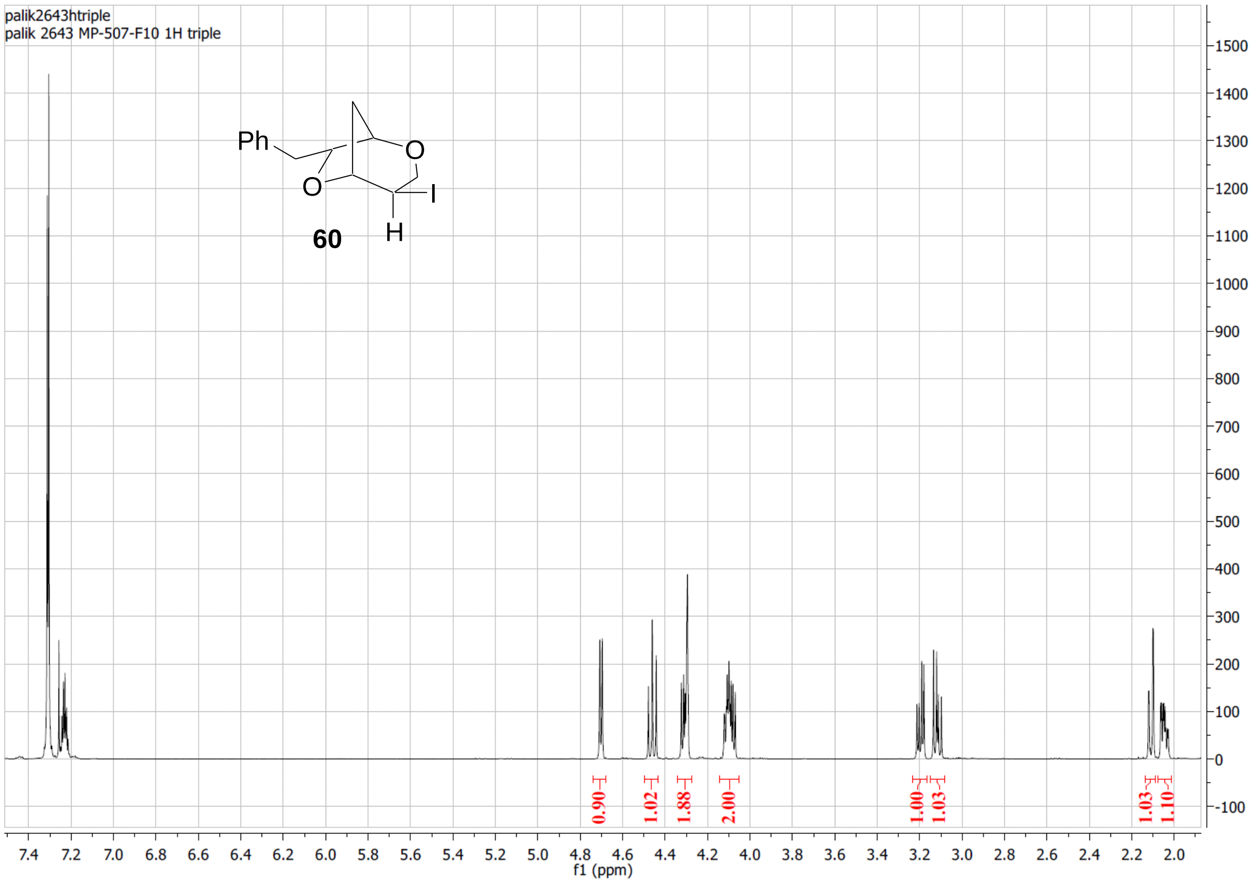
palik2643htriple
palik 2643 MP-507-F10 1H triple
Ph
O
O
I
H
60
0.90
1.02
1.88
2.00
1.00
1.03
1.03
1.10
f1 (ppm)

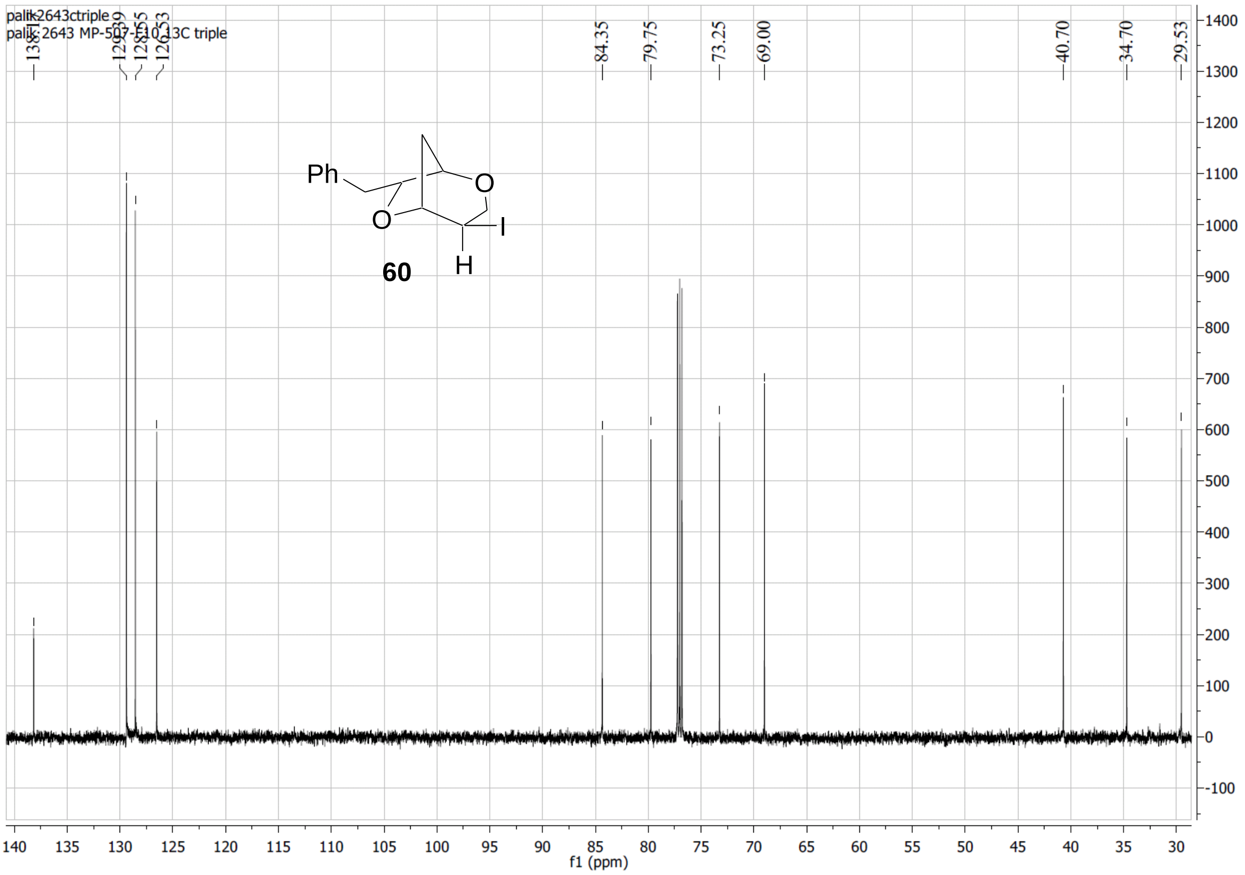
palik2643ctriple
palik 2643 MP-507-F10 13C triple
84.35
79.75
73.25
69.00
40.70
34.70
29.53
Ph
O
O
I
H
60
f1 (ppm)

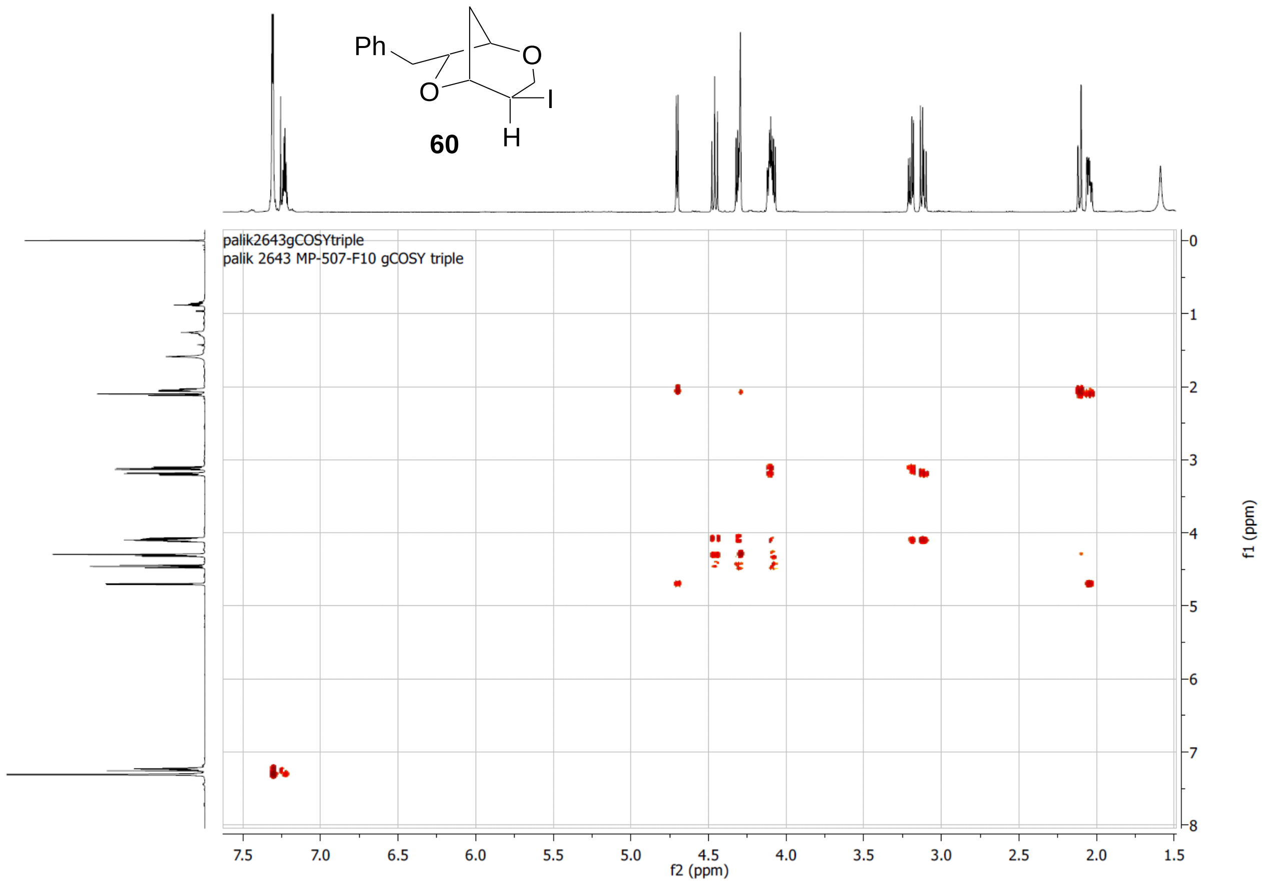
Ph
O
O
I
H
60
palik2643gCOSYtriple
palik 2643 MP-507-F10 gCOSY triple
f2 (ppm)
f1 (ppm)

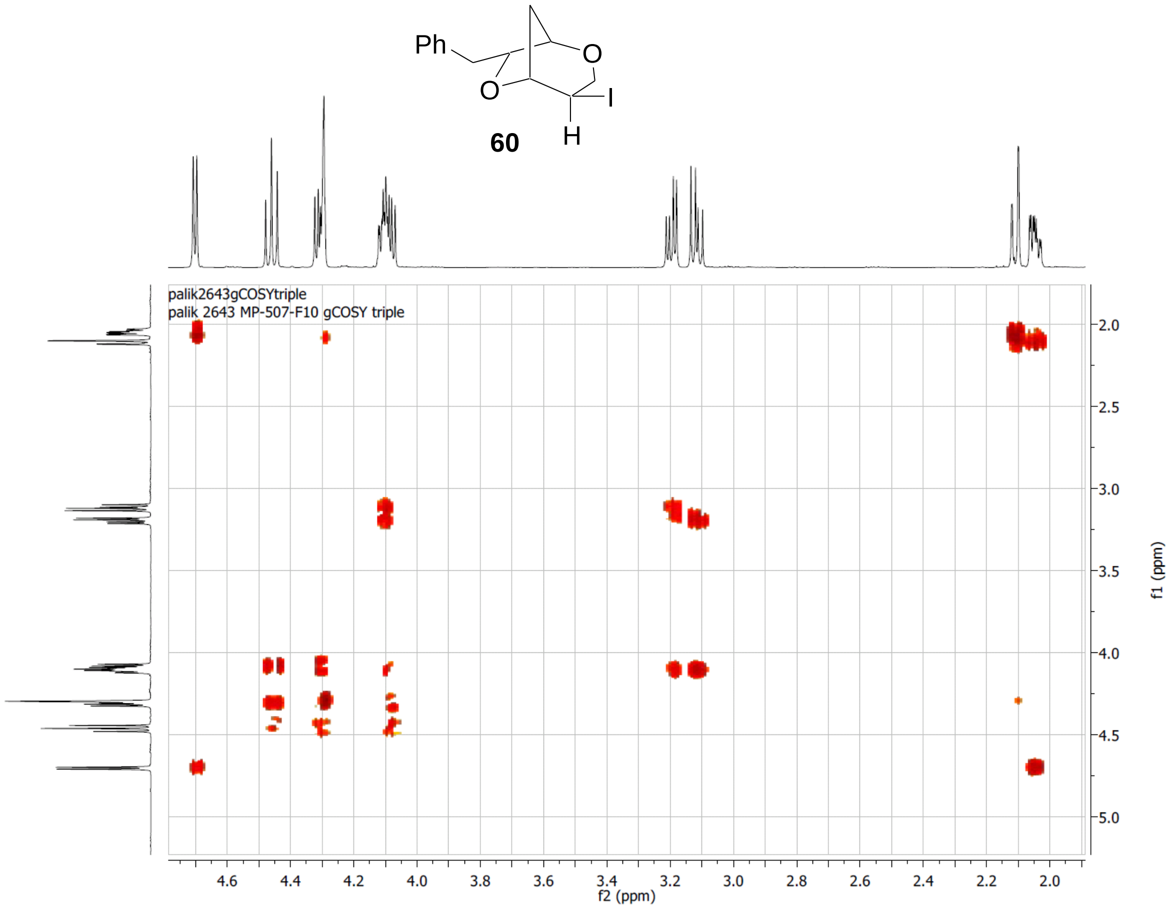
Ph
O
O
I
H
60
palik2643gCOSYtriple
palik 2643 MP-507-F10 gCOSY triple
f2 (ppm)
f1 (ppm)

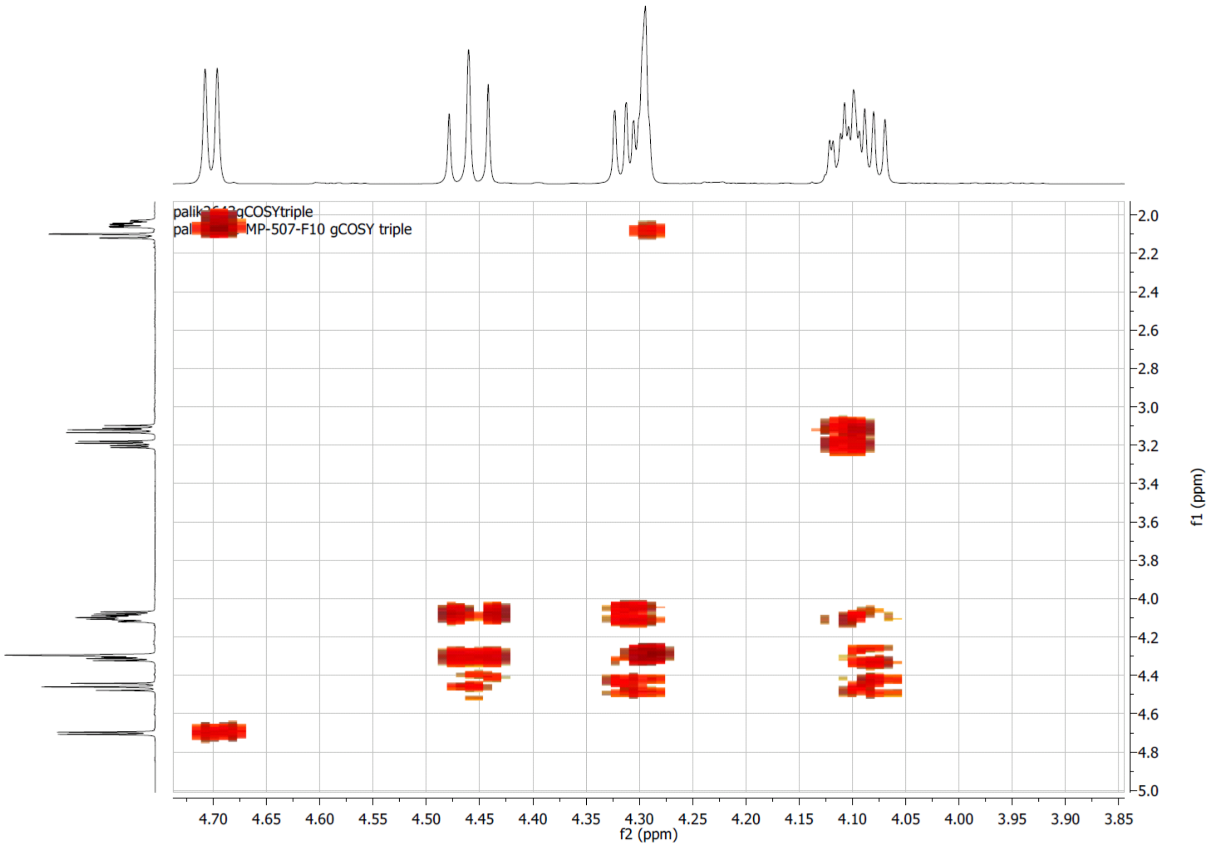
MP-507-F10 gCOSY triple
f2 (ppm)
f1 (ppm)

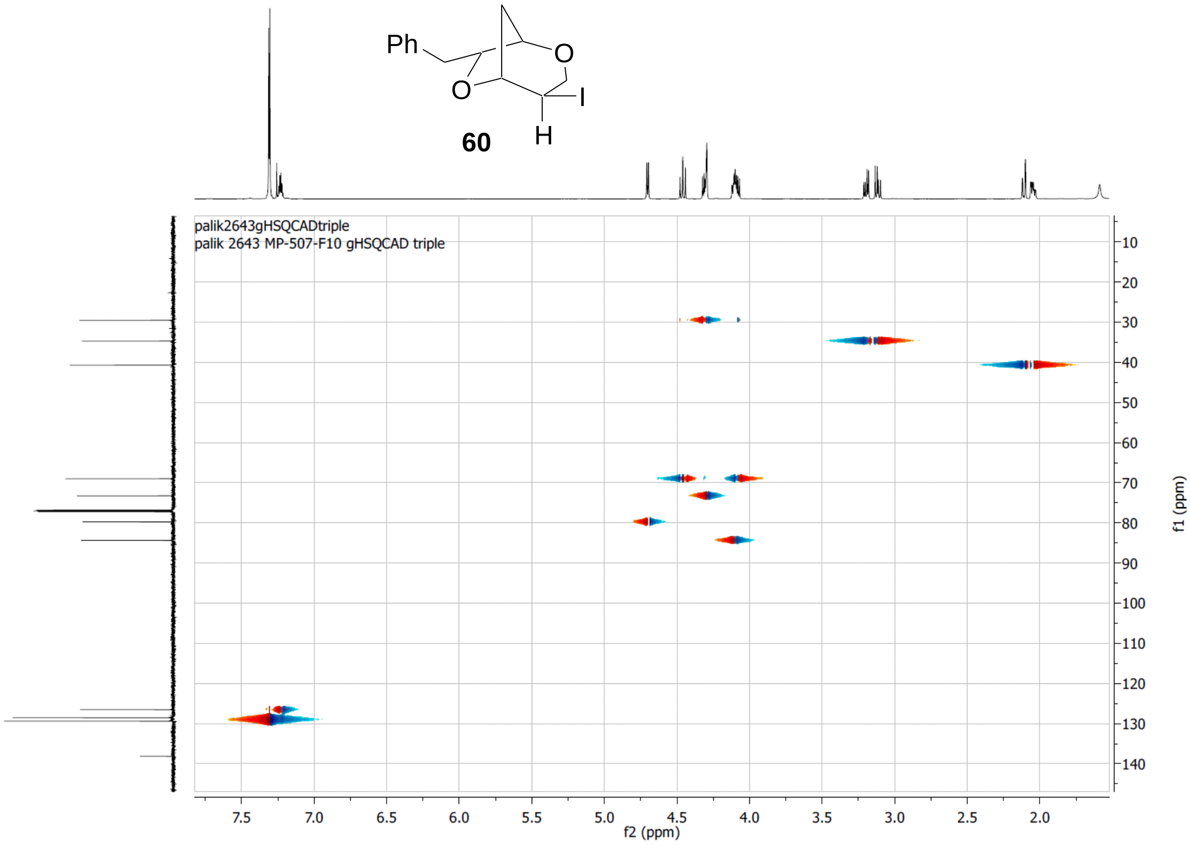
Ph
O
O
I
H
60
palik2643gHSQCADtriple
palik 2643 MP-507-F10 gHSQCAD triple
f2 (ppm)
f1 (ppm)

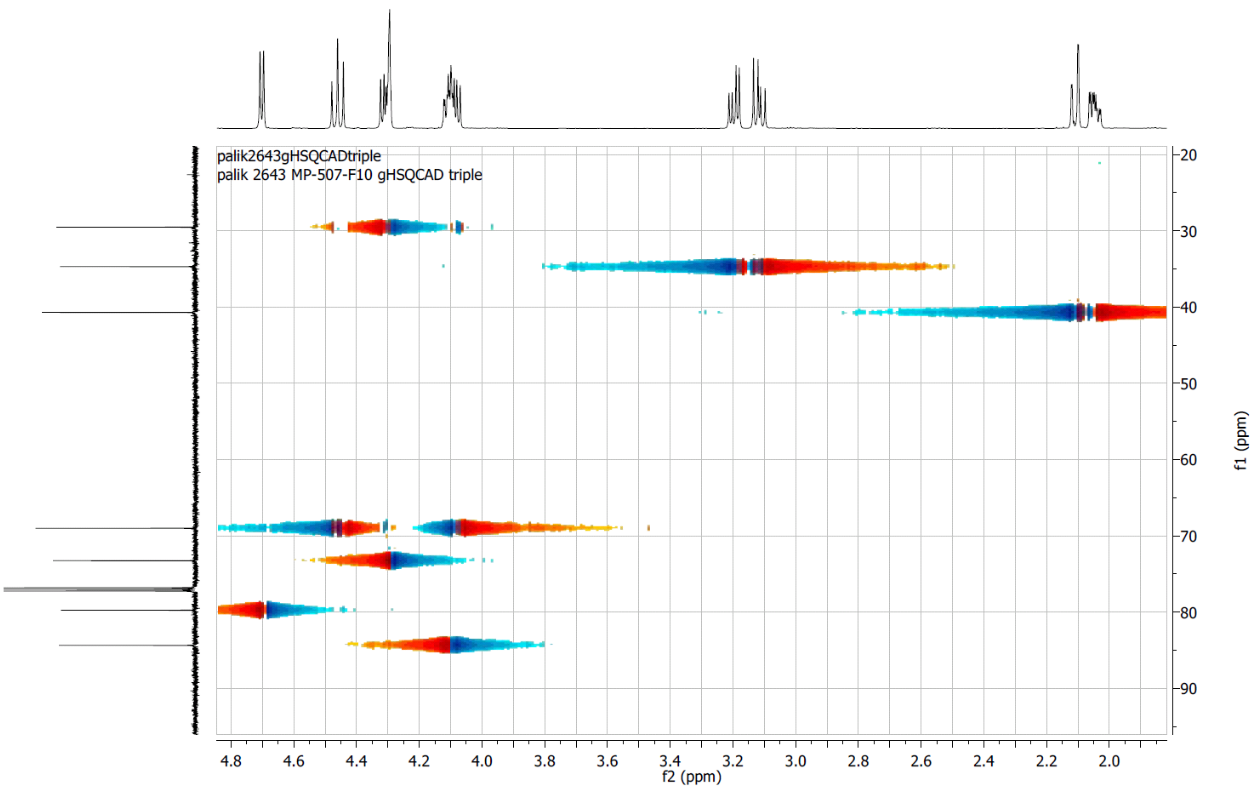
palik2643gHSQCADtriple
palik 2643 MP-507-F10 gHSQCAD triple
f2 (ppm)
f1 (ppm)

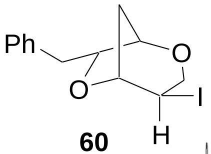
Ph
O
O
I
H

60

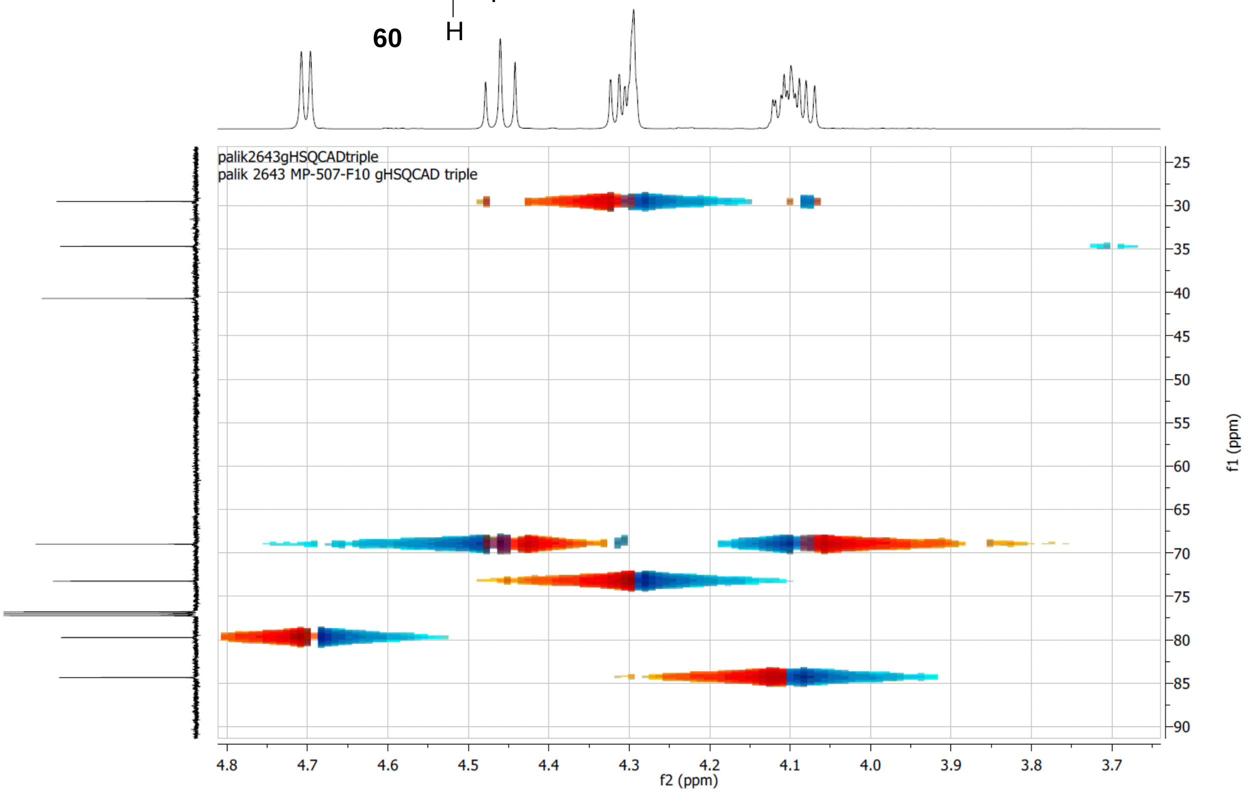
palik2643gHSQCADtriple
palik 2643 MP-507-F10 gHSQCAD triple
f2 (ppm)
f1 (ppm)
