# Supplementary material for: Palladium-catalysed cyclisation of alkenols: Synthesis of oxaheterocycles as core intermediates of natural compounds
Source: Beilstein J Org Chem. 2014 Sep 3;10:2077–86. doi: 10.3762/bjoc.10.216 (PMC4168889; doi:10.3762/bjoc.10.216)
Supplement: File 3 — X-ray crystal structure analysis of 53. [file Beilstein_J_Org_Chem-10-2077-s003.pdf]

Supporting Information File 3

for

Palladium-catalysed cyclisation of alkenols: Synthesis of oxaheterocycles as core intermediates of natural compounds

Miroslav Palík[1], Jozef Kožíšek[2], Peter Koóš[3] and Tibor Gracza*[1]

Address: [1]Department of Organic Chemistry Slovak University of Technology, Radlinského 9, SK-812 37 Bratislava, Slovakia, [2]Department of Physical Chemistry, Slovak University of Technology, Radlinského 9, SK-812 37 Bratislava, Slovakia and[3]Georganics Ltd., Koreničova 1, SK-811 03 Bratislava, Slovakia

Email: Tibor Gracza - tibor.gracza@stuba.sk

* Corresponding author

X-ray Crystal Structure Analysis of 53

Single crystals of **53** suitable for X-ray crystal analysis were used for data collection at 100 K on a Oxford Diffraction GEMINI R diffractometer[1] equipped with an CCD detector with Mo K_{α} radiation (λ= 0.71070 Å) and graphite monochromator. Crystal structure was solved and refined by SHELXS and SHELXL-97 suit of programs.[2]

[1] Oxford Diffraction (2008). *CrysAlis CCD* and *CrysAlis RED*. Oxford Diffraction Ltd, Abingdon, Oxfordshire, England.

[2] Sheldrick, G. M. (1997). SHELXS97and SHEXL97, University of Göttingen, Germany.

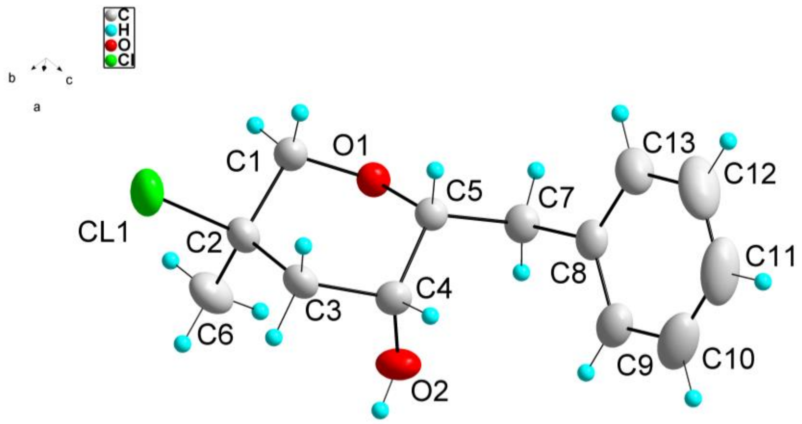

Figure 1. ORTEP[3] scheme of **53.** Thermal ellipsoids are drawn at the 20% probability level.

[3] Brandenburg, K. (1998). *DIAMOND*. Visual Information System for Crystal Structures, Bonn, Germany.

Table 1 Crystallographic data and structure refinement details for **53**

Formula	$C_{13}H_{17}ClO_2$
Formula weight	240.71
Temperature (K)	298 (2)
Wavelength λ (Å)	0.71073
Crystal system	orthorhombic
Space group	$P\ 2_12_12_1$
Unit cell dimensions	
a (Å)	8.9957(2)
b (Å)	9.7725(3)
c (Å)	14.4036(4)
α (°)	90.0
β (°)	90.0
γ (°)	90.0
Volume (Å^3)	1266.23(6)
Z	4
D_{calc} (g·cm^{-3})	1.263
μ (mm^{-1})	0.285
F(000)	512
Reflections collected	21581
Independent reflections	2592
Refinement on F^2	
R_{int}	0.0129
S	1.065
$R[F^2>2\sigma(F^2)]$	0.0327
$wR(F^2)$	0.0796
$wR(F^2)$[a]	0.0824

[a] All diffractions

Table 2a Selected bond lengths (Å) and angles (°)

	**53**
Bond lengths (Å)	
C1-O1	1.434 (3)
C1-C2	1.516 (3)
C2-C3	1.520 (3)
C3-C4	1.526 (3)
C4-C5	1.516 (3)
C5-O1	1.438 (3)
C2-Cl1	1.830 (2)
C5-C7	1.521 (3)
C7-C8	1.501 (3)
Bond angles (°)	
O1—C1—C2	109.4 (2)
C1—C2—C3	109.5 (2)
C2—C3—C4	112.6(2)
C3—C4—C5	109.9(2)
C4—C5—O1	110.1(2)
C1—O1—C5	111.2(2)
C1—C2—Cl1	105.1(2)
C3—C2—Cl1	107.3(2)

Table 2b Hydrogen bonds

D-H…A	d(D-H)	d(H...A)	d(D...A)	<(DHA)
C5—H5*A*···Cl1 [i]	1.00	2.81	3.778 (2)	163
C6—H6*B*···O2	0.98	2.54	3.117 (4)	118
O2—H2*A*···O1 [ii]	0.82 (4)	2.04 (4)	2.850 (2)	171 (3)

Symmetry code used: [i] $-x+2, y-1/2, -z+1/2$; [ii] $x+1/2, -y+1/2, -z+1$.

Supplementary material: Crystallographic data for the structural analysis have been deposited at the Cambridge Crystallographic Data Centre, CCDC no. **963459**.
